# Euphraticanoids N–T: Aromadendrane-Type Diterpenes and Sesquiterpenes with Fungicidal Activities from *Populus euphratica* Resins

**DOI:** 10.3390/ijms26052187

**Published:** 2025-02-28

**Authors:** Qinbin Jiang, Yun-Yun Liu, Danling Huang, Yong-Xian Cheng

**Affiliations:** 1 School of Pharmacy, Guangdong Pharmaceutical University, Guangzhou 510006, China; jqb0925@163.com; 2 Guangdong Provincial Key Laboratory of Chinese Medicine Ingredients and Gut Microbiomics, Institute for Inheritance-Based Innovation of Chinese Medicine, School of Pharmacy, Shenzhen University, Shenzhen 518055, China; liuyy@szu.edu.cn (Y.-Y.L.); leonchemistry@szu.edu.cn (D.H.)

**Keywords:** *Populus euphratica* resins, prenylaromadendrane-type diterpenes, aromadendrane-type sesquiterpenes, fungicidal activity

## Abstract

Seven previously undescribed terpenoids, including five prenylaromadendrane-type diterpenes euphraticanoids N–R (**1**–**5**) and two aromadendrane-type sesquiterpenes, euphraticanoids S and T (**6** and **7**), were isolated from *Populus euphratica* resins. Their structures, including their absolute configurations, were elucidated by HRESIMS and spectroscopic analysis, ECD calculations, and crystallographic methods. In addition, an evaluation of the fungicidal activities of compound **1** was carried out, resulting in the discovery of **1** as a fungicidal candidate lead compound with an EC_50_ of 15.7 and 68.6 mg/L against *Curvularia mebaldsii* and *Fusarium graminearum*, respectively.

## 1. Introduction

**Figure 1:**
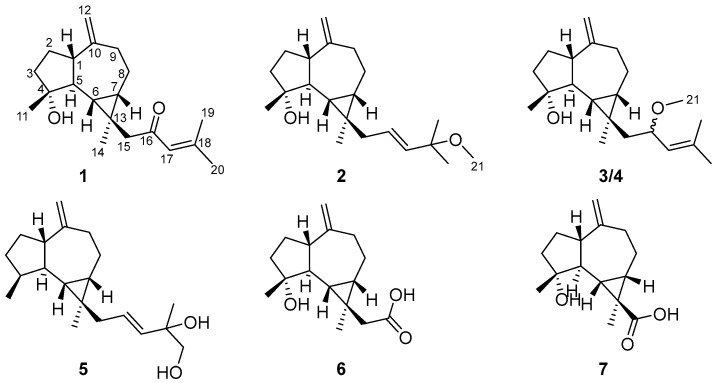
The chemical structures of compounds **1**–**7**.

All living creatures, including humans, animals, and plants, have developed diverse defense strategies though evolution for self-protection, as they face extreme stresses such as environmental stresses and social stresses. Among various defense mechanisms, exudate production plays an important role. For example, humans shed tears when they get hurt; the hagfish produces slime when it is provoked [1]; sperm whales create ambergris when they eat hard squid beak chitin, an irritant [2]; and *Megaponera analisantis* ants secrete saliva to treat infected wounds when nestmates are infected [3]. However, the phenomenon of resinous exudate production to defend against injury is more common in plants than humans and animals, such as agarwood from the resinous heartwood of *Aquilaria* tree [4]; red resin from the fruits of *Daemonorops draco* tree [5]; yellowish resins from *Ferula sinkiangensis* [6], and so on. We assumed that these plants’ resinous exudates are used to defend, and should have biological activities, which also suggested that these resins may be a potentially valuable reservoir in drug discovery. Therefore, our group has focused on plant resins in recent years. A recent comprehensive review by us summarized the chemistry and biological activity of recent substances, highlighting our contribution to this field [7]. The tears of *Populus euphratica* were collected for systematic research by us, accompanied by discovering a series of structurally intriguing and biologically significant compounds, including various terpenoids [8]. As a continuous study on this topic, five prenylaromadendrane-type diterpenes euphraticanoids N–R (**1**–**5**) and two aromadendrane-type sesquiterpenes euphraticanoids S and T (**6** and **7**) were identified (Figure 1). In addition, we evaluated the fungicidal activity of compounds **1**–**7**. As a result, compound **1** was found to exhibit potent fungicidal activity against *Curvularia mebaldsii* and *Fusarium graminearum*. This finding provides hope for the discovery of fungicides derived from *P. euphratica.*

## 2. Results and Discussion

### 2.1. Compound Structure Elucidation

**Figure 2:**
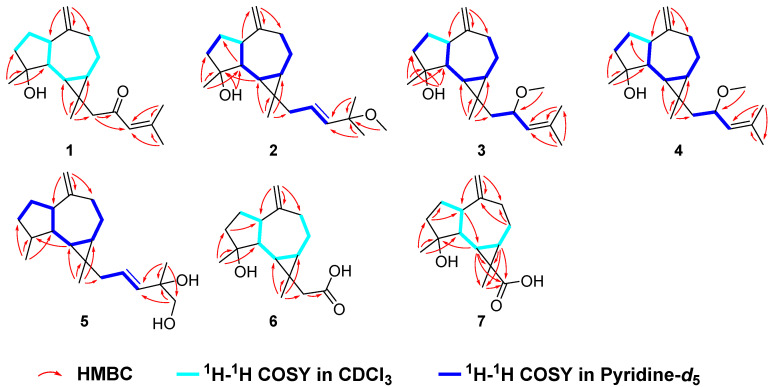
^1^H-^1^H COSY and key HMBC correlations of **1**–**7**.

Euphraticanoid N (**1**) was obtained as yellowish crystals through crystallization of methanol, and its molecular formula C_20_H_30_O_2_ was supported by HRESIMS (*m*/*z* 303.2311 [M + H]^+^, calcd for C_20_H_31_O_2_, 303.2319), ^13^C NMR, and DEPT spectra. The ^1^H NMR spectrum (Table 1) showed signals for four methyl groups at *δ*_H_ 2.14 (3H, d, *J* = 0.8 Hz), 1.87 (3H, d, *J* = 0.8 Hz), 1.24 (3H, s), and 1.03 (3H, s), and three olefinic protons at *δ*_H_ 6.00 (1H, brs), 4.73 (1H, brs), and 4.69 (1H, brs), along with two characteristic protons at *δ*_H_ 0.60 (1H, t-like, *J* = 11.0 Hz) and 0.77 (1H, td, *J* = 11.0, 6.6 Hz). According to the ^13^C NMR and DEPT spectra (Table 2), there were twenty distinct carbon resonances, categorized into four methyl groups, six methylene groups, five methine groups, and five signals from nonprotonated carbons (one ketocarbonyl group at *δ*_C_ 201.0, two olefinic groups at *δ*_C_ 155.8 and 154.3, one oxygenated group at *δ*_C_ 79.8, and one sp^3^ quaternary carbon). The data mentioned above indicated that compound **1** is a prenylaromadendrane-type diterpene, and 1D NMR data analysis suggested that the signals of **1** were similar to those of 4*β*-hydroxy-15-(3-methyl-2-butenyl) aromadendr-*Δ*^10(12)^-en [9]. In the ^13^C NMR spectrum, a key difference was that the methylene singlet at *δ*_C_ 25.6 (C-16) was substituted by a ketone carbonyl signal at *δ*_C_ 201.0, along with a downfield shift in C-15 (*δ*_C_ 39.1→*δ*_C_ 56.6) and C-18 (*δ*_C_ 131.2→*δ*_C_ 155.8) carbon signals adjacent to C-16. Furthermore, the specific UV absorption wavelength (239 nm) combined with the molecular weight of **1** suggested that the methylene at C-16 was transformed into a ketone carbonyl, forming an *α*,*β*-unsaturated ketone carbonyl group with the double bond between C-17 and C-18. The conclusion also was further supported by the HMBC correlations (Figure 2) of H_2_-15/C-16 (*δ*_C_ 201.1), C-17 (*δ*_C_ 124.1), and H_3_-19/C-17, C-18 (*δ*_C_ 155.8). As a result, the planar structure of **1** was determined (Figure 1).

**Figure 3:**
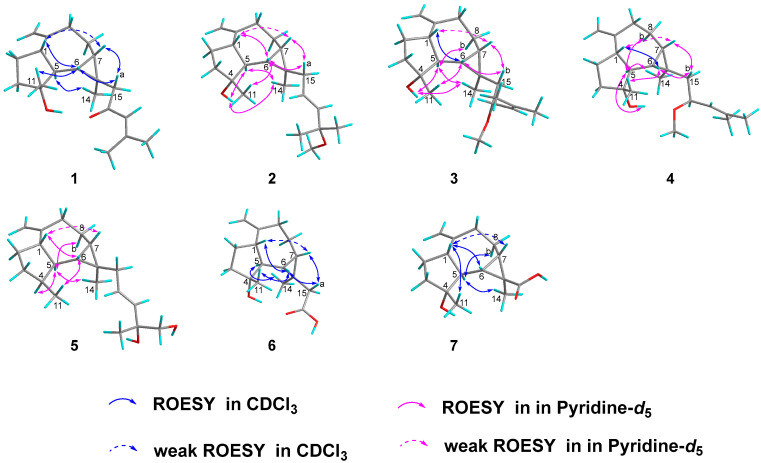
Key ROESY correlations of **1**–**7**.

**Figure 4:**
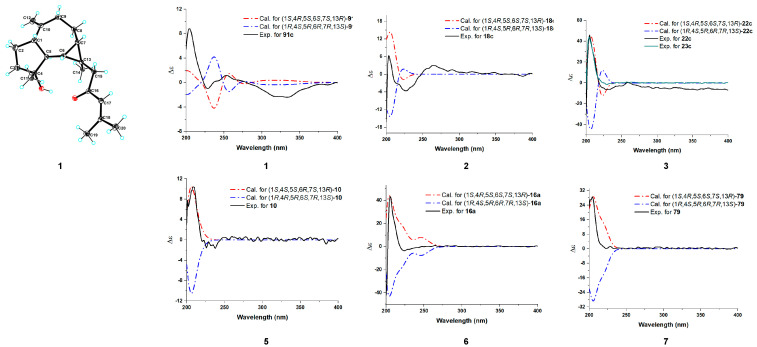
X-ray structures of **1**. Comparison of B3LYP/6-311g(d,p) calculated ECD spectra together with the experimental spectra of **1**–**7** in MeOH. **1**: ☌ = 0.3 eV; shift = 11 nm, scaling factor = 2.5; **2**: ☌ = 0.29 eV, shift = 10 nm, scaling factor = 0.9; **3/4**: ☌ = 0.29 eV; shift = 25 nm, scaling factor = 1.53; **5**: ☌ = 0.29 eV, shift = 10 nm, scaling factor = 1.67; **6**: ☌ = 0.35 eV, shift = 23 nm, scaling factor = 4.5; **7**: ☌ = 0.27 eV, shift = 0 nm, scaling factor = 26.5.

To establish the relative configuration of **1,** a ROESY experiment was conducted. The ROESY correlations (Figure 3) between H-7/Ha-15, H-1 (weak), H-6/Ha-15, H-1, H_3_-11, and H_3_-14/H-5 implied that H-6, H-7, H-1, CH_3_-11, and H_2_-15 were on the same face and *β*-oriented, while CH_3_-14 and H-5 were *α*-oriented. The absolute configuration of compound **1** was identified through electronic circular dichroism (ECD) calculations at the B3LYP/6-311g(d,p) level. The data demonstrated that the ECD spectrum calculated for (1*S*,4*R*,5*S*,6*S*,7*S*,13*R*)-**1** (Figure 4) were very similar to the experimental spectrum, confirming the absolute configuration of **1** as 1*S*,4*R*,5*S*,6*S*,7*S*,13*R*. Luckily, a suitable crystal of **1** was acquired, and X-ray diffraction analysis with CuK*α* radiation was performed (Figure 4), confirming the previous conclusion.

Euphraticanoid O (**2**) was isolated as a colorless gum, with a molecular formula of C_21_H_34_O_2_, determined from the HRESIMS ion peak at *m*/*z* 341.2452 [M + H]^+^ (calcd for C_21_H_35_O_2_, 341.2451). According to the ^1^H and ^13^C NMR data (Table 1 and Table 2), compound **2** resembled boscartol C [10], except for a methoxyl group resonating at *δ*_H_/*δ*_C_ 3.19/50.5 (CH_3_-21) in pyridine-*d*_5_ instead of the hydroxy group. This was corroborated by the HMBC correlations (Figure 2) of H_3_-21/C-18 (*δ*_C_ 75.2), H_3_-18/C-17, C-18, and H_3_-19/C-17, C-18. Additionally, compound **2** shared the same relative configurations at the chiral centers in C-1, C-4, C-5, C-6, C-7, and C-13 as **1**, based on similar findings in the 1D NMR (Table 1 and Table 2) and ROESY spectra (Figure 3). Moreover, the large coupling constant of H-17 (*J* = 15.7 Hz) indicated that the double bond between H-16 and H-17 is an *E* configuration. Finally, the absolute configuration of **2** as 1*S*,4*R*,5*S*,6*S*,7*S*,13*R* was validated through the comparison of the experimental and calculated ECD curves (Figure 4).

Euphraticanoid P (**3**) was identified with a molecular formula of C_21_H_34_O_2_, derived from the positive HRESIMS data. In the ^1^H and ^13^C NMR data (Table 1 and Table 2) for compound **3**, there were strong resemblances to compound **1**, with the exception of the missing ketone resonance at *δ*_C_ 201.0 (C-16). Instead, oxymethine signals were detected at *δ*_H_/*δ*_C_ 4.14/75.7 (CH-16) in CDCl_3_ in the 1D NMR spectra of **3**. Furthermore, more shielded resonances of protons for Ha-15 (*δ*_H_ 2.83→*δ*_H_ 1.52), Hb-15 (*δ*_H_ 1.95→*δ*_H_ 1.25), and H-17 (*δ*_H_ 6.00→*δ*_H_ 4.95), and carbons for C-15 (*δ*_C_ 56.6→*δ*_C_ 48.8) and C-18 (*δ*_C_ 155.8→*δ*_C_ 135.7) in CDCl_3_ were observed due to the conversion from a ketone carbonyl to an methoxyl group at C-16, as confirmed by the ^1^H-^1^H COSY correlations (Figure 2) of H_2_-15/H-16/H-17 and the HMBC correlations (Figure 2) of H_3_-21/C-16. Then, based on ROESY correlations and 1D NMR data similar to those of **1** and **2**, the relative configuration of 1S*,4R*,5S*,6S*,7S*,13R* in **3** was identified. The relative configuration of C-16 was not assigned because the flexible side chain at C-16 poses a significant challenge. Ultimately, the absolute configuration of compound **3** was identified as 1*S*,4*R*,5*S*,6*S*,7*S*,13*R* by comparing the experimental CD curve with the calculated ECD curve (Figure 4).

Euphraticanoid Q (**4**) was isolated as a colorless gum and had the same molecular formula as **3**, as determined by HRESIMS analysis. Detailed analysis of the 1D NMR spectra revealed that the ^13^C NMR data for **4** match those of **3**. However, the ^1^H NMR data showed slight differences, such as (A) variations in the chemical shift, primarily at H-6 (*δ*_H_ 0.79→*δ*_C_ 0.94), Ha-15 (*δ*_H_ 2.02→*δ*_C_ 1.63), Hb-15 (*δ*_H_ 1.27→*δ*_C_ 1.57), and H-17 (*δ*_H_ 5.14→*δ*_C_ 5.20), and (B) changes in the splitting pattern of oxymethine proton at *δ*_H_ 4.21 from *dt* (*J* = 9.4, 6.6) to *td* (*J* = 8.4, 6.6 Hz), indicating that **4** was the C-16-epimer of **3**. Furthermore, the conclusion was reinforced by identical ROESY correlations (Figure 3) involving H-6/H-1, Hb-15, H_3_-11, H-7/H-1 (weak), Hb-15, and H-5/Hb-8, H_3_-14, 4-OH, and the experimental ECD spectrum in **4** the same as **3**.

Euphraticanoid R (**5**) was isolated as a colorless gum with a molecular formula of C_20_H_32_O_2_, as revealed by HRESIMS. The primary characteristics of the 1D NMR spectra (Table 2 and Table 3) for compound **5** was similar to those for compound **2**, except that the hydroxy group signal at C-4 was absent and a hydroxymethyl group (*δ*_H_ 3.98, d, *J* = 10.4 Hz and 3.93, d, *J* = 10.4 Hz; *δ*_C_ 71.5) appeared at C-20 in the place of a methyl group. These modifications were verified by the change in splitting pattern of the methyl group at C-4 from *s* to *d* (*J* = 8.6 Hz) and the HMBC correlations (Figure 2) involving H_3_-11/C-3, C-4, C-5 and H_2_-20/C-17 (*δ*_C_ 138.7), C-18 (*δ*_C_ 73.6). The ROESY correlations of H-6/H-1, H_3_-11, H-1/H-7 (weak) and H-5/H-4, H_3_-14, Hb-8 were used to define its relative configuration as 1S*,4S*,5S*,6R*,7S*,13R*. In conclusion, the resemblance of the ECD curves of **5** to those of **1**–**4** (Figure 4) implied that the absolute configuration of **5** is 1*S*,4*S*,5*S*,6*R*,7*S*,13*R*.

Euphraticanoid S (**6**) was confirmed to be C_16_H_24_O_3_ using its HRESIMS and ^13^C NMR data. The 1D and 2D NMR data (Table 2 and Table 3) exhibited structural characteristics identical to those of spathulenol [9]. The primary distinction was the presence of an acetate moiety (*δ*_H_/*δ*_C_ 2.68, 1.87/46.2; *δ*_C_ 176.6) at C-13, instead of a methyl group in **6**, as indicated by the HMBC correlations (Figure 2) of H_3_-14/C-6, C-7, C-13, C-15, and H_2_-15/C-16 (*δ*_C_ 176.6). The ROESY correlations (Figure 3) involving H-6/H-1, Ha-15, H_3_-11, H-7/H-1 (weak), Ha-15, and H-5/H_3_-14 indicated that H-1, H-6, H-7, H_3_-11, and H_2_-15 were *β*-oriented, while H-5 and H_3_-14 were *α*-oriented. Finally, the absolute configuration of **6** was identified as 1*S*,4*R*,5*S*,6*S*,7*S*,13*R* by comparing the calculated ECD spectra with the experimental results (Figure 4).

Euphraticanoid T (**7**) was isolated as white solids, with a molecular formula of C_15_H_22_O_3_ determined through HRESIMS data. The signals in the ^1^H and ^13^C NMR spectra (Table 2 and Table 3) were almost identical to those of **6**, except for the substitution of the acetate moiety at C-13 with a carboxyl group, confirmed by the HMBC correlations (Figure 2) of H-7/C-13, C-14, C-15 (*δ*_C_ 177.4), H-6/C-13, C-14, C-15, and H_3_-14/C-15. Moreover, the ROESY correlations (Figure 3) of H-1/H_3_-14, H-6, H-7 (weak), and H-5/H_3_-14 were used to determine the *β*-orientation of the carboxyl group. The absolute configuration was determined to be 1*S*,4*R*,5*S*,6*S*,7*S*,13*R* by comparing the calculated and experimental ECD spectra.

### 2.2. Biological Activity

**Figure 5:**
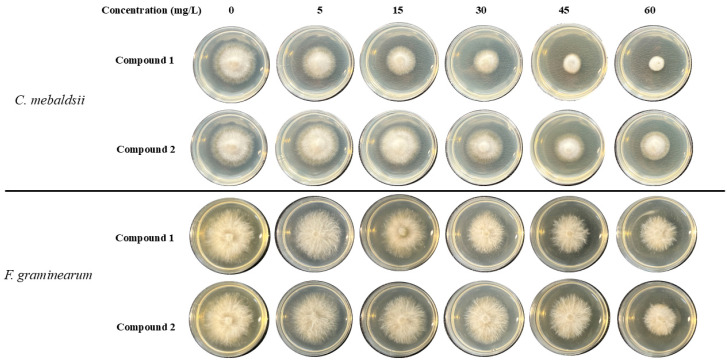
The fungicidal efficacy of compounds **1** and **2**.

The fungicidal activities of compounds against *Fusarium graminearum*, *Curvularia mebaldsii*, *Curvularia lunata*, *Botrytis cinerea*, *Alternaria altanata*, *Sclerotinia sclerotiorum*, and *Rhizoctonia solani* were evaluated, with the commercial fungicide hymexazol used as a positive control. For all compounds, preliminary screening was carried out at a concentration of 80 mg/L, and the results are shown in Table 4. It revealed that these compounds display board-spectrum fungicidal activities. In particular, compounds **1**–**5** show considerable fungicidal activities, which possess inhibitory rates (IRs) of over 50% towards most pathogenic fungi. Among them, compound **1** displays notable anti-*C. mebaldsii* with 80% IRs, whose activity far surpasses that of hymexazol. Given the sufficient quantity and attractive fungicidal activities against *F. graminearum* and *C. mebaldsii* of compounds **1** and **2** in preliminary screening, their maximal effect (EC_50_) values of 50% towards *F. graminearum* and *C. mebaldsii* were further measured (Figure 5 and Table 5). This demonstrated that compounds **1** and **2** possessed 15.7 and 42.1 mg/L EC_50_ values against *C. mebaldsii, respectively,* while the fungicidal levels were superior to that of hymexazol (84.8 mg/L). In addition, the EC_50_ values of compounds **1** (68.8 mg/L) and **2** (78.0 mg/L) against *F. graminearum* were close to that of hymexazol (66.3 mg/L). This finding suggested that compounds **1** and **2** could be potential alternative lead compounds for the design of fungicides.

## 3. Experimental Section

### 3.1. Fungal Material

The origin and verification of *P. euphratica* resins matched our previous study [6], and the voucher specimen (CHYX0573) was stored at Shenzhen University.

### 3.2. Extraction and Isolation

The dried resins (50.0 kg) were soaked in 95% ethanol (300 L × 3 × 24 h) to produce a crude extract, which was then mixed with water and separated using ethyl acetate. Subsequently, the EtOAc solution was concentrated under reduced pressure to produce a 12.0 kg EtOAc soluble extract. Subsequently, the extract underwent separation by multiple chromatography, obtaining compounds **1**–**7**. For additional detailed isolation procedures, consult the Supplementary Information.

### 3.3. Crystal Structure of ***1***

The crystallographic information for euphraticanoid N (**1**) (deposition number CCDC 2422553) is available at the Cambridge Crystallographic Data Centre. You can access the data for free at www.ccdc.cam.ac.uk/data_request/cif (accessed on 15 February 2025), or by emailing data request@ccdc.cam.ac.uk, or by contacting The Cambridge Crystallographic Data Centre, 12 Union Road, Cambridge CB2 1EZ, UK; fax: +44 1223 336033.

### 3.4. ECD Calculations for Compounds ***1***–***7***

The main conformers of compounds **1**–**7** were optimized using Gaussian 09 at the B3LYP/6-311g(d,p) level. Then, we used the same method for the optimized conformers for the ECD calculations. Solvent effects were incorporated using the PCM model, with methanol serving as the solvent. The percentages of each conformation can be found in Table S3. The ECD spectra were ultimately derived from the Boltzmann-calculated contribution of each conformer.

### 3.5. Fungicidal Activity Assay

The fungicidal activity of compounds **1**–**7** was evaluated against the fungal strains using the method according to the literature [11,12,13,14]. Briefly, pathogen mycelial plugs (diameter 0.4 cm) were adhered to the center of potato dextrose agar (PDA) plates containing specific concentrations of the compounds (with equal volumes of DMSO as a control). The plates were cultivated at 26 °C for three days, and the colony diameter was measured in triplicate to assess the growth rate. The inhibition rates were identified using the formula *I*% = [(*C* − *T*)/(*C* − 0.4)] × 100%. Here, *C* means the diameter of fungal growth treated by DMSO; *T* means the diameter of fungal growth treated by the compounds; and *I* means the inhibition rate. An average was taken, and the standard deviation was measured.

## 4. Conclusions

To conclude, the current study led to the identification of five prenylaromadendrane-type diterpenes (**1**–**5**) and two aromadendrane-type sesquiterpenes (**6** and **7**) from *P. euphratica* resins. In addition, the fungicidal activities of compounds **1**–**7** were evaluated. The bioassay results revealed that compound **1** displays potent fungicidal activities against *C. mebaldsii* and *F. graminearum* with the EC_50_ values of 15.7 and 68.6 mg/L, respectively. According to our knowledge, this is one of few studies on fungicidal agents derived from *P. euphratica*, providing an innovative structural model for fungicide design.

## Figures and Tables

**Table 1:** ^1^H NMR (600 MHz) data for **1**–**4** (*δ* in ppm, *J* in Hz).

No	1 ^a^	2 ^b^	3 ^b^	4 ^b^
1	2.28, m	2.27, overlap	2.26, overlap	2.26, overlap
2	Ha: 1.94, m	Ha: 2.27, overlap	Ha: 2.26, overlap	Ha: 2.26, overlap
	Hb: 1.68, overlap	Hb: 1.68, m	Hb: 1.68, m	Hb: 1.67, overlap
3	Ha: 1.84, m	Ha: 2.05, m	Ha: 2.04, m	Ha: 2.05, m
	Hb: 1.68, overlap	Hb: 1.67, m	Hb: 1.67, m	Hb: 1.67, overlap
5	1.43, t (11.0)	1.74, t (10.5)	1.72, t (10.4)	1.77, t (10.3)
6	0.60, t-like (11.0)	0.64, t-like (10.5)	0.61, t (10.4)	0.58, t (10.4)
7	0.77, td (11.0, 6.6)	0.77, td (10.5, 6.1)	0.79, td (10.4, 6.4)	0.94, td (10.4, 6.4)
8	Ha: 2.02, m	Ha: 1.97, m	Ha: 1.93, dt (13.0, 6.4)	Ha: 2.03, m
	Hb: 0.99, m	Hb: 1.13, m	Hb: 1.08, q-like (13.0)	Hb: 1.15, q-like (12.8)
9	Ha: 2.43, dd (13.0, 6.5)	Ha: 2.48, dd (13.2, 5.9)	Ha: 2.48, dd (13.0, 6.4)	Ha: 2.48, dd (12.8, 6.0)
	Hb: 1.98, m	Hb: 2.14, t (13.2)	Hb: 2.12, t (13.0)	Hb: 2.13, t (12.8)
11	1.24, s	1.54, s	1.51, s	1.54, s
12	Ha: 4.73, br s	Ha: 4.89, br s	Ha: 4.88, br s	Ha: 4.89, br s
	Hb: 4.69, br s	Hb: 4.80, br s	Hb: 4.81, br s	Hb: 4.81, br s
14	1.03, s	1.17, s	1.21, s	1.28, s
15	Ha: 2.83, d (17.9)	Ha: 2.06, m	Ha: 2.02, dd (13.7, 6.6)	Ha: 1.63, dd (14.3, 4.1)
	Hb: 1.95, d (17.9)	Hb: 2.03, m	Hb: 1.27, dd (13.7, 6.6)	Hb: 1.57, dd (14.3, 8.4)
16		5.77, td (15.7, 7.1)	4.21, dt (9.4, 6.6)	4.23, td (8.4, 4.1)
17	6.00, br s	5.66, d (15.7)	5.14, d (9.4)	5.20, br d (8.4)
19	1.87, d (0.8)	1.31, s	1.75, s	1.72, br s
20	2.14, d (0.8)	1.31, s	1.70, s	1.70, br s
21		3.19, s	3.27, s	3.28, s
4-OH		5.37, s	5.15, s	5.29, s

^a^ NMR spectra data were recorded in CDCl_3_. ^b^ NMR spectra data were recorded in pyridine-*d*_5._

**Table 2:** ^13^C NMR (150 MHz) data for **1**–**7** (δ in ppm).

No	1 ^a^	2 ^b^	3 ^b^	4 ^b^	5 ^b^	6 ^a^	7 ^a^
1	49.0, CH	54.3, CH	53.4, CH	54.0, CH	54.0, CH	49.1, CH	52.2, CH
2	24.2, CH_2_	27.6, CH_2_	27.2, CH_2_	27.5, CH_2_	29.8, CH_2_	24.3, CH_2_	26.8, CH_2_
3	39.5, CH_2_	43.0, CH_2_	42.5, CH_2_	42.9, CH_2_	35.6, CH_2_	39.5, CH_2_	41.2, CH_2_
4	79.8, C	80.3, C	80.2, C	80.2, C	36.2, CH	80.5, C	81.2, C
5	53.5, CH	54.3, CH	54.3, CH	54.3, CH	44.1, CH	53.3, CH	52.9, CH
6	24.7, CH	29.9, CH	29.9, CH	30.0, CH	28.3, CH	24.9, CH	32.4, CH
7	26.5, CH	26.8, CH	27.3, CH	27.8, CH	26.6, CH	26.9, CH	31.2, CH
8	25.1, CH_2_	25.5, CH_2_	25.6, CH_2_	25.5, CH_2_	25.4, CH_2_	25.2, CH_2_	24.6, CH_2_
9	39.1, CH_2_	39.6, CH_2_	39.7, CH_2_	39.7, CH_2_	39.6, CH_2_	38.9, CH_2_	38.4, CH_2_
10	154.3, C	154.4, C	154.7, C	154.6, C	154.7, C	154.0, C	152.3, C
11	23.9, CH_3_	27.3, CH_3_	26.6, CH_3_	26.9, CH_3_	18.0, CH_3_	23.9, CH_3_	25.9, CH_3_
12	106.7, CH_2_	106.7, CH_2_	106.7, CH_2_	106.6, CH_2_	106.4, CH_2_	107.0, CH_2_	107.7, CH_2_
13	20.5, C	24.9, C	22.7, C	23.2, C	24.7, C	21.0, C	30.3, C
14	14.5, CH_3_	14.6, CH_3_	14.9, CH_3_	15.3, CH_3_	14.0, CH_3_	14.3, CH_3_	23.5, CH_3_
15	56.6, CH_2_	46.4, CH_2_	49.6, CH_2_	49.8, CH_2_	46.3, CH_2_	46.2, CH_2_	177.4, C
16	201.0, C	128.6, CH	76.6, CH	76.9, CH	126.7, CH	176.6, C	
17	124.1, CH	137.8, CH	127.9, CH	128.2, CH	138.7, CH		
18	155.8, C	75.2, C	135.3, C	134.9, C	73.6, C		
19	27.9, CH_3_	26.9, CH_3_	26.2, CH_3_	26.2, CH_3_	25.9, CH_3_		
20	21.0, CH_3_	26.2, CH_3_	18.6, CH_3_	18.6, CH_3_	71.5, CH_3_		
21		50.5, CH_3_	55.6, CH_3_	55.8, CH_3_			

^a^ NMR spectra data were recorded in CDCl_3_. ^b^ NMR spectra data were recorded in pyridine-*d*_5_.

**Table 3:** ^1^H NMR (600 MHz) data of **5**–**7** (δ in ppm, J in Hz).

No	5 ^b^	6 ^a^	7 ^a^
1	2.17, m	2.30, m	2.18, td (10.7, 6.6)
2	Ha: 1.66, m	Ha: 1.95, m	Ha: 1.92, m
	Hb: 1.57, m	Hb: 1.67, m	Hb: 1.67, m
3	Ha: 1.79, ddd (12.6, 6.4, 3.2)	Ha: 1.81, m	Ha: 1.86, m
	Hb: 1.16, m	Hb: 1.68, m	Hb: 1.57, ddd (12.7, 10.4, 6.5)
4	2.03, m		
5	1.39, q (10.4)	1.41, t (10.8),	1.95, t (10.7)
6	0.65, t (10.4)	0.73, t (10.8)	0.98, t-like (10.7)
7	0.74, td (10.4, 6.6)	0.88, td (10.8, 6.8)	1.20, td (10.7, 6.1)
8	Ha: 1.90, dt (12.5, 6.1)	Ha: 2.05, dt (14.6, 6.8)	Ha: 2.03, m
	Hb: 1.04, m	Hb: 0.98, m	Hb: 1.45, q-like (12.3)
9	Ha: 2.42, dd (13.1, 6.1)	Ha: 2.43, dd (13.2, 6.8)	Ha: 2.44, dd (13.6, 6.4)
	Hb: 2.05, m	Hb: 1.99, m	Hb: 2.01, m
11	1.01, d (8.6)	1.29, s	1.35, s
12	4.76, s	Ha: 4.74, br s	Ha: 4.72, br s
		Hb: 4.71, br s	Hb: 4.69, br s
14	1.00, s	1.08, s	1.35, s
15	2.06, m	Ha: 2.68, d (16.8)	
		Hb: 1.87, d (16.8)	
16	6.18, dt (15.0, 7.1)		
17	6.03, d (15.0)		
19	1.67, (s)		
20	3.98, d (10.4)		
	3.93, d (10.4)		

^a^ NMR spectra data were recorded in CDCl_3_. ^b^ NMR spectra data were recorded in pyridine-*d*_5_.

**Table 4:** Fungicidal activities of compounds **1**–**7** at 80 mg/L.

Cpd.	FG *	CM	CL	BC	AA	SS	RS
**1**	61.4 ± 4.3	80.0 ± 5.3	54.5 ± 0.0	36.2 ± 7.9	53.3 ± 2.9	61.4 ± 6.1	56.0 ± 9.2
**2**	55.7 ± 6.5	60.0 ± 2.7	51.5 ± 2.6	55.2 ± 6.0	53.3 ± 2.9	36.8 ± 13.9	52.0 ± 0.0
**3**	65.7 ± 0.0	NT **	47.0 ± 2.6	56.9 ± 6.0	51.7 ± 2.9	66.7 ± 8.0	38.0 ± 3.5
**4**	58.6 ± 2.5	NT	NT	34.5 ± 7.9	51.7 ± 5.8	49.1 ± 13.2	40.0 ± 0.0
**5**	54.3 ± 6.5	72.3 ± 0.0	NT	56.9 ± 3.0	63.3 ± 2.9	40.4 ± 8.0	40.0 ± 6.0
**6**	37.1 ± 4.9	NT	NT	5.2 ± 3.0	13.3 ± 2.9	36.8 ± 0.0	20.0 ± 3.5
**7**	NT	NT	NT	34.5 ± 6.0	NT	33.3 ± 3.0	NT
hymexazol	61.4 ± 0.0	50.8 ± 2.7	83.3 ± 2.6	70.7 ± 3.0	66.7 ± 2.9	61.4 ± 3.0	62.0 ± 3.5

* FG: Fusarium graminearum; CM: Curvularia mebaldsii; CL: Curvularia lunata; BC: Botrytis cinerea; AA: Alternaria altanata; SS: Sclerotinia sclerotiorum; RS: Rhizoctonia solani. ** NT means no test.

**Table 5:** The EC_50_ values of compounds **1** and **2**.

	Compound	R2	Regression Equation (y = ax + b)	EC_50_ (mg/L)
*C. mebaldsii*	**1**	0.943	y = 1.171x + 3.601	15.7
	**2**	0.950	y = 0.712x + 3.844	42.1
	hymexazol	0.953	y = 2.314x + 0.537	84.8
*F. graminearum*	**1**	0.961	y = 1.426x + 2.381	68.6
	**2**	0.983	y = 1.369x + 2.410	78.0
	hymexazol	0.961	y = 1.561x + 2.157	66.3

## Data Availability

All the data in this research are presented in manuscript and Supplementary Materials.

## Supplementary Materials

The following supporting information can be downloaded at https://www.mdpi.com/article/10.3390/ijms26052187/s1: NMR data for ^1^H and ^13^C in CDCl_3_ of **2**–**4**, along with general procedures, detailed isolation procedures, characterization data, and NMR, HRESIMS, UV, and CD spectra for compounds **1**–**7**, as well as X-ray crystallography data for compound **1**. ECD calculations for compounds **1**–**7** can also be found here. Reference [15] are cited in the Supplementary Materials.
