# Supplementary material for: Euphraticanoids N–T: Aromadendrane-Type Diterpenes and Sesquiterpenes with Fungicidal Activities from Populus euphratica Resins
Source: Int J Mol Sci. 2025 Feb 28;26(5):2187. doi: 10.3390/ijms26052187 (PMC11900364; doi:10.3390/ijms26052187)
Supplement: Supplementary file 1 [file ijms-26-02187-s001.zip › ijms-3506934-supplementary.pdf]

Supplementary Information

Euphraticanoids N–T: Aromadendrane-type Diterpenes and Sesquiterpenes with Fungicidal Activities from *Populus euphratica* Resins

Qin-Bin Jiang [1,2,†], Yun-Yun Liu [2,†], Danling Huang [2], and Yong-Xian Cheng [1,2*]

1 School of Pharmacy, Guangdong Pharmaceutical University, Guangzhou, 510006，China; jqb0925@163.com (Q.-B.J.); yxcheng@szu.edu.cn (Y.-X.C.);

2 Guangdong Provincial Key Laboratory of Chinese Medicine Ingredients and Gut Microbiomics, Institute for Inheritance-Based Innovation of Chinese Medicine, School of Pharmacy, Shenzhen University, Shenzhen 518055, Guangdong, PR China; liuyy@szu.edu.cn (Y.-Y.L.); leonchemistry@szu.edu.cn (D.-L.H.);

* Correspondence: yxcheng@szu.edu.cn; Tel.: +86-0755-26902073;

† These authors contributed equally to this work.

E-mail address: yxcheng@szu.edu.cn.

Content
**^{1}H and ^{13}C NMR data in $CDCl_3$ of the new compounds 1–7**
**Table S1** ^{1}H NMR (600 MHz) data in $CDCl_3$ of **2–4** (δ in ppm, J in Hz)
**Table S2** ^{13}C NMR (150 MHz) data in $CDCl_3$ of **2–4** (δ in ppm)
General Procedures
Detailed isolation procedures
Compound Characterization Data
**Spectra of the new compounds 1–7**
**Figure S1.** ^{1}H NMR spectrum of **1** in $CDCl_3$.
**Figure S2.** ^{13}C NMR and DEPT spectra of **1** in $CDCl_3$.
**Figure S3.** ^{1}H-^{1}H COSY spectrum of **1** in $CDCl_3$.
**Figure S4.** HSQC spectrum of **1** in $CDCl_3$.
**Figure S5.** HMBC spectrum of **1** in $CDCl_3$.
**Figure S6.** ROESY spectrum of **1** in $CDCl_3$.
**Figure S7.** HRESIMS of **1**.
**Figure S8.** CD and UV spectra of **1**.
**Figure S9.** ^{1}H NMR spectrum of **2** in Pyridine-d_5.
**Figure S10.** ^{13}C NMR and DEPT spectra of **2** in Pyridine-d_5.
**Figure S11.** ^{1}H-^{1}H COSY spectrum of **2** in Pyridine-d_5.
**Figure S12.** HSQC spectrum of **2** in Pyridine-d_5.
**Figure S13.** HMBC spectrum of **2** in Pyridine-d_5.
**Figure S14.** ROESY spectrum of **2** in Pyridine-d_5.
**Figure S15.** ^{1}H NMR spectrum of **2** in $CDCl_3$.
**Figure S16.** ^{13}C NMR and DEPT spectra of **2** in $CDCl_3$.
**Figure S17.** ^{1}H-^{1}H COSY spectrum of **2** in $CDCl_3$.
**Figure S18.** HSQC spectrum of **2** in $CDCl_3$.
**Figure S19.** HMBC spectrum of **2** in $CDCl_3$.
**Figure S20.** ROESY spectrum of **2** in $CDCl_3$
**Figure S21.** HREIMS of **2**.
**Figure S22.** CD and UV spectra of **2**.
**Figure S23.** ^{1}H NMR spectrum of **3** in Pyridine-d_5.
**Figure S24.** ^{13}C NMR and DEPT spectra of **3** in Pyridine-d_5.
**Figure S25.** ^{1}H-^{1}H COSY spectrum of **3** in Pyridine-d_5.
**Figure S26.** HSQC spectrum of **3** in Pyridine-d_5.
**Figure S27.** HMBC spectrum of **3** in Pyridine-d_5.
**Figure S28.** ROESY spectrum of **3** in Pyridine-d_5.
**Figure S29.** ^{1}H NMR spectrum of **3** in $CDCl_3$.
**Figure S30.** ^{13}C NMR and DEPT spectra of **3** in $CDCl_3$.
**Figure S31.** ^{1}H-^{1}H COSY spectrum of **3** in $CDCl_3$.
**Figure S32.** HSQC spectrum of **3** in $CDCl_3$.

**Figure S33.** HMBC spectrum of **3** in $CDCl_3$.
**Figure S34.** ROESY spectrum of **3** in $CDCl_3$.
**Figure S35.** HREIMS of **3**.
**Figure S36.** CD and UV spectra of **3**.
**Figure S37.** ^{1}H NMR spectrum of **4** in Pyridine-d_5.
**Figure S38.** ^{13}C NMR and DEPT spectra of **4** in Pyridine-d_5.
**Figure S39.** ^{1}H-^{1}H COSY spectrum of **4** in Pyridine-d_5.
**Figure S40.** HSQC spectrum of **4** in Pyridine-d_5.
**Figure S41.** HMBC spectrum of **4** in Pyridine-d_5.
**Figure S42.** ROESY spectrum of **4** in Pyridine-d_5.
**Figure S43.** ^{1}H NMR spectrum of **4** in $CDCl_3$.
**Figure S44.** ^{13}C NMR and DEPT spectra of **4** in $CDCl_3$.
**Figure S45.** ^{1}H-^{1}H COSY spectrum of **4** in $CDCl_3$.
**Figure S46.** HSQC spectrum of **4** in $CDCl_3$.
**Figure S47.** HMBC spectrum of **4** in $CDCl_3$.
**Figure S48.** ROESY spectrum of **4** in $CDCl_3$.
**Figure S49.** HREIMS of **4**.
**Figure S50.** CD and UV spectra of **4**.
**Figure S51.** ^{1}H NMR spectrum of **5** in Pyridine-d_5.
**Figure S52.** ^{13}C NMR and DEPT spectra of **5** in Pyridine-d_5.
**Figure S53.** ^{1}H-^{1}H COSY spectrum of **5** in Pyridine-d_5.
**Figure S54.** HSQC spectrum of **5** in Pyridine-d_5.
**Figure S55.** HMBC spectrum of **5** in Pyridine-d_5
**Figure S56.** ROESY spectrum of **5** in Pyridine-d_5.
**Figure S57.** HREIMS of **5**.
**Figure S58.** CD and UV spectra of **5**.
**Figure S59.** ^{1}H NMR spectrum of **6** in $CDCl_3$.
**Figure S60.** ^{13}C NMR and DEPT spectra of **6** in $CDCl_3$.
**Figure S61.** ^{1}H-^{1}H COSY spectrum of **6** in $CDCl_3$.
**Figure S62.** HSQC spectrum of **6** in $CDCl_3$.
**Figure S63.** HMBC spectrum of **6** in $CDCl_3$.
**Figure S64.** HMBC spectrum of **6** in $CDCl_3$.
**Figure S65.** HREIMS of **6**.
**Figure S66.** CD and UV spectra of **6**.
**Figure S67.** ^{1}H NMR spectrum of **7** in $CDCl_3$.
**Figure S68.** ^{13}C NMR and DEPT spectra of **7** in $CDCl_3$.
**Figure S69.** ^{1}H-^{1}H COSY spectrum of **7** in $CDCl_3$.
**Figure S70.** HSQC spectrum of **7** in $CDCl_3$.
**Figure S71.** HMBC spectrum of **7** in $CDCl_3$.
**Figure S72.** HMBC spectrum of **7** in $CDCl_3$.
**Figure S73.** HREIMS of **7**.
**Figure S74.** CD and UV spectra of **7**.
**X-ray crystallographic data of 1**

**ECD calculation for compounds 1–7**
**Selected conformation of 1 and their percentage**
**Figure S75.** Model compound of **1**.
**Figure S76.** The lowest energy conformers of **1** (the relative populations are in parentheses).
**Selected conformation of 2 and their percentage**
**Figure S77.** Model compound of **2**.
**Figure S78.** The lowest energy conformers of **2** (the relative populations are in parentheses).
**Selected conformation of 3 and 4 and their percentage**
**Figure S79.** Model compounds of **3** and **4**.
**Figure S80.** The lowest energy conformers of **3/4** (the relative populations are in parentheses).
**Selected conformation of 5 and their percentage**
**Figure S81.** Model compound of **5**.
**Figure S82.** The lowest energy conformers of **5** (the relative populations are in parentheses).
**Selected conformation of 6 and their percentage**
**Figure S83.** Model compound of **6**.
**Figure S84.** The lowest energy conformers of **6** (the relative populations are in parentheses).
**Figure S85.** Model compound of **7**.
**Figure S86.** The lowest energy conformers of **7** (the relative populations are in parentheses).
**Table S3.** Extracted heats and weighting factors of the optimized conformers of **1**–**7** at B3LYP/6-311g(d,p) level
**Table S4.** The Cartesian coordinates of the lowest energy conformers for **1–7**

Table S1 ^{1}H (600 MHz) data in $CDCl_3$ of **2–4** (*δ* in ppm, *J* in Hz)

No	**2**	**3**	**4**
1	2.22, td (10.2, 6.4)	2.24, td (10.6, 8.3)	2.25, td (9.2, 8.4)
2	Ha: 1.91, m	Ha: .1.95, overlap	Ha: 1.92, m
	Hb: 1.63, m	Hb: 1.65, m	Hb: 1.63, m
3	Ha: 1.76, m	Ha: 1.76, m	Ha: 1.77, m
	Hb: 1.59, m	Hb: 1.64, m	Hb: 1.62, m
5	1.33, t (10.4)	1.33, t (10.6)	1.35, overlap
6	0.57, t-like (10.2)	0.61, t-like (10.6)	0.54, t-like (10.4)
7	0.77, td (10.2, 6.1)	0.69, td (10.6, 6.5)	0.82, td (10.4, 6.2)
8	Ha: 1.98, m	Ha: 1.95, m	Ha: 1.99, m
	Hb: 1.04, m	Hb: 0.97, q-like (13.2)	Hb: 1.00, q-like (12.5)
9	Ha: 2.43, dd (13.3, 6.1)	Ha: 2.41, dd (13.2, 6.3)	Ha: 2.42, dd (12.5, 6.2)
	Hb: 2.05, t (13.3)	Hb: 1.99, t (13.2)	Hb: 2.02, t (12.5)
11	1.30, s	1.27, s	1.29, s
12	Ha: 4.70, br s	Ha: 4.71, br s	Ha: 4.70, br s
	Hb: 4.67, br s	Hb: 4.68, br s	Hb: 4.67, br s
14	1.02, s	1.03, s	1.05, s
15	1.97, m	Ha: 1.52, dd (14.0, 5.1)	Ha: 1.57, dd (14.3, 7.3)
		Hb: 1.25, dd (14.0, 9.1)	Hb: 1.35, overlap
16	5.59, td (7.0, 15.4)	4.14, td (9.1, 5.1)	4.05, ddd (9.1, 7.3, 5.1)
17	5.42, d (15.4)	4.95, br d (9.1)	5.05, br d (9.1)
19	1.25, s	1.75, br s	1.76, br s
20	1.25, s	1.69, br s	1.70, br s
21	3.15, s	3.21, s	3.21, s

Table S2 ^{13}C NMR (150 MHz) data in $CDCl_3$ of **2**–**4** (δ in ppm)

No	**2**	**3**	**4**
1	53.2, CH	49.8, CH	51.7, CH
2	26.5, CH_2	24.8, CH_2	25.7, CH_2
3	41.8, CH_2	39.6, CH_2	40.9, CH_2
4	81.2, C	80.3, C	80.8, C
5	54.0, CH	53.7, CH	53.8, CH
6	28.7, CH	26.8, CH	27.6, CH
7	26.4, CH	26.0, CH	27.4, CH
8	24.9, CH_2	25.2, CH_2	24.9, CH_2
9	38.9, CH_2	39.1, CH_2	39.0, CH_2
10	153.4, C	154.4, C	153.9, C
11	26.3, CH_3	24.3, CH_3	25.2, CH_3
12	106.5, CH_2	106.6, CH_2	106.5, CH_2
13	24.4, C	21.2, C	22.2, C
14	14.2, CH_3	13.6, CH_3	14.9, CH_3
15	45.8, CH_2	48.8, CH_2	48.7, CH_2
16	128.0, CH	75.7, CH	76.3, CH
17	137.1, CH	126.4, CH	127.0, CH
18	75.0, CH_3	135.7, CH_3	135.8, CH_3
19	26.3, CH_3	25.9, CH_3	26.1, CH_3
20	25.7, CH_3	18.4, CH_3	18.4, CH_3
21	50.4, CH_3	55.3, CH_3	55.6, CH_3

General Procedures

UV and CD spectra were recorded on a Chirascan instrument (Agilent Technologies, Santa Clara, CA, USA). Optical rotations were measured by an Anton Paar MCP-100 digital polarimeter. 1D NMR and 2D NMR spectra were obtained on a Bruker Avance 500 MHz or a Bruker Avance III 600 MHz spectrometer (Bruker, Karlsruhe, Germany), with TMS (Tetramethyl silane) as an internal standard. A Shimazu LC-20 CE AB SCIEX triple TOF X500R MS spectrometer (Shimadzu Corporation, Tokyo, Japan) was used to collect the HRESIMS data of **1**–**7**. Column chromatography was carried out using C-18 silica gel (40–60 μm; Daiso Co., Japan), MCI gel CHP 20P (75–150 μm, Mitsubishi Chemical Industries, Tokyo, Japan), and Sephadex LH-20 (Amersham Pharmacia, Uppsala, Sweden). Semi-preparative HPLC was conducted using an Agilent 1200 liquid chromatograph (Agilent Technologies, Santa Clara, CA, USA) with the YMC-Pack ODS-A (250 mm × 10 mm, i.d., 5 μm) column.

Detailed isolation procedures

The extract underwent separation into eight fractions (Fr.A–Fr.H) using a silica gel column with petroleum ether–acetone (50:1–1:1) as solvents system. Fr.B (980.0 g) was fractionated by an MCI gel CHP 20P column ($MeOH/H_2O$, 40%–100%) into twelve parts (Fr.B.1–Fr.B.12). Fr.B.7 (27.0 g) was filtrated over middle pressure liquid chromatography (MPLC) ($MeOH/H_2O$, 45%–100%) to give seven portions (Fr.B.7.1–Fr.B.7.7). Fr.B.7.3 (2.6 g) was separated via Sephadex LH-20 (MeOH) to afford three subfractions (Fr.B.7.3.1–Fr.B.7.3.3) and Fr.B.7.3.1 (2.3 g) was further divided into eight parts (Fr.B.7.3.1.1–Fr.B.7.3.1.8) by using a silica gel column eluted with petroleum ether–acetone (100:1–20:1). Among them, Fr.B.7.3.1.3 (499.1 mg) was submitted to preparative HPLC with aqueous MeCN (50–60%) to afford three parts (Fr.B.7.3.1.3.1–Fr.B.7.3.1.3.3) and Fr.B.7.3.1.3.1 (79.2 mg) was further purified by semi-preparative HPLC eluted with aqueous MeOH (80%; flow rate: 3 mL/min) to yield **1** (17.1 mg, t_R = 22.7 min). Fr.B.7.5 (8.2 g) was separated using a silica gel column eluted with petroleum ether–acetone (20:1–1:1) to yield ten parts (Fr.B.7.5.1–Fr.B.7.5.10) and Fr.B.7.5.8 (372.2 mg) was separated via Sephadex LH-20 (MeOH) to afford three portions (Fr.B.7.5.8.1–Fr.B.7.5.8.3). Fr.B.7.5.8.1 (104.2 mg) and Fr.B.7.5.8.2 (196.2 mg) were subjected to preparative TLC (petroleum ether–acetone, 5:1) to respectively give seven fractions Fr.B.7.5.8.1.1–Fr.B.7.5.8.1.7 and Fr.B.7.5.8.2.1–Fr.B.7.5.8.2.7. And then, Fr.B.7.5.8.1.6 and Fr.B.7.5.8.2.6 were merged to Fr.B.7.5.8.c.a. Fr.B.7.5.8.c.a (30.0 mg) was divided into two parts (Fr.B.7.5.8.c.a.1 and Fr.B.7.5.8.c.a.2) by using a silica gel column eluted with petroleum ether–EtOAc (2:1) and Fr.B.7.5.8.c.a.2 (17.2 mg) was purified by semi-preparative HPLC (aqueous MeCN, 90%; flow rate: 3 mL/min) to yield **5** (5.3 mg, t_R = 12.1 min).

Fr.E (370.0 g) was separated by an MCI gel CHP 20P column ($MeOH/H_2O$, 40%–100%) to afford seven parts (Fr.E.1–Fr.E.7). Fr.E.2 (5.7 g) was separated via Sephadex LH-20 (MeOH) to afford three portions (Fr.E.2.1–Fr.E.2.3). Fr.E.2.2 (1.2 g) was fractionated via vacuum liquid chromatography on silica gel washed with CH_2Cl_2–acetone (100:1–1:1) to yield six portions (Fr.E.2.2.1–Fr.E.2.2.6). Purification of Fr.E.2.2.2 (200.0 mg) was further performed with preparative HPLC with aqueous MeOH (38–95%) to afford Fr.E.2.2.2.1–Fr.E.2.2.2.9. And then, Fr.E.2.2.2.6 (4.5 mg) was further purified by semi-preparative HPLC with aqueous MeCN (24%; flow rate: 3 mL/min) to afford **7** (1.7 mg, t_R = 25.8 min). Fr.E.3 (57.2 g) was subjected to a RP-18 column eluted with gradient aqueous MeOH (50%–100%) to yield thirteen fractions (Fr.E.3.1–Fr.E.3.13). Fr.E.3.3 (38.0 g) was chromatographed on MPLC eluted with gradient

aqueous MeOH (45%–55%) to give eight fractions (Fr.E.3.3.1–Fr.E.3.3.8). Fr.E.3.3.3 (5.5 g) was further fractionated by using a RP-18 column eluted with aqueous MeOH (45%) to give two portions Fr.E.3.3.3.1 and Fr.E.3.3.3.2. Fr.E.3.3.3.2 (3.3 g) was further separated via vacuum liquid chromatography on silica gel washed with CH_2Cl_2–acetone (100:1–2:1) to afford five subfractions (Fr.E.3.3.3.2.1–Fr.E.3.3.3.2.5). Fr.E.3.3.3.2.3 (326.0 mg) was further purified by preparative HPLC with aqueous MeOH (40–100%) to afford three parts (Fr.E.3.3.3.2.3.1–Fr.E.3.3.3.2.3.3) and Fr.E.3.3.3.2.3.3.2 (57.8 mg) was further purified by semi-preparative HPLC (aqueous MeOH, 55%; flow rate: 3 mL/min) to afford **6** (4.71 mg, t_R = 16.2 min). Fr.E.3.3.6 (3.0 g) was separated via vacuum liquid chromatography on silica gel washed with petroleum ether–acetone (5:1–0:1) to give eight portions (Fr.E.3.3.6.1–Fr.E.3.3.6.8). Fr.E.3.3.6.2 (99.8 mg) was subjected to preparative TLC (petroleum ether–EtOAc, 12:1) to obtain five fractions Fr.E.3.3.6.2.1–Fr.E.3.3.6.2.5. Fr.E.3.3.6.2.2 (32.3 mg) was fractionated by using Sephadex LH-20 (MeOH) and further purified by semi-preparative HPLC (aqueous MeCN, 72%; flow rate: 3 mL/min) to afford **4** (4.63 mg, t_R = 26.7 min) and **3** (4.38 mg, t_R = 28.7 min). Fr.E.3.6.3 (91.0 mg) was purified by semi-preparative HPLC (aqueous MeOH, 78%; flow rate: 3 mL/min) to afford **2** (38.0 mg, t_R = 33.4 min).

Compound Characterization Data

Euphraticanoid N (**1**): colorless crystals; UV (MeOH) λ_{max} (logε) 239 (4.37), 200 (4.17) nm; {$[\alpha]_D^{20}$ –29.1 (*c* 1.1, MeOH); CD (MeOH) $\Delta\varepsilon_{333}$ –1.15, $\Delta\varepsilon_{253}$ +0.57, $\Delta\varepsilon_{228}$ –0.47, $\Delta\varepsilon_{204}$ +4.24}; HRESIMS *m/z* 303.2311 $[M+H]^+$ (calcd for $C_{20}H_{31}O_2$, 303.2319). ^{1}H and ^{13}C NMR data see Tables 1 and 3.

Euphraticanoid O (**2**): colorless gums; UV (MeOH) λ_{max} (logε) 232 (3.79), 200 (4.23) nm; {$[\alpha]_D^{20}$ –10.8 (*c* 0.37, MeOH); CD (MeOH) $\Delta\varepsilon_{256}$ +1.11, $\Delta\varepsilon_{227}$ –2.08, $\Delta\varepsilon_{203}$ +2.32}; HRESIMS *m/z* 341.2452 $[M+H]^+$ (calcd for $C_{21}H_{35}O_2$, 341.2451); ^{1}H and ^{13}C NMR data see Tables 1 and 3.

Euphraticanoid P (**3**): colorless gums; UV (MeOH) λ_{max} (logε) 200 (4.40) nm; {$[\alpha]_D^{20}$ –14.6 (*c* 0.41, MeOH); CD (MeOH) $\Delta\varepsilon_{204}$ +14.52}; HRESIMS *m/z* 341.2456 $[M+H]^+$ (calcd for $C_{21}H_{34}O_2Na$, 341.2451); ^{1}H and ^{13}C NMR data see Tables 1 and 3.

Euphraticanoid Q (**4**): colorless gums; UV (MeOH) λ_{max} (logε) 200 (4.41) nm; {$[\alpha]_D^{20}$ –5.8 (*c* 0.51, MeOH); CD (MeOH) $\Delta\varepsilon_{205}$ +21.04}; HRESIMS *m/z* 341.2464 $[M+H]^+$ (calcd for $C_{21}H_{34}O_2Na$, 341.2451); ^{1}H and ^{13}C NMR data see Tables 1 and 3.

Euphraticanoid R (**5**): colorless gums; UV (MeOH) λ_{max} (logε) 249 (3.63), 200 (4.33) nm, {$[\alpha]_D^{20}$ –5.3 (*c* 0.56, MeOH); CD (MeOH) $\Delta\varepsilon_{209}$ +3.55}; HRESIMS *m/z* 303.2321 $[M–H]^-$ (calcd for $C_{20}H_{31}O_2$, 303.2330); ^{1}H and ^{13}C NMR data see Tables 2 and 3.

Euphraticanoid S (**6**): white solids; UV (MeOH) λ_{max} (logε) 200 (4.14) nm; {$[\alpha]_D^{20}$ –8.0 (*c* 0.5, MeOH); CD (MeOH) $\Delta\varepsilon_{205}$ +1.94}; HRESIMS *m/z* 287.1621 $[M+H]^+$ (calcd for $C_{16}H_{24}O_3Na$, 287.1618); ^1H and ^{13}C NMR data see Tables 2 and 3.

Euphraticanoid T (**7**): white solids; UV (MeOH) λ_{max} (logε) 200 (4.14) nm; {$[\alpha]_D^{20}$ +15.0 (*c* 0.2, MeOH); CD (MeOH) $\Delta\varepsilon_{204}$ +7.73}; HRESIMS *m/z* 249.1490 $[M–H]^-$ (calcd for $C_{15}H_{21}O_3$, 249.1496); ^1H and ^{13}C NMR data see Tables 2 and 3.

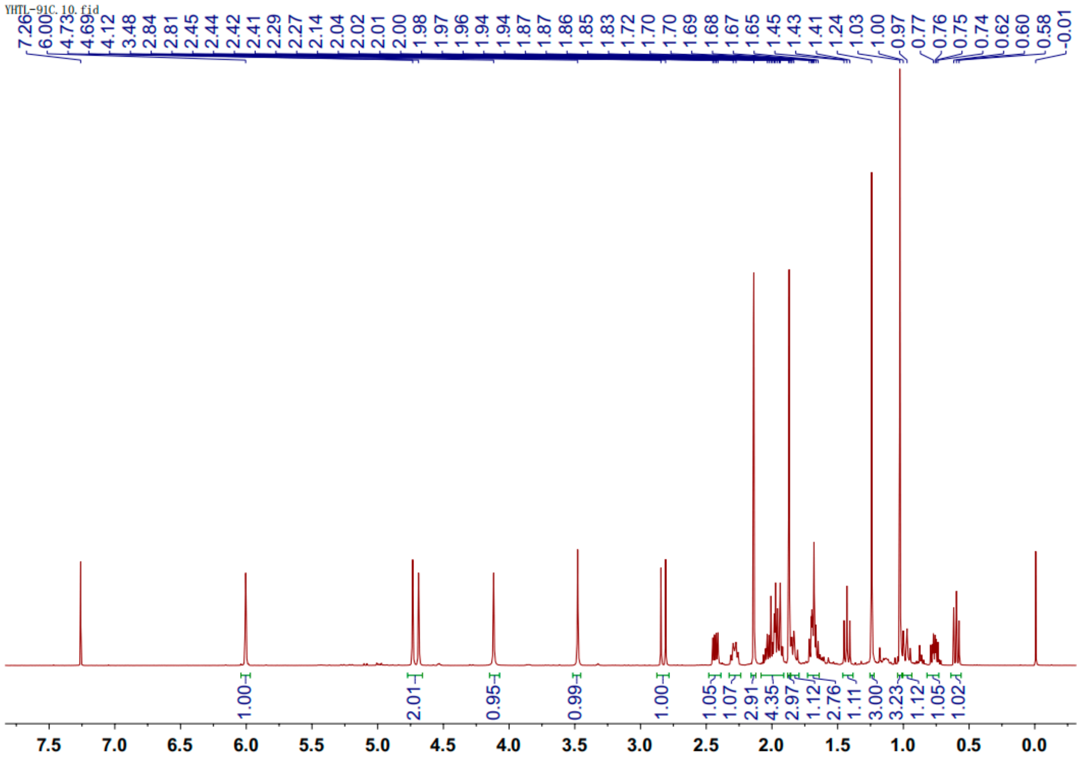

Figure S1. ^1H NMR spectrum of **1** in $CDCl_3$.

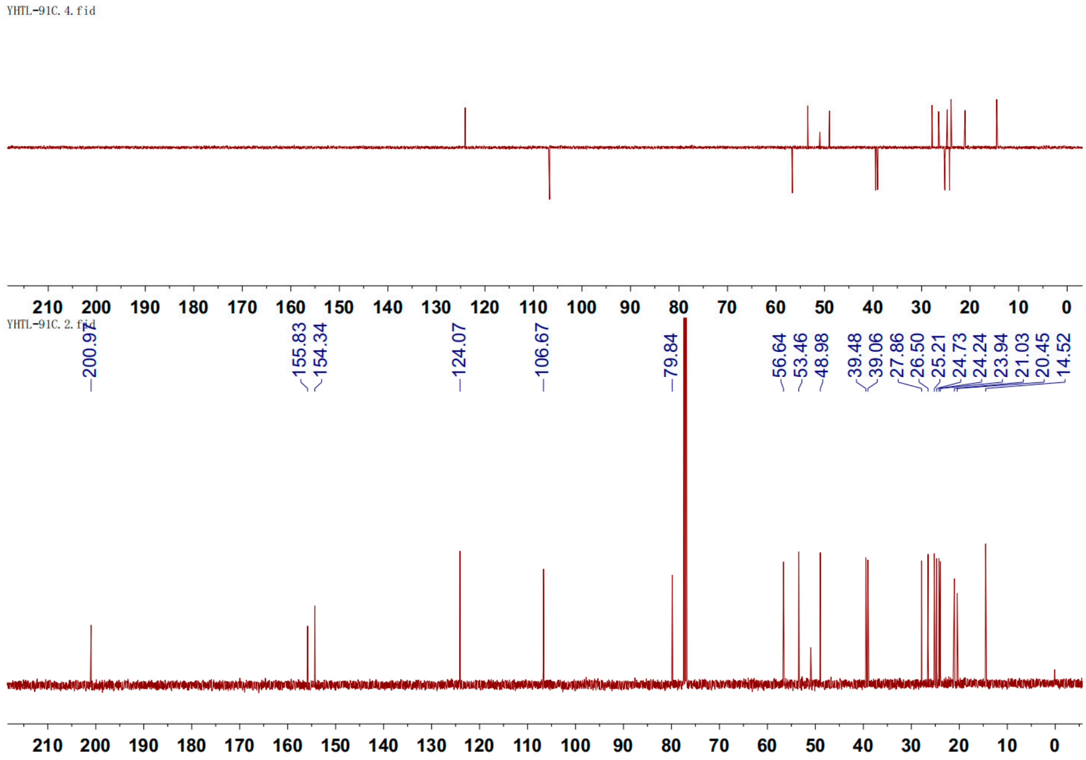

Figure S2. ^{13}C NMR and DEPT spectra of **1** in $CDCl_3$.

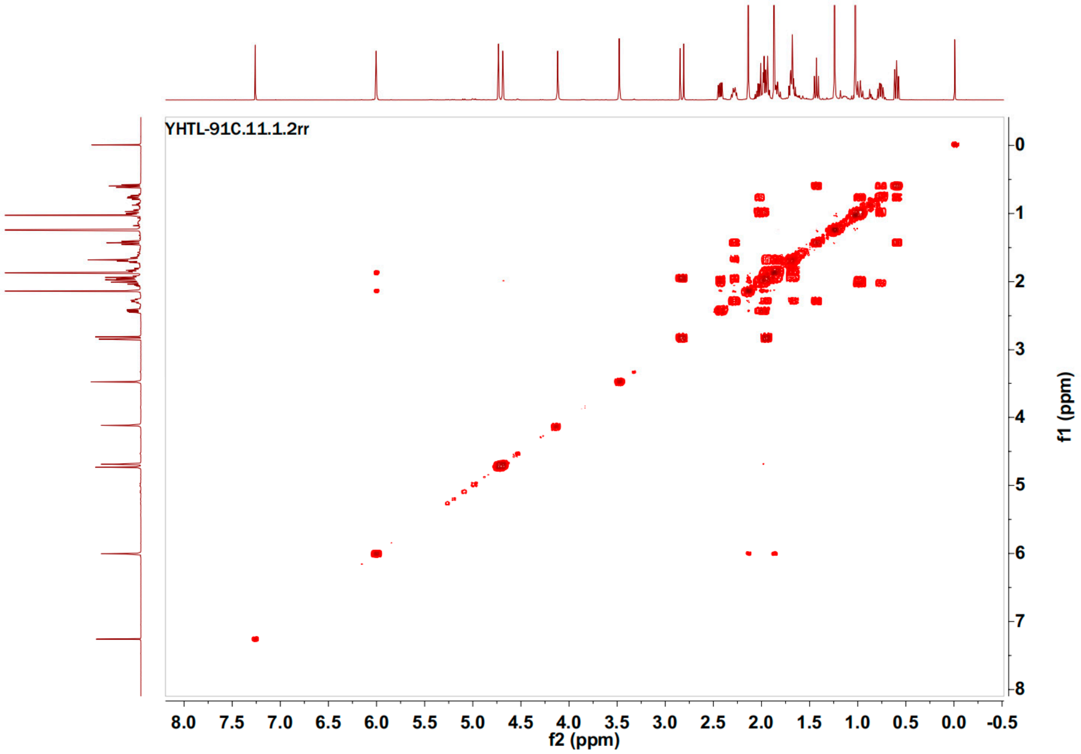

Figure S3. ^{1}H-^{1}H COSY spectrum of **1** in $CDCl_3$.

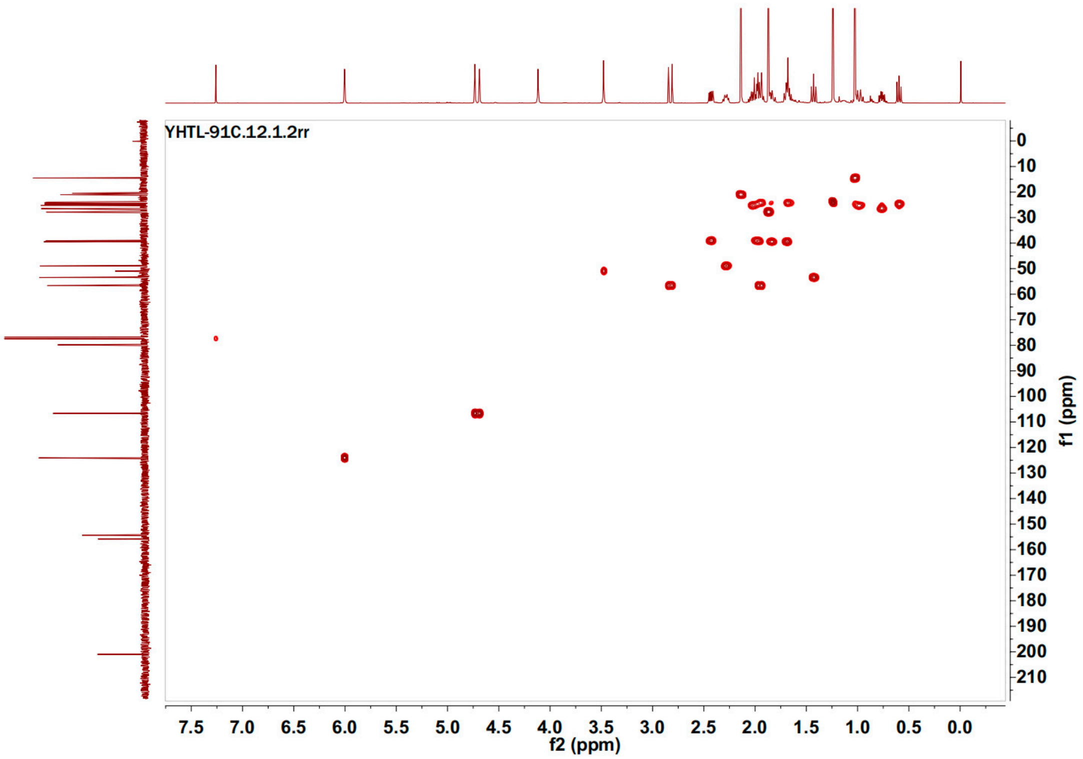

Figure S4. HSQC spectrum of **1** in $CDCl_3$.

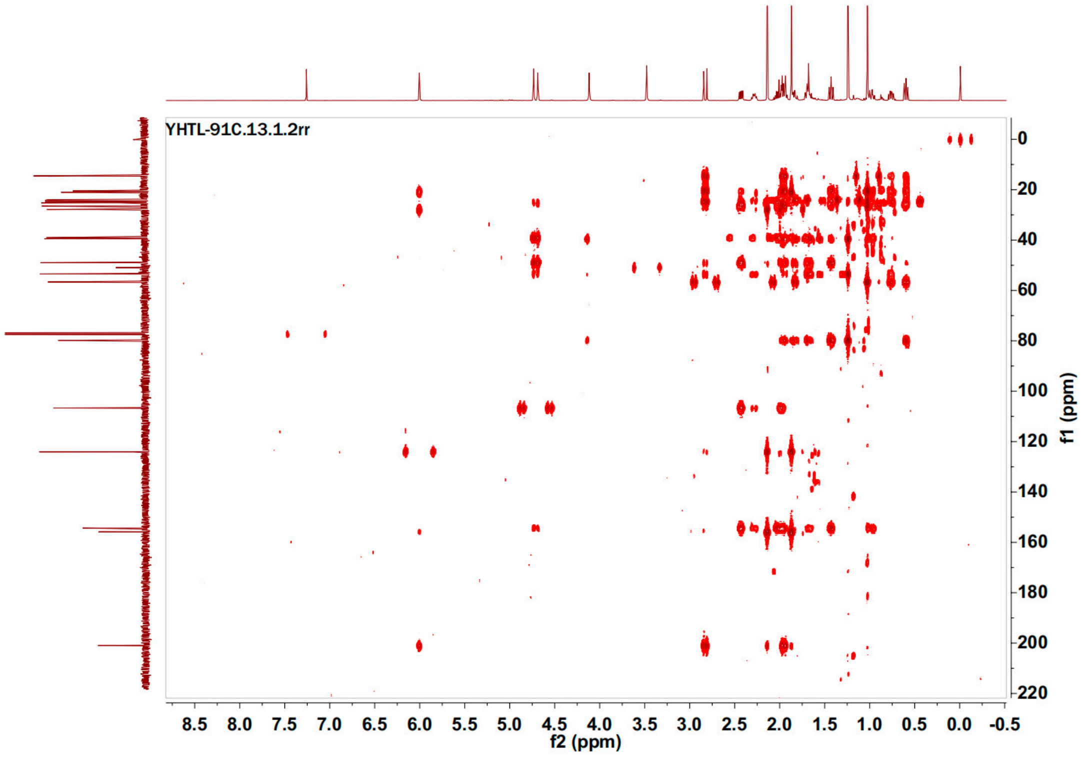

Figure S5. HMBC spectrum of **1** in $CDCl_3$.

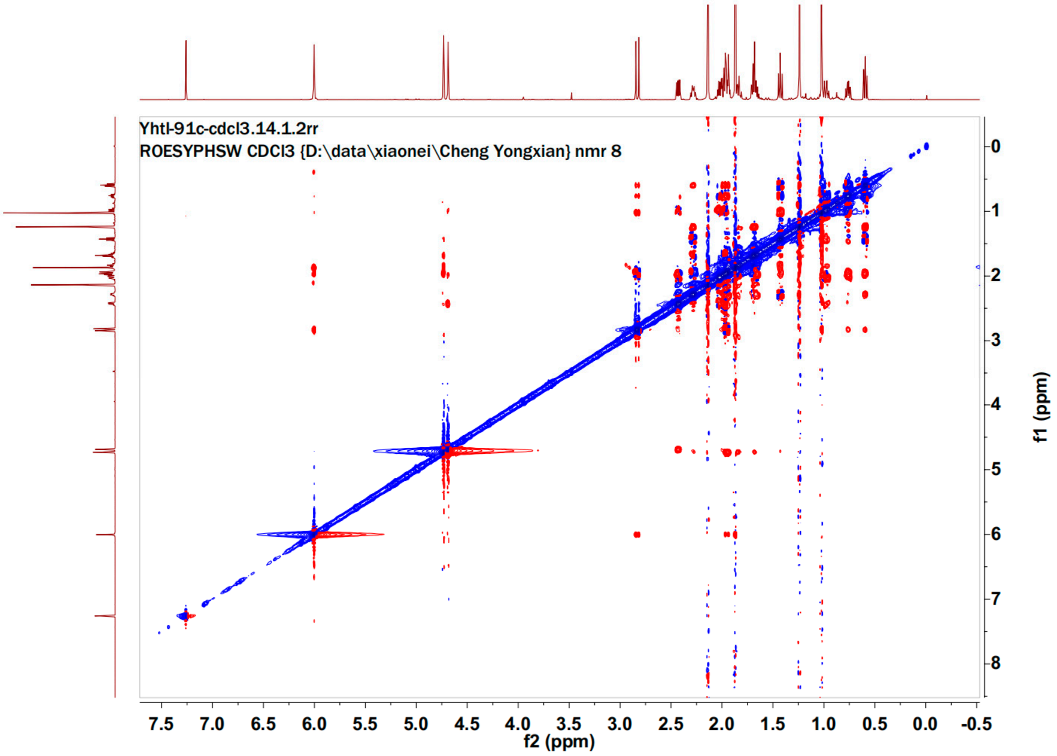

Figure S6. ROESY spectrum of **1** in $CDCl_3$.

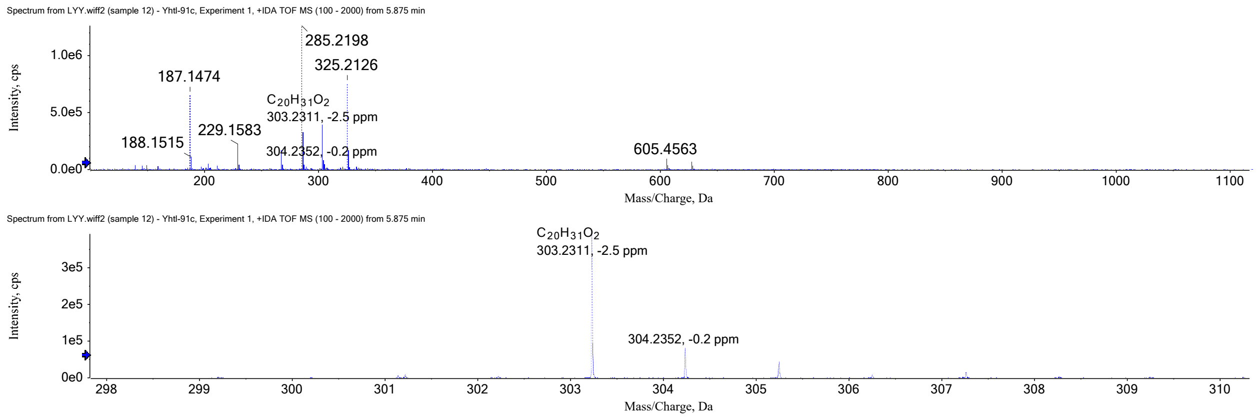

Hit	Formula	m/z	RDB	ppm	MS Rank	MSMS ppm	MSMS Rank	Found
1	C20H30O2	303.2319	6.0	-2.5	1			NA/NA

Figure S7. HRESIMS of **1**.

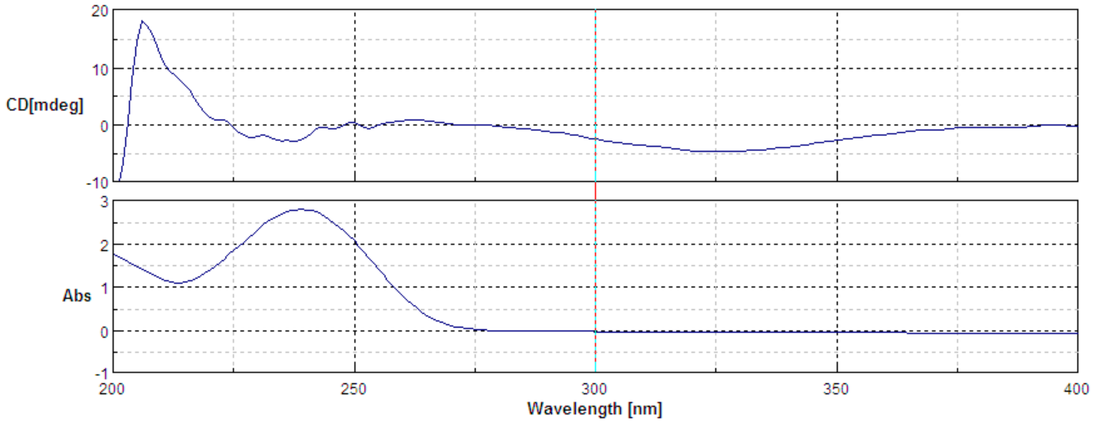

Figure S8. CD and UV spectra of **1**.

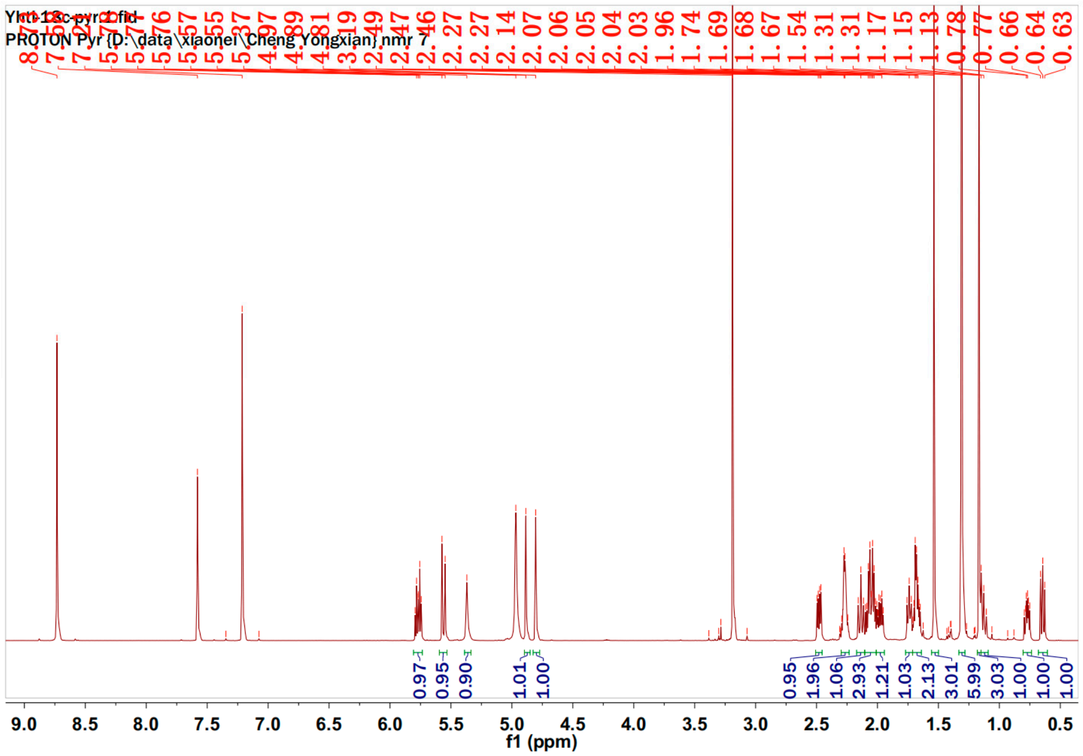

Figure S9. ^{1}H NMR spectrum of **2** in Pyridine-d_5.

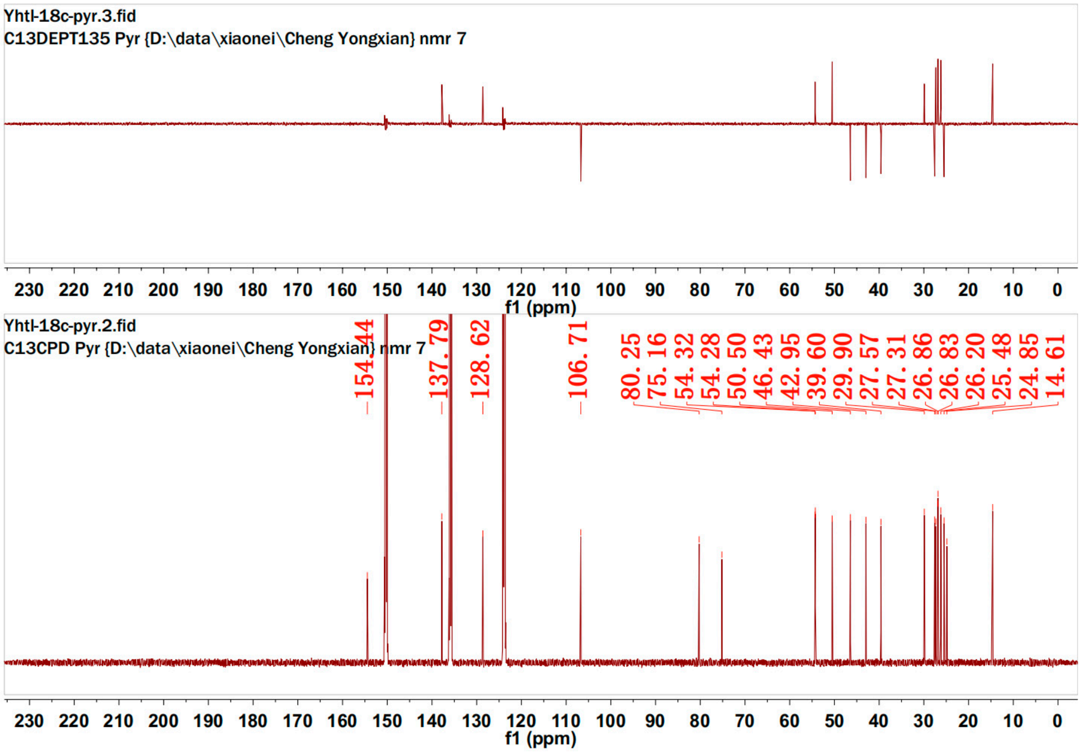

Figure S10. ^{13}C NMR and DEPT spectra of **2** in Pyridine-d_5.

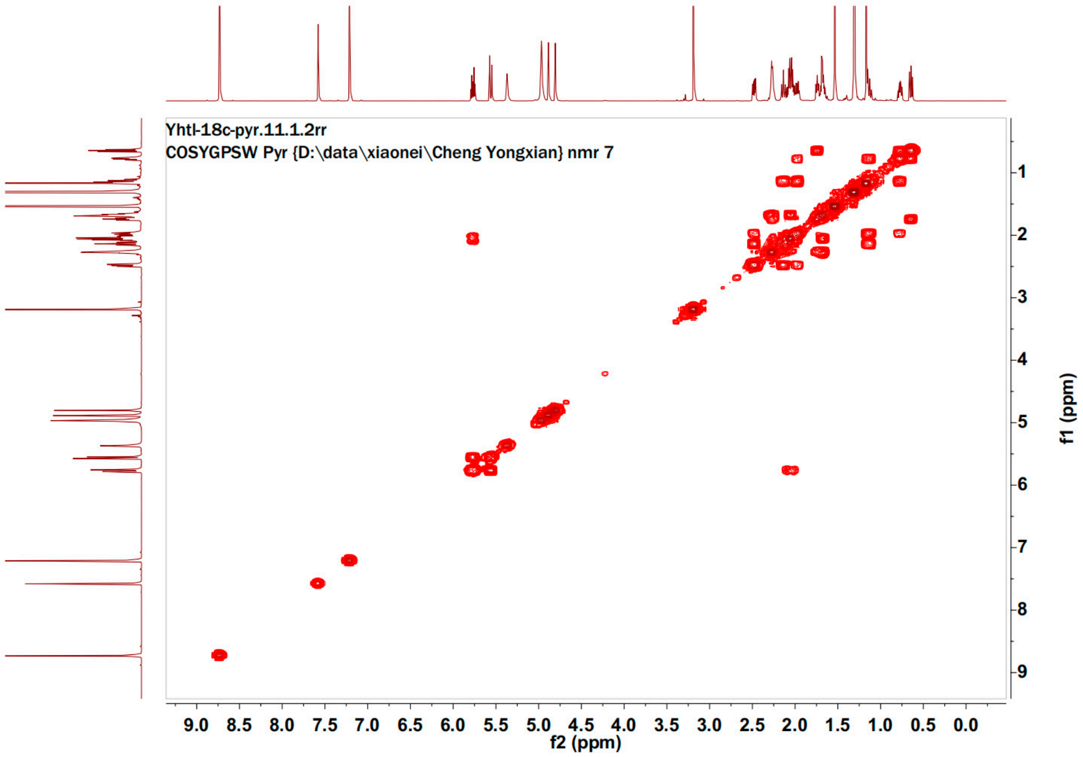

Figure S11. ^{1}H-^{1}H COSY spectrum of **2** in Pyridine-d_5.

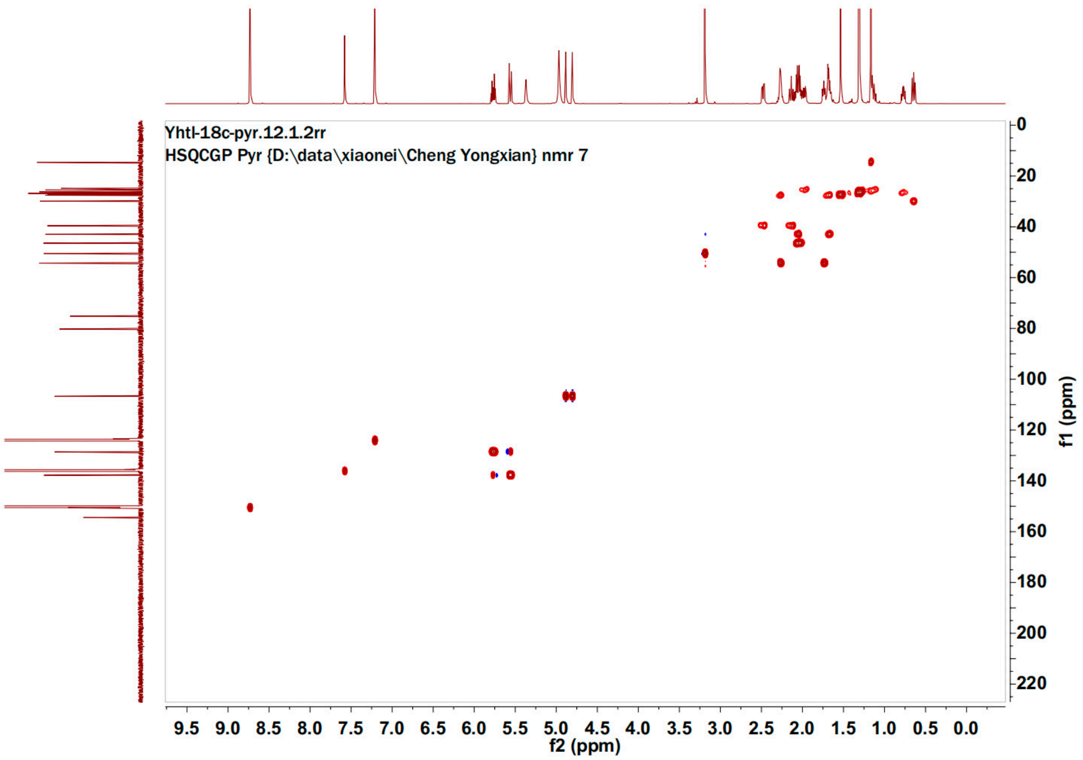

Figure S12. HSQC spectrum of **2** in Pyridine-*d*5.

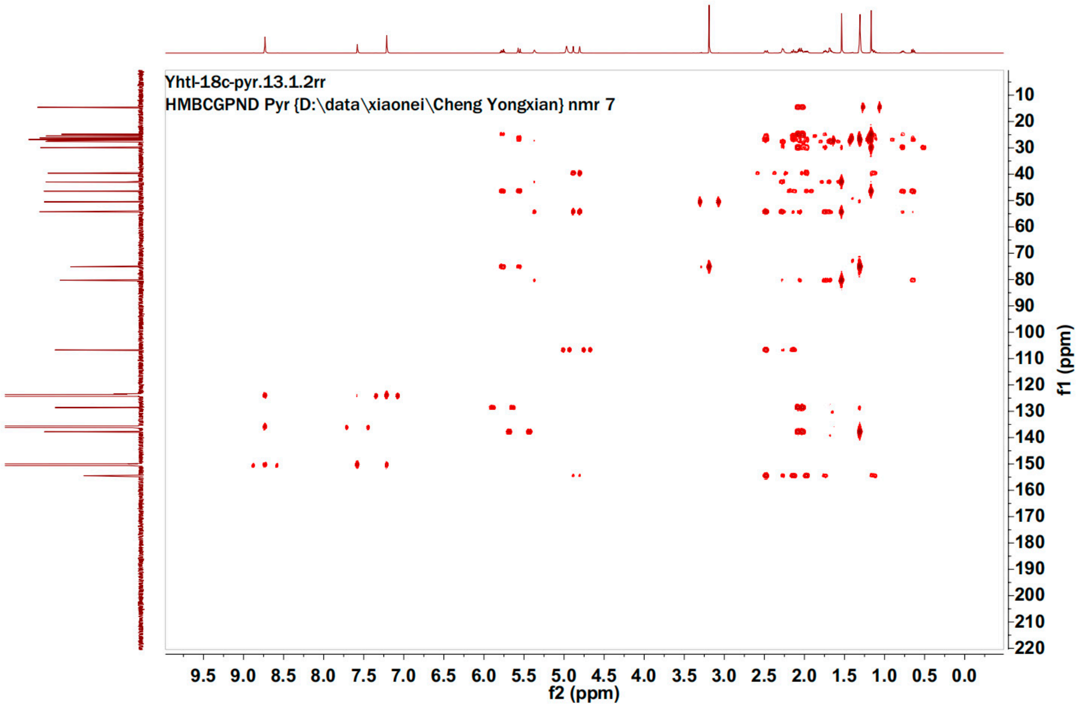

Figure S13. HMBC spectrum of **2** in Pyridine-*d*5.

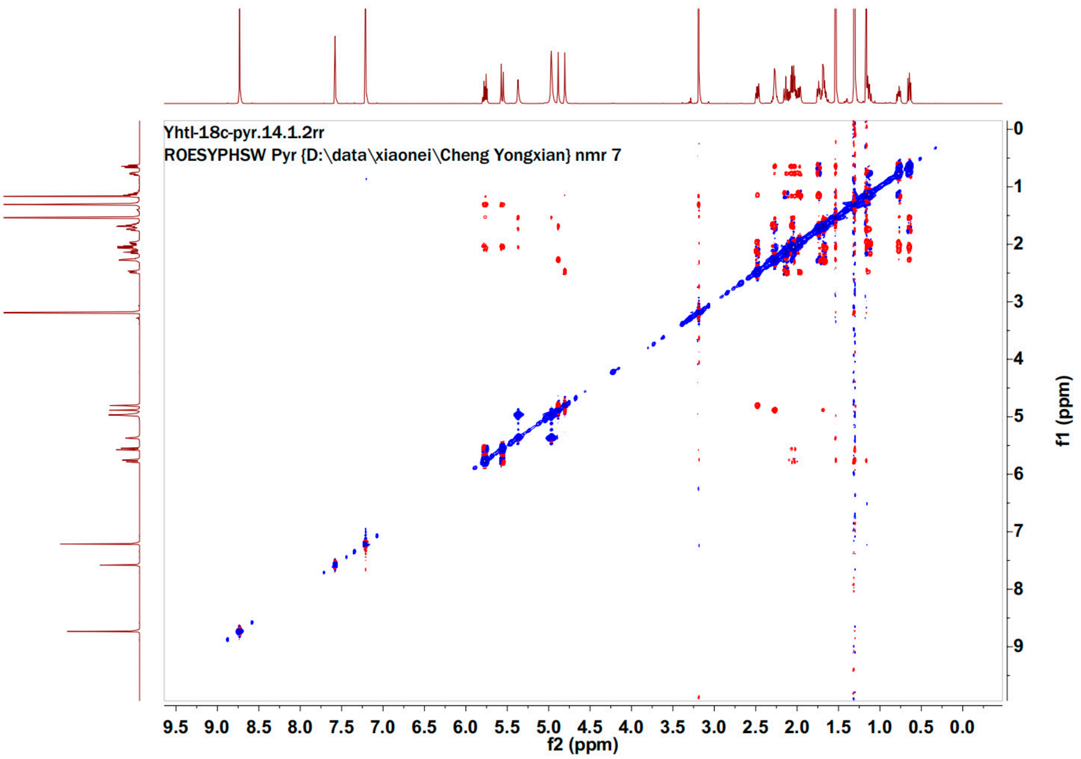

Figure S14. ROESY spectrum of **2** in Pyridine-d_5.

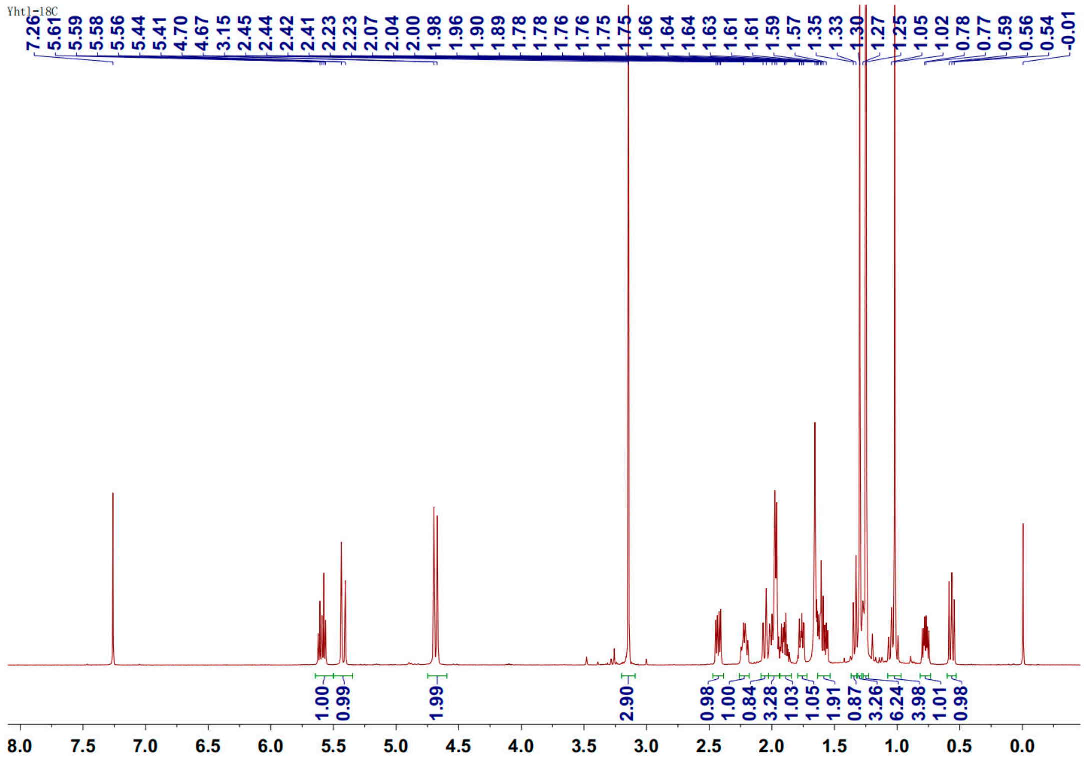

Figure S15. ^{1}H NMR spectrum of **2** in $CDCl_3$.

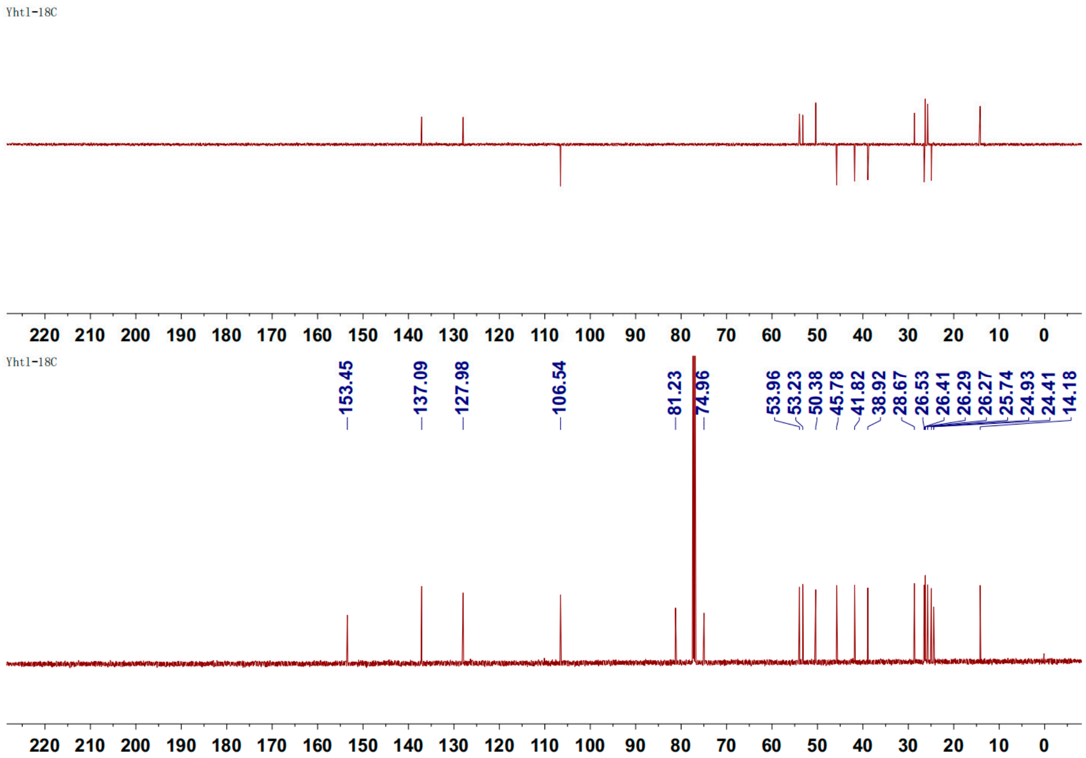

Figure S16. ^{13}C NMR and DEPT spectra of **2** in $CDCl_3$.

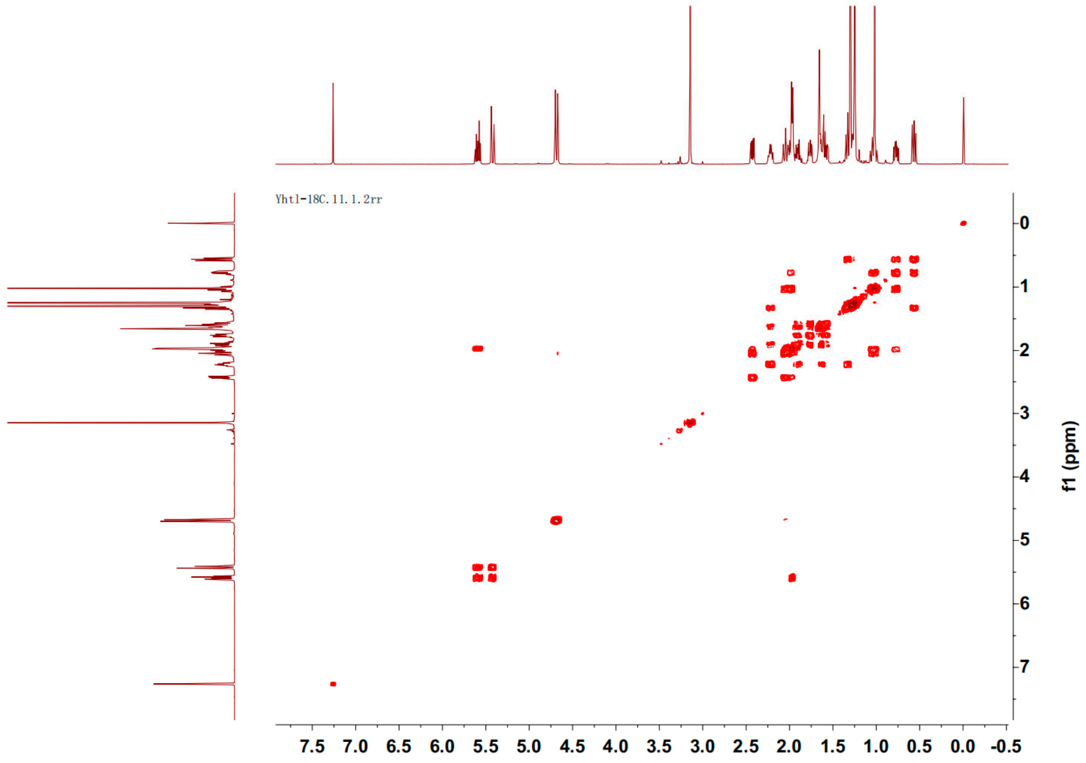

Figure S17. ^{1}H-^{1}H COSY spectrum of **2** in $CDCl_3$.

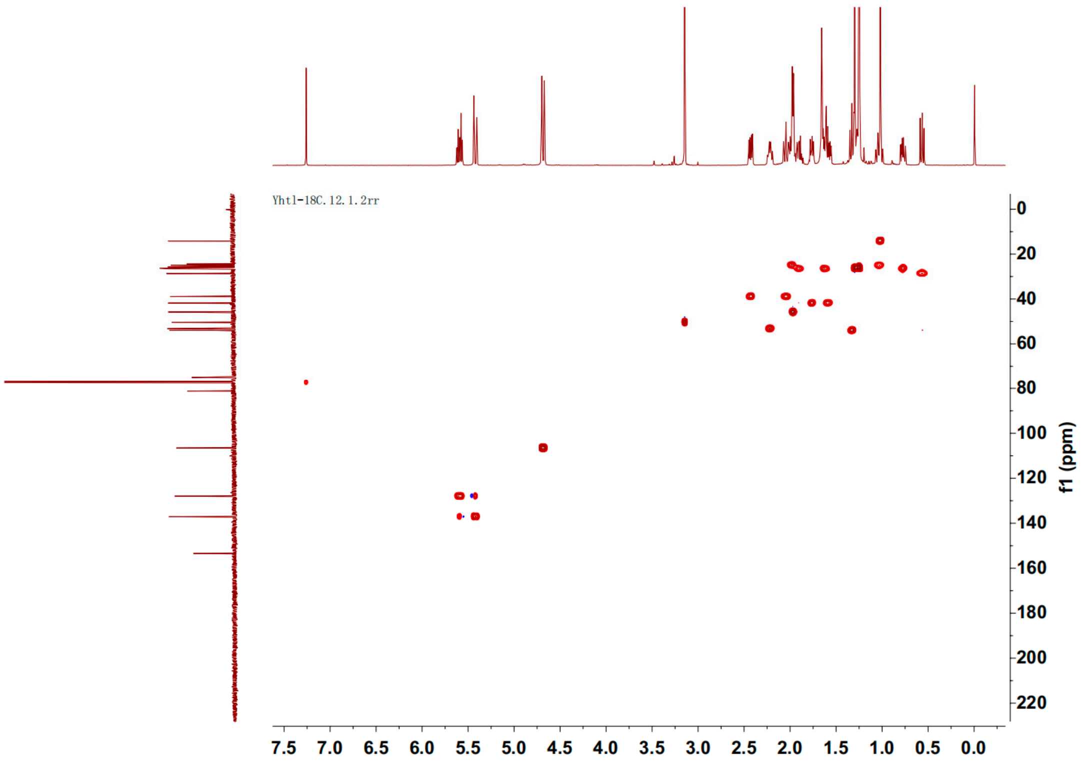

Figure S18. HSQC spectrum of **2** in $CDCl_3$.

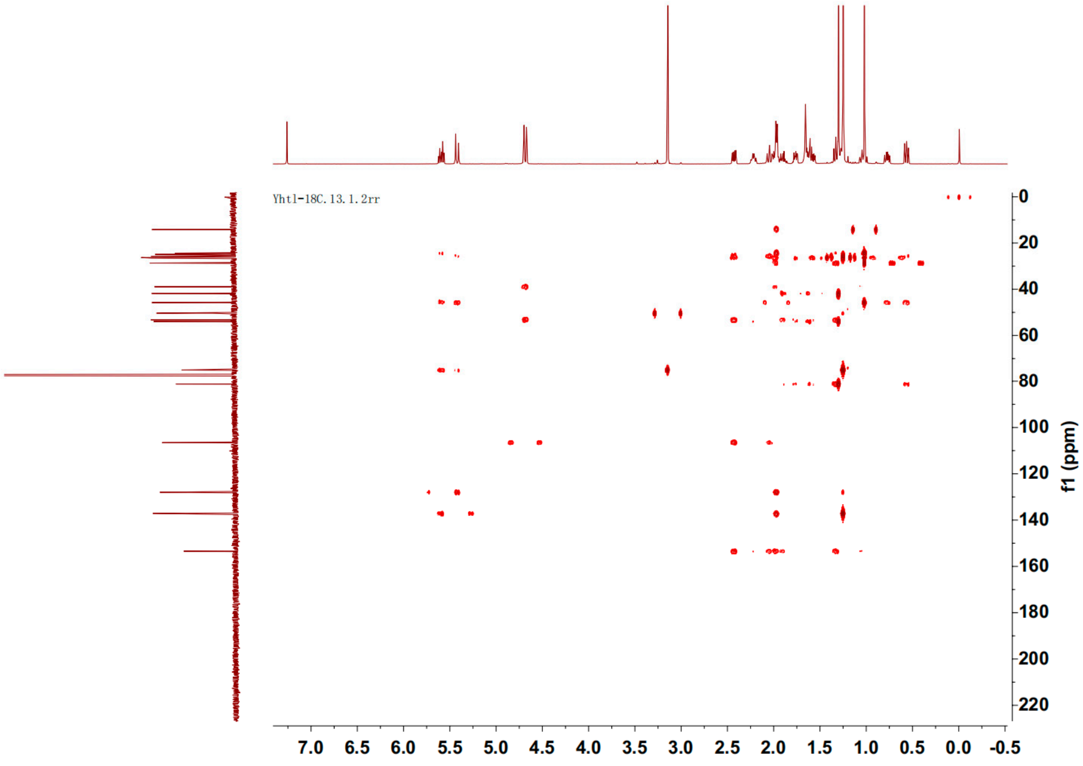

Figure S19. HMBC spectrum of **2** in $CDCl_3$.

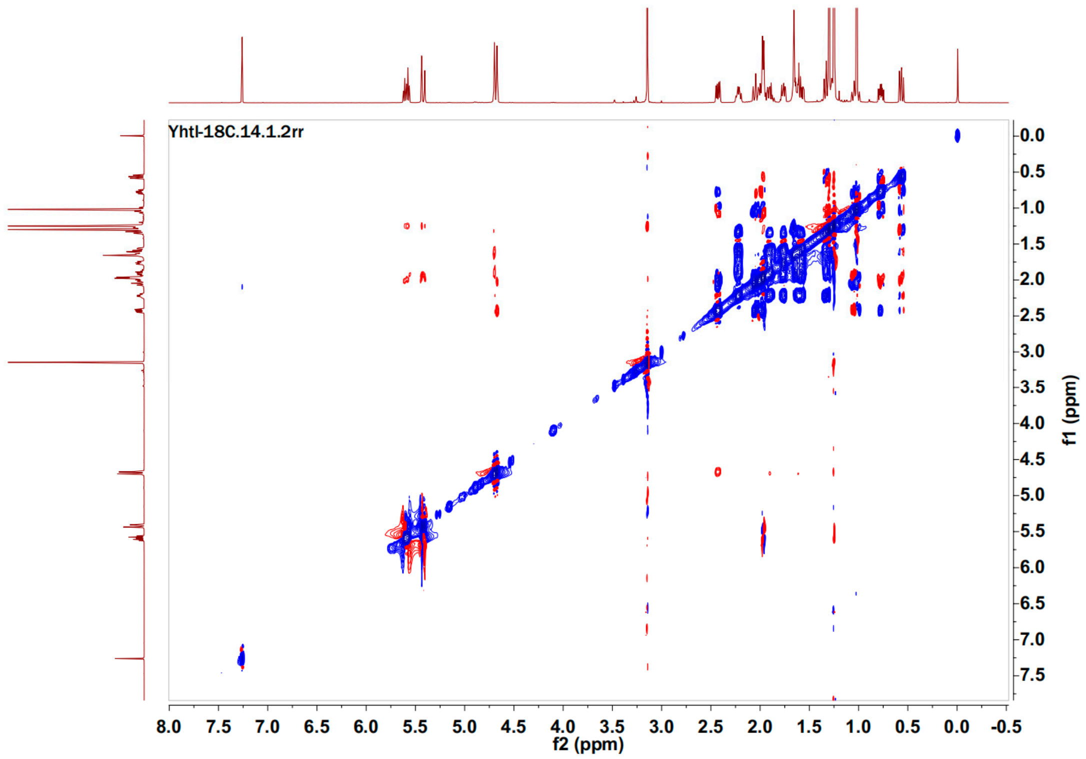

Figure S20. ROESY spectrum of **2** in $CDCl_3$.

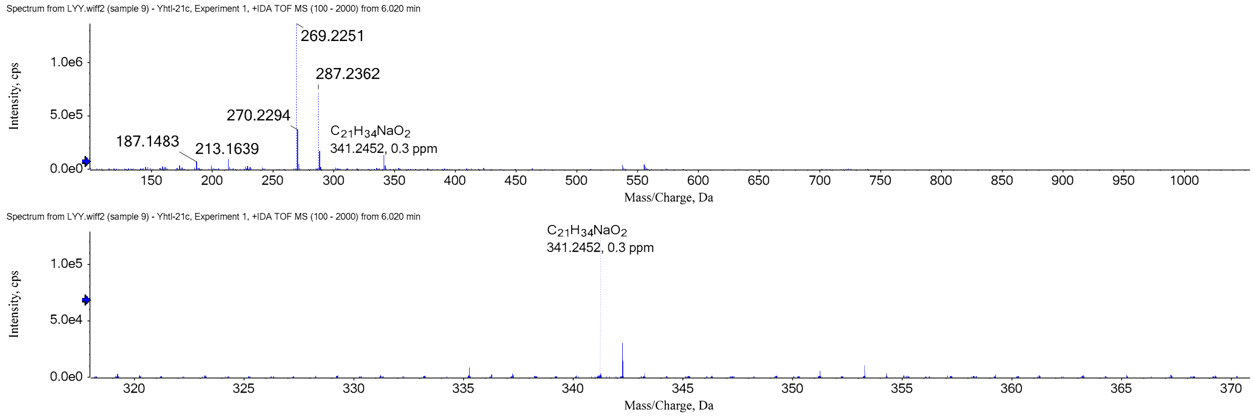

Hit	Formula	m/z	RDB	ppm	MS Rank	MSMS ppm	MSMS Rank	Found
1	C21H34O2	341.2451	5.0	0.3	1			NA/NA

Figure S21. HREIMS of **2**.

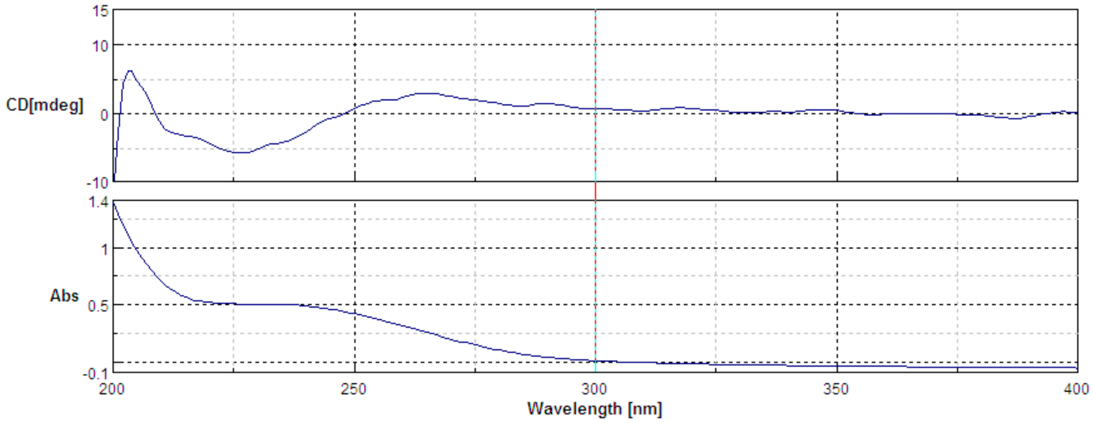

Figure S22. CD and UV spectra of **2**.

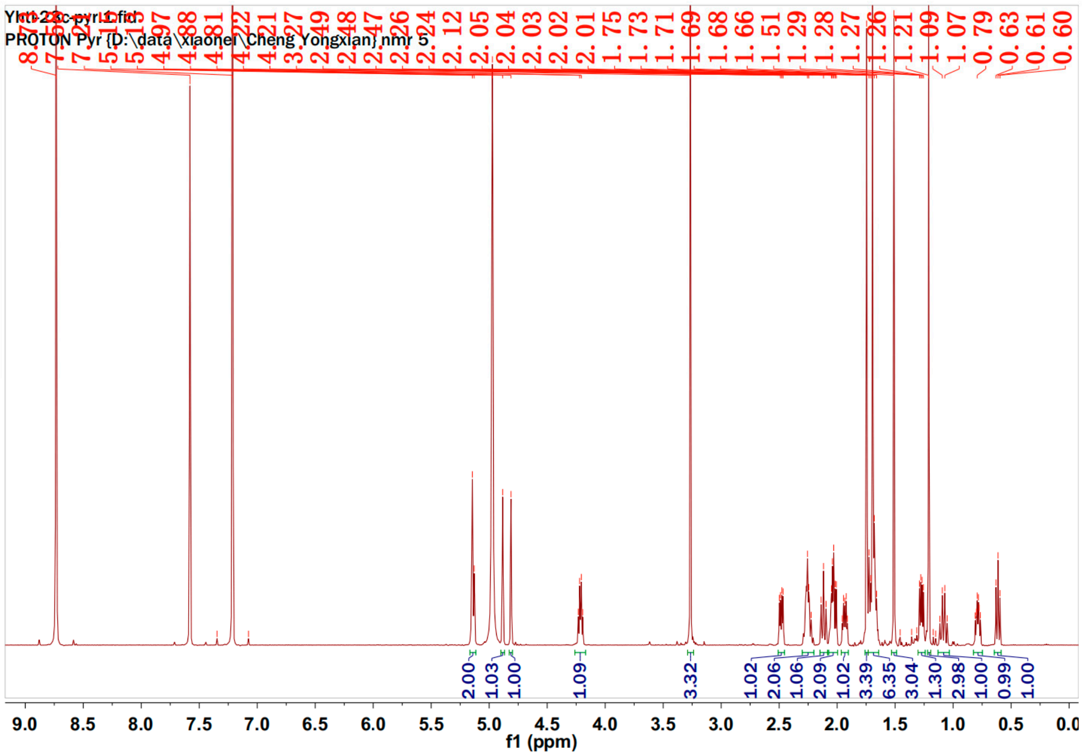

Figure S23. ^{1}H NMR spectrum of **3** in Pyridine-d_5.

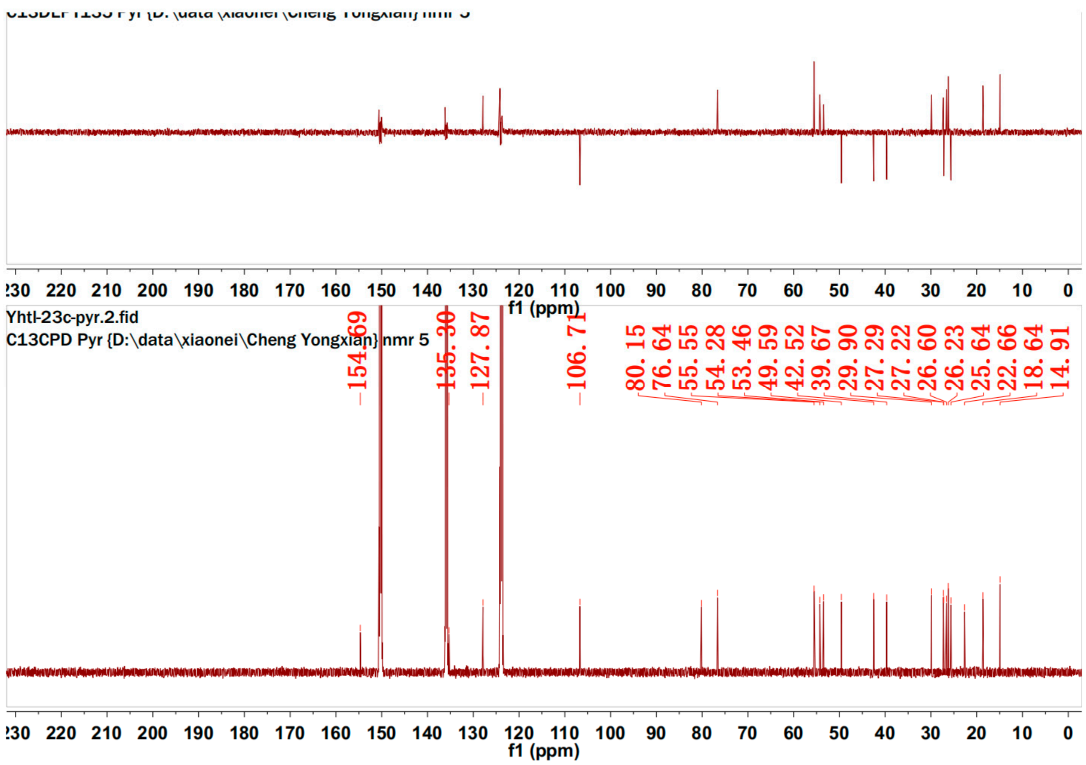

Figure S24. ^{13}C NMR and DEPT spectra of **3** in Pyridine-*d*$_5$.

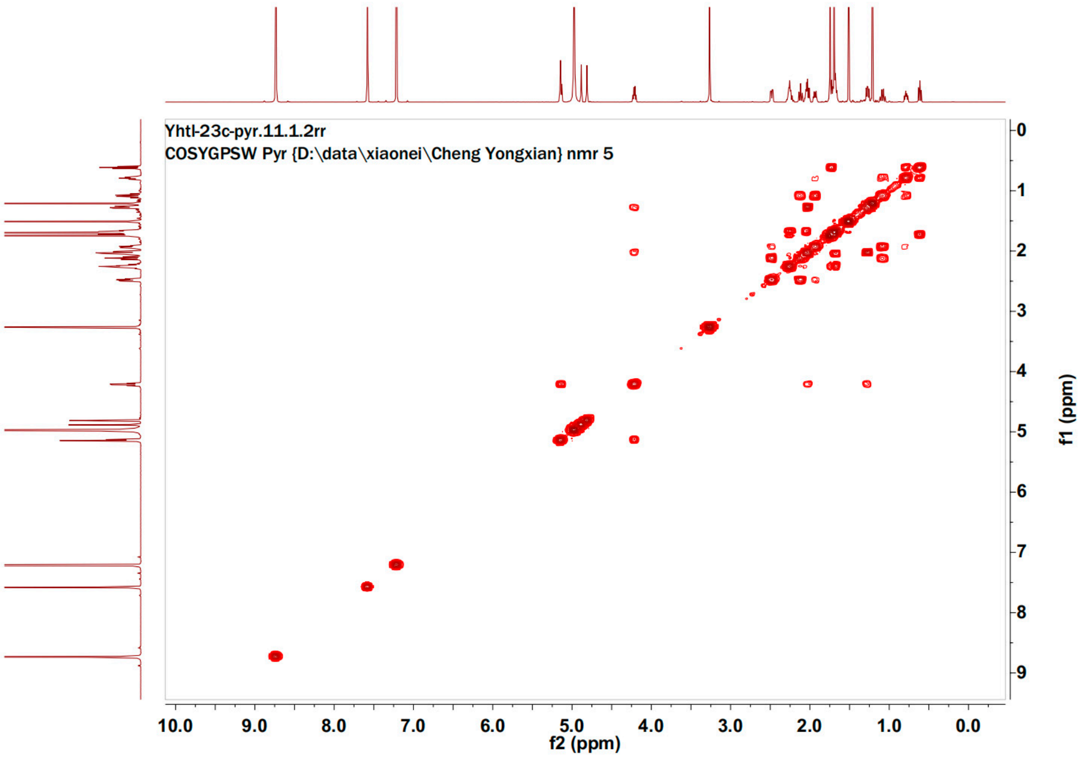

Figure S25. ^{1}H-^{1}H COSY spectrum of **3** in Pyridine-*d*$_5$.

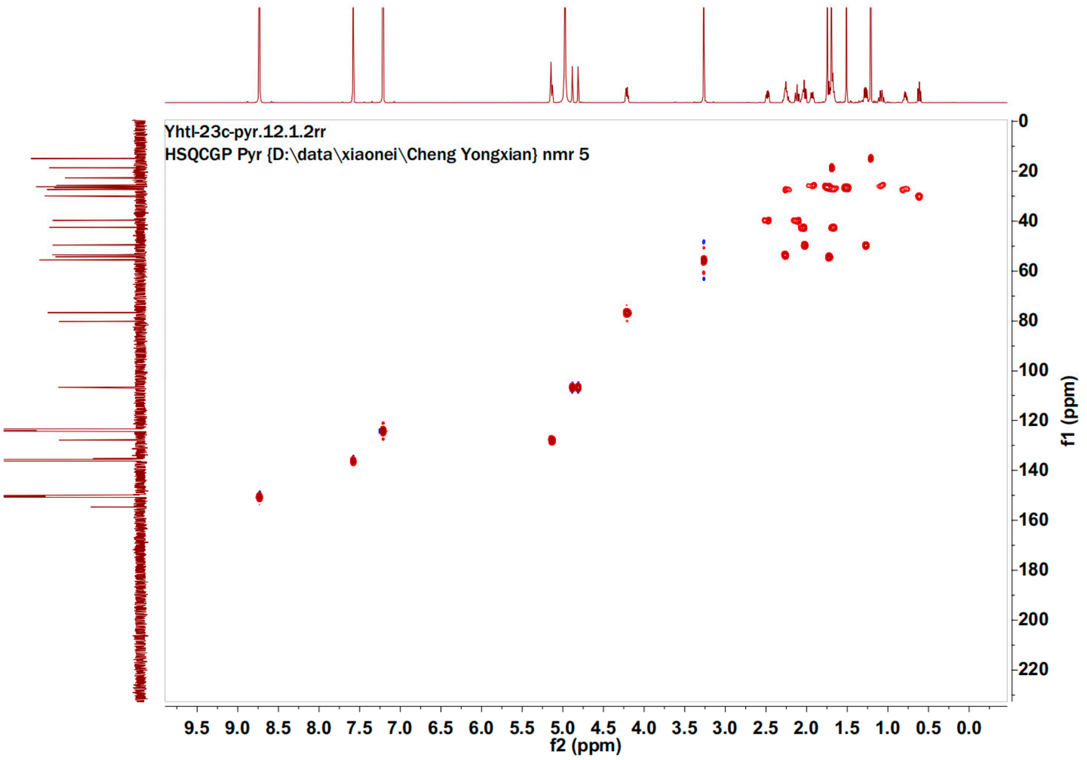

Figure S26. HSQC spectrum of **3** in Pyridine-d_5.

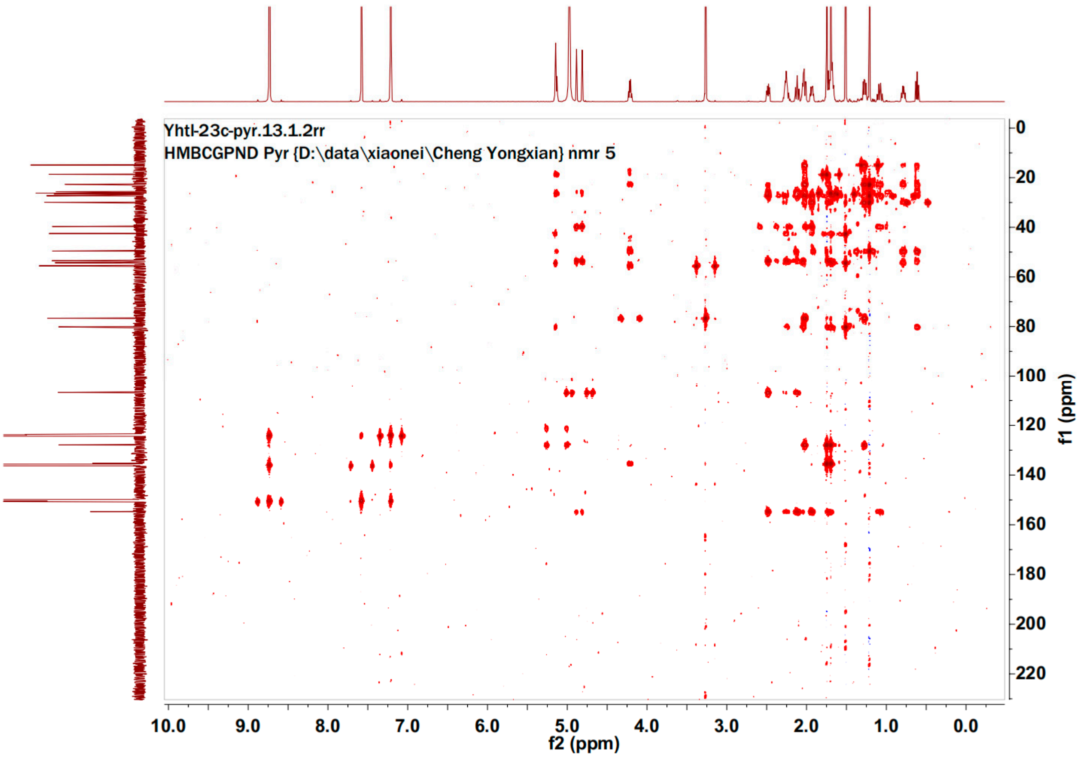

Figure S27. HMBC spectrum of **3** in Pyridine-d_5.

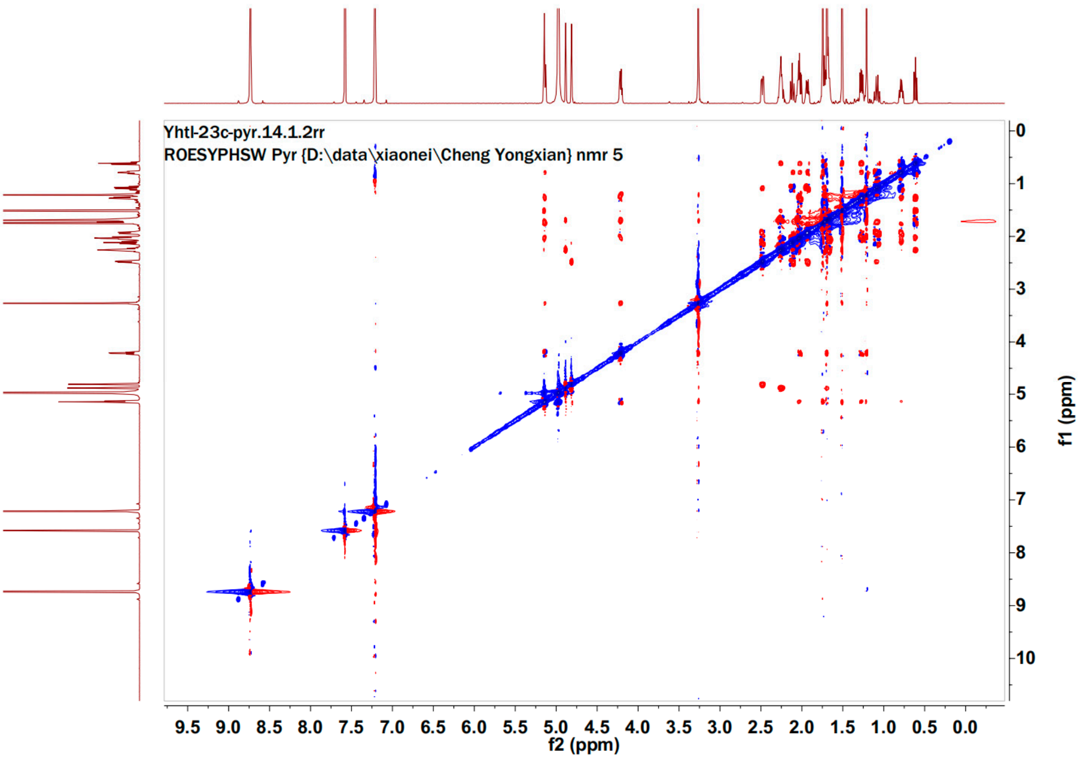

Figure S28. ROESY spectrum of **3** in Pyridine-d_5.

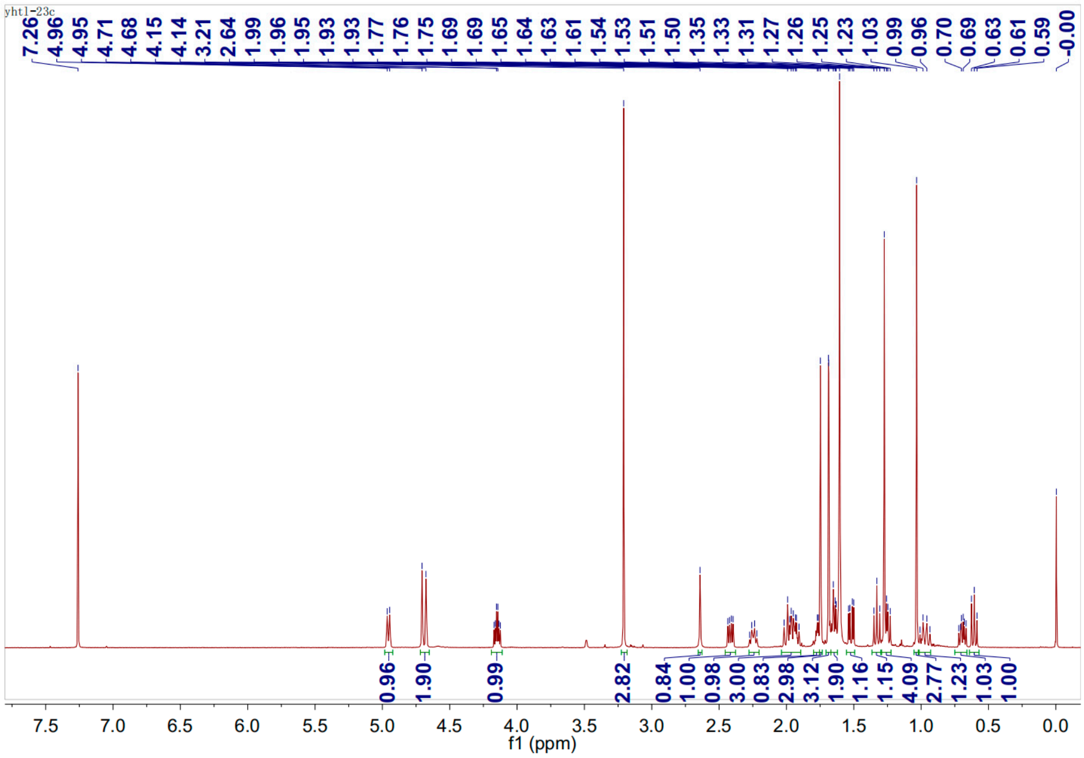

Figure S29. ^{1}H NMR spectrum of **3** in $CDCl_3$.

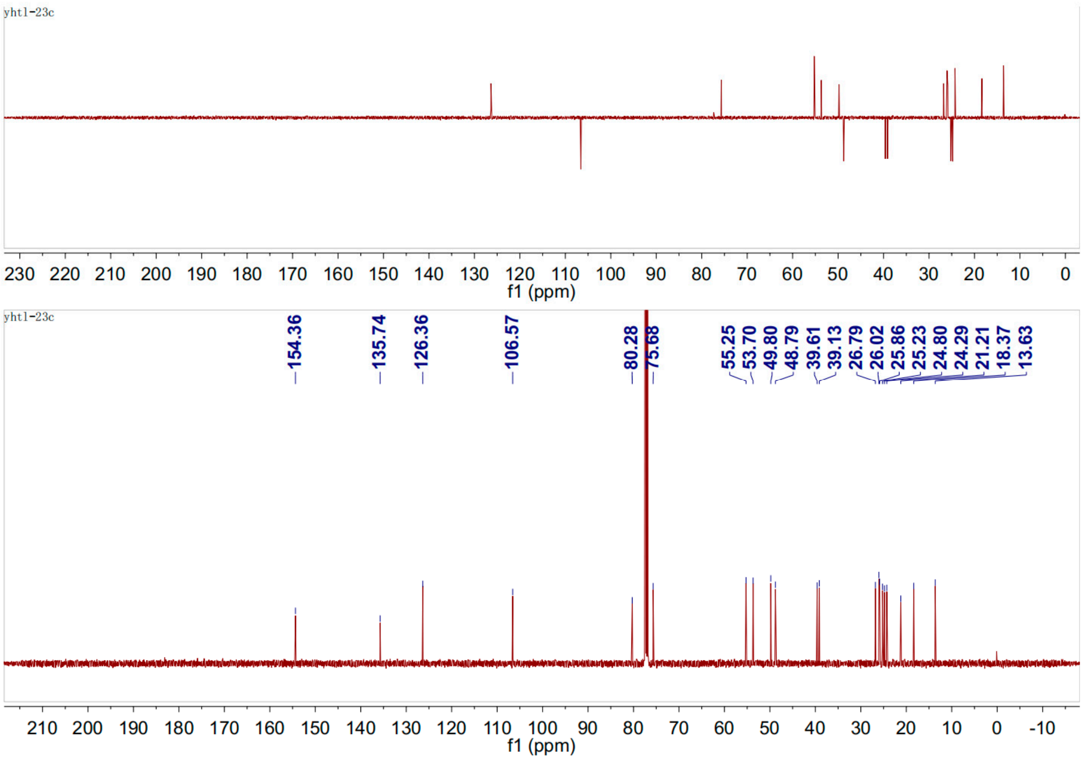

Figure S30. ^{13}C NMR and DEPT spectra of **3** in $CDCl_3$.

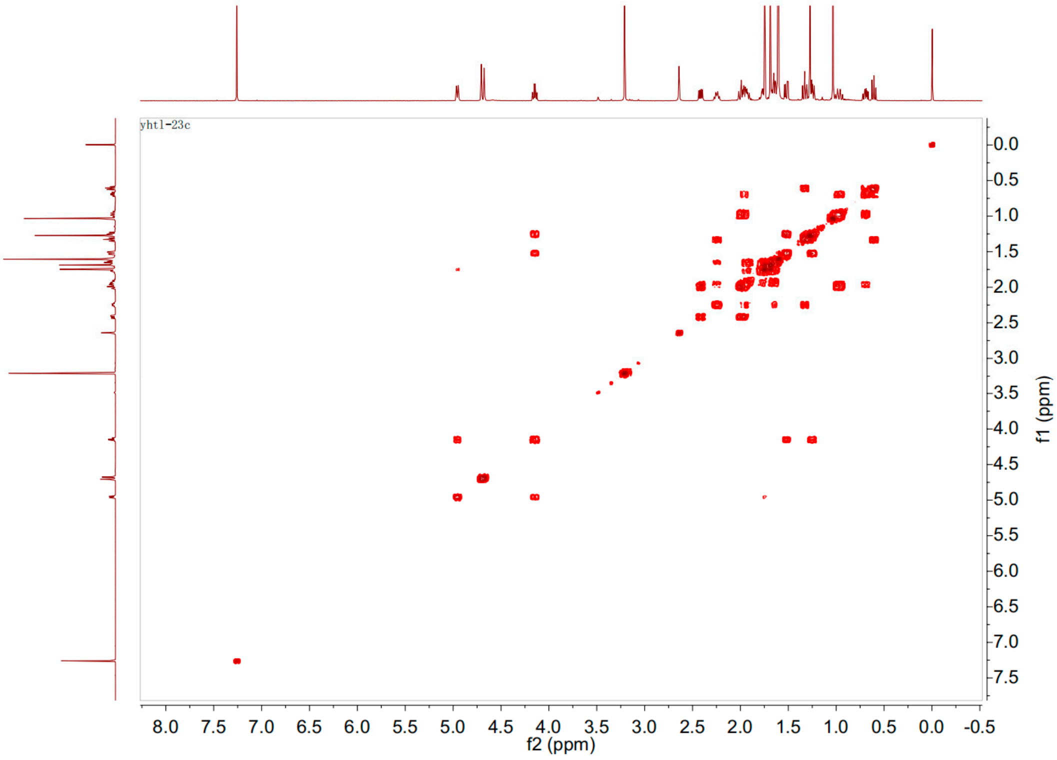

Figure S31. ^{1}H-^{1}H COSY spectrum of **3** in $CDCl_3$.

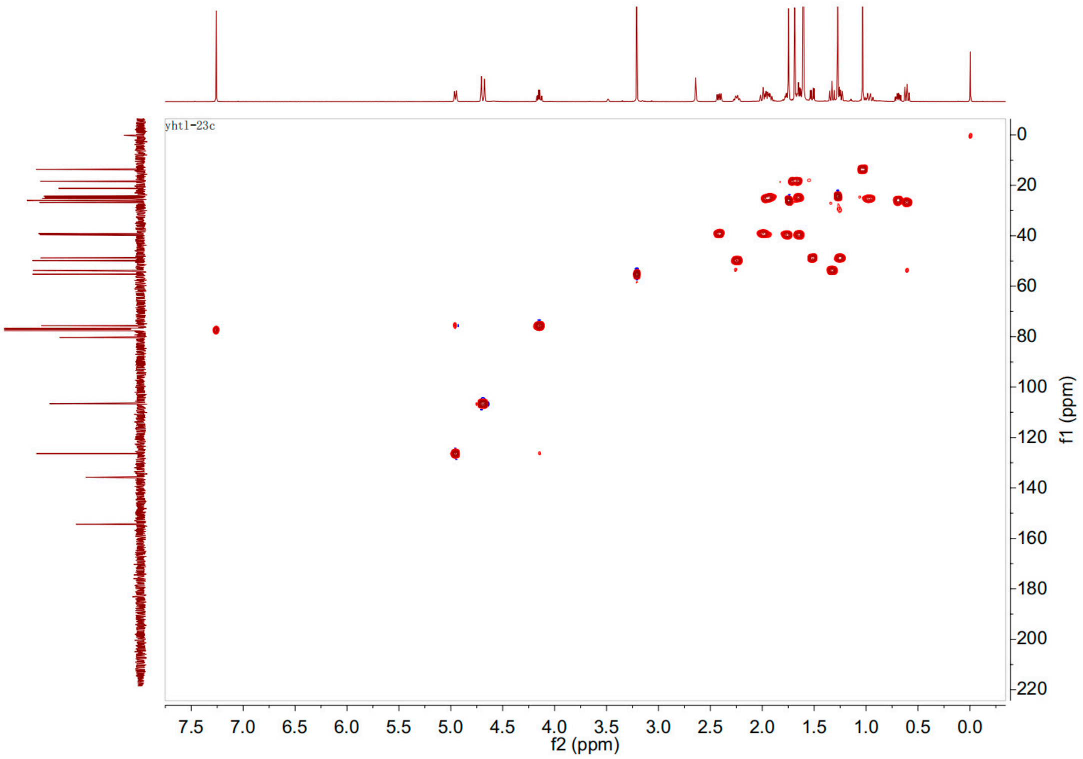

Figure S32. HSQC spectrum of **3** in $CDCl_3$.

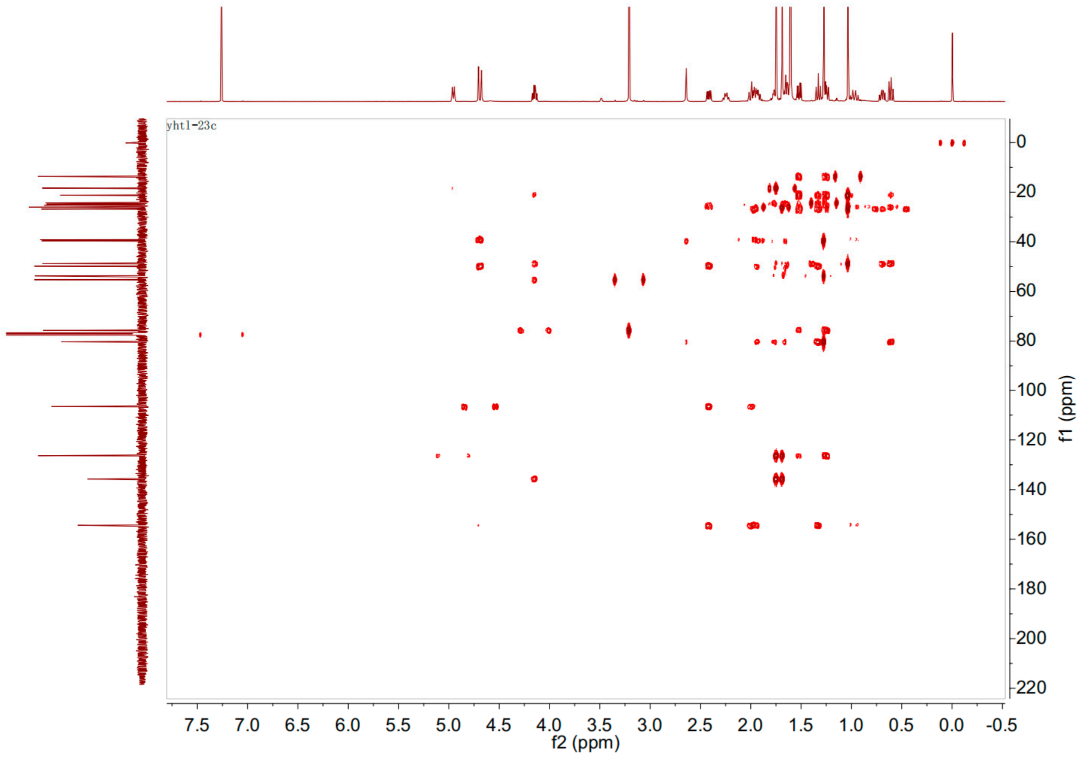

Figure S33. HMBC spectrum of **3** in $CDCl_3$.

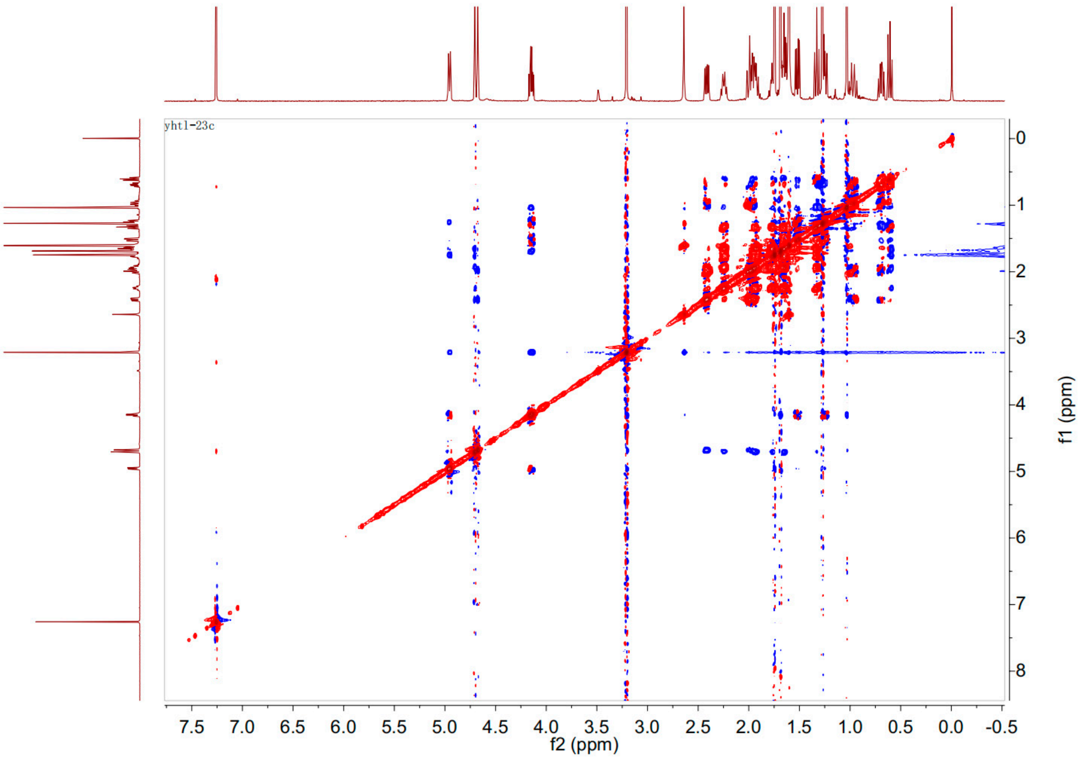

Figure S34. ROESY spectrum of **3** in $CDCl_3$.

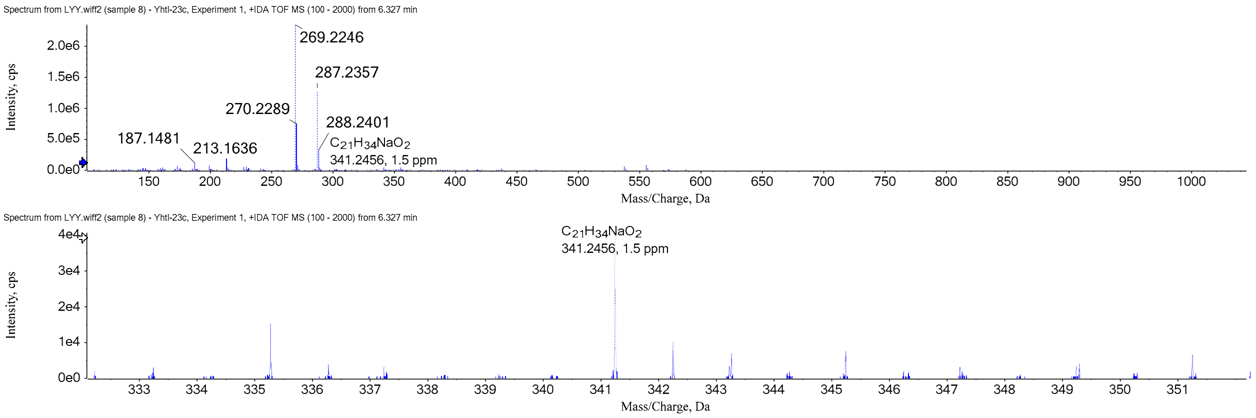

Hit	Formula	m/z	RDB	ppm	MS Rank	MSMS ppm	MSMS Rank	Found
1	C21H34O2	341.2451	5.0	1.5	1			NA/NA

Figure S35. HREIMS of **3**.

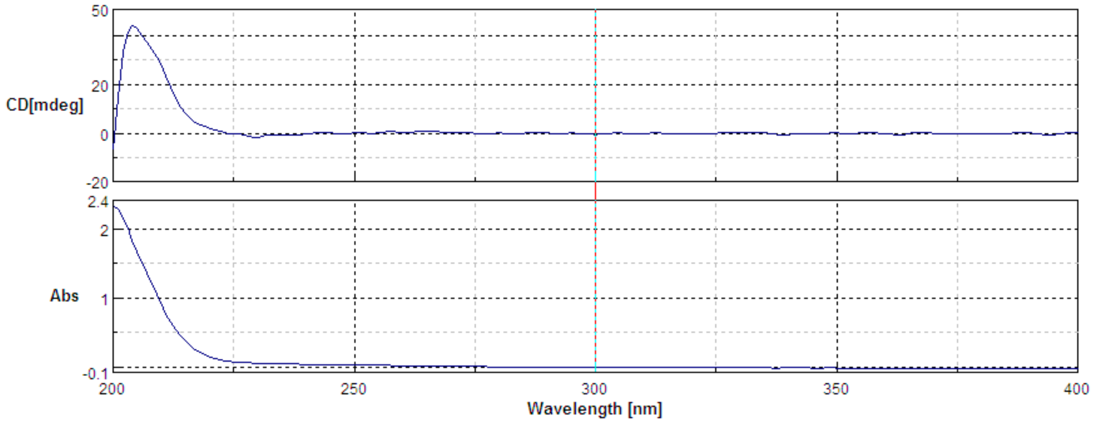

Figure S36. CD and UV spectra of **3**.

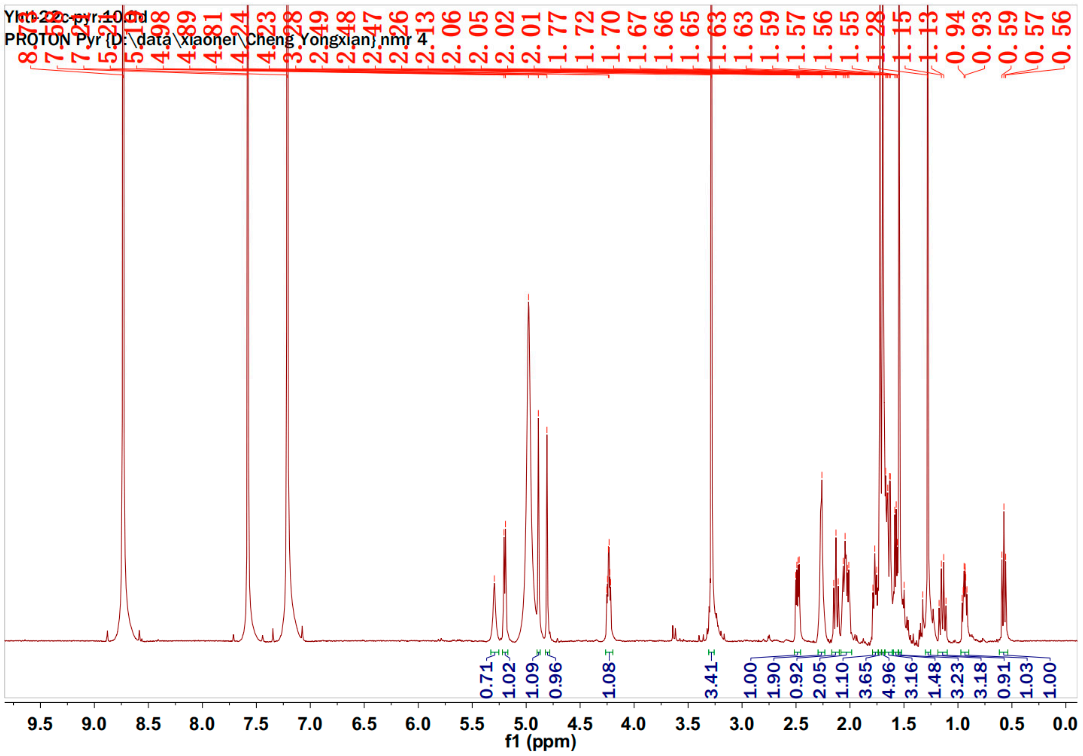

Figure S37. ^{1}H NMR spectrum of **4** in Pyridine-*d*5.

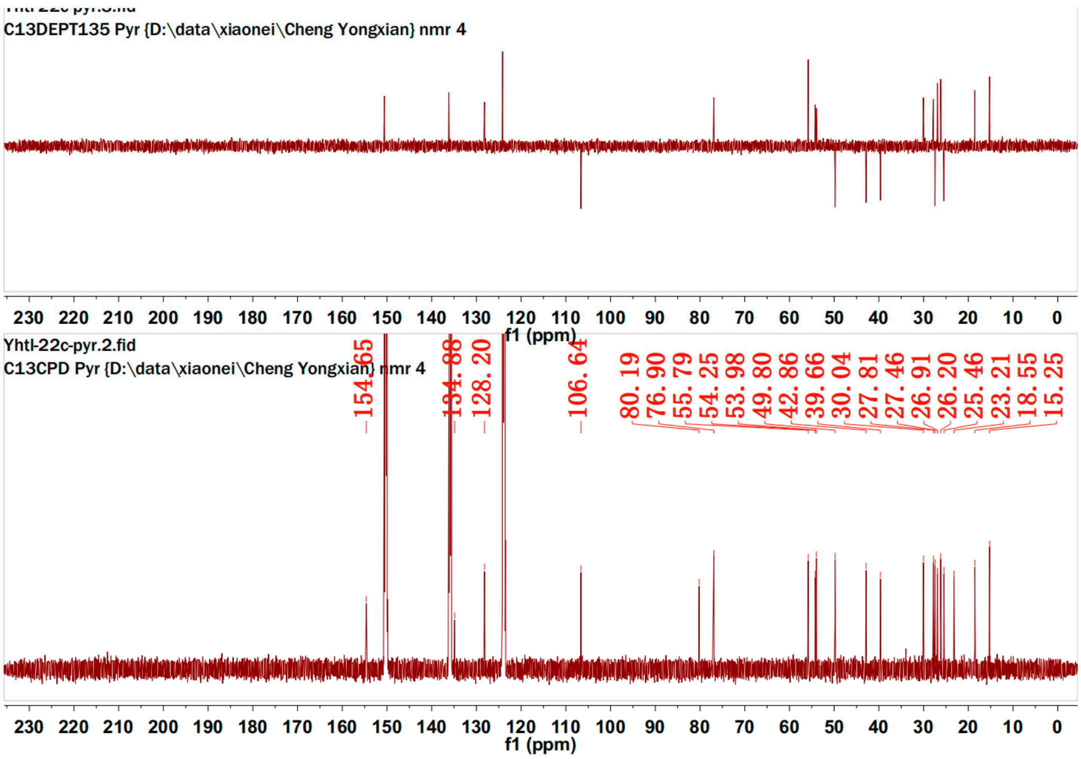

Figure S38. ^{13}C NMR and DEPT spectra of **4** in Pyridine-d_5.

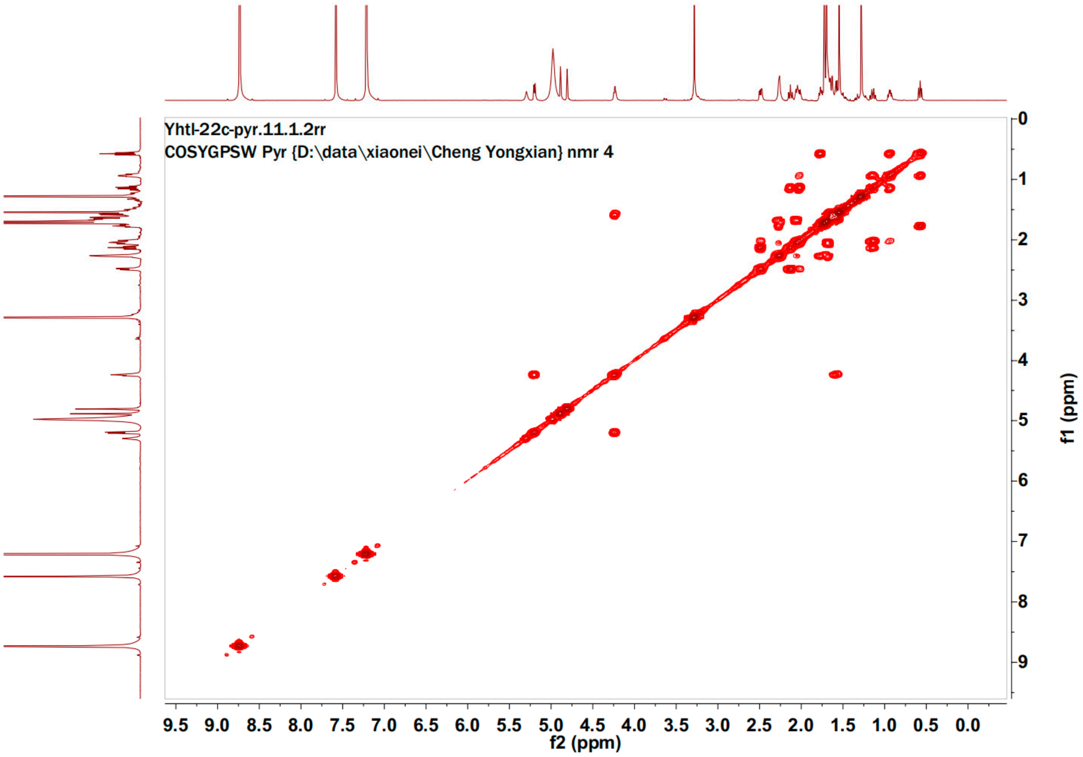

Figure S39. ^{1}H-^{1}H COSY spectrum of **4** in Pyridine-d_5.

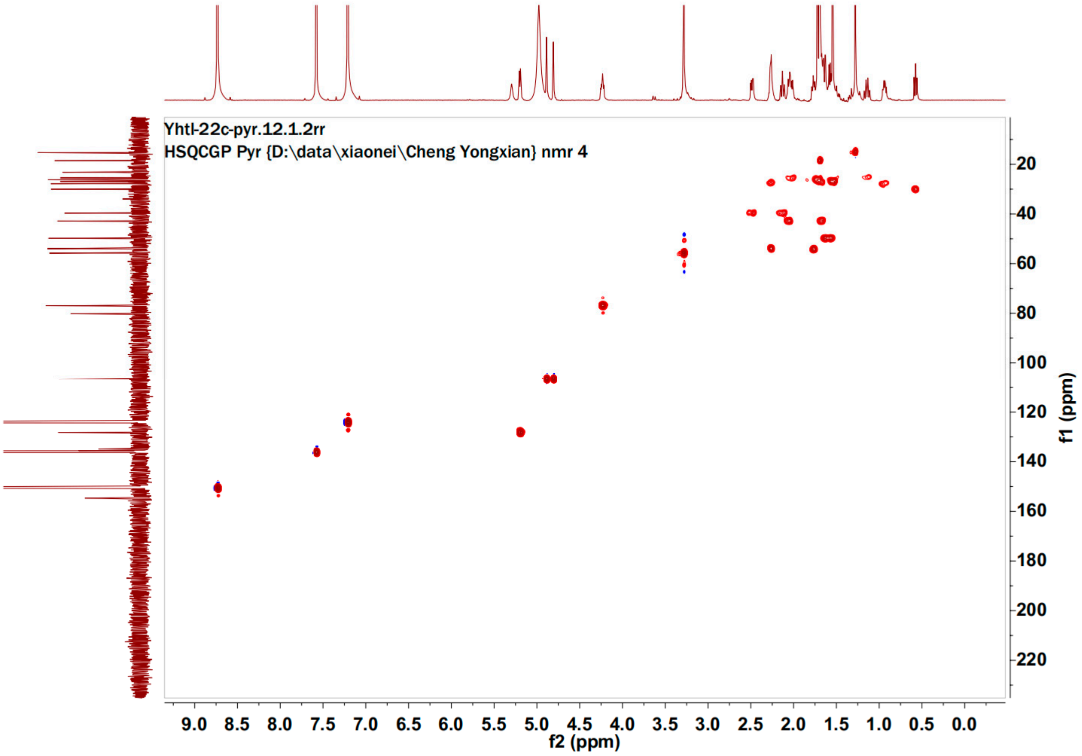

Figure S40. HSQC spectrum of **4** in Pyridine-d_5.

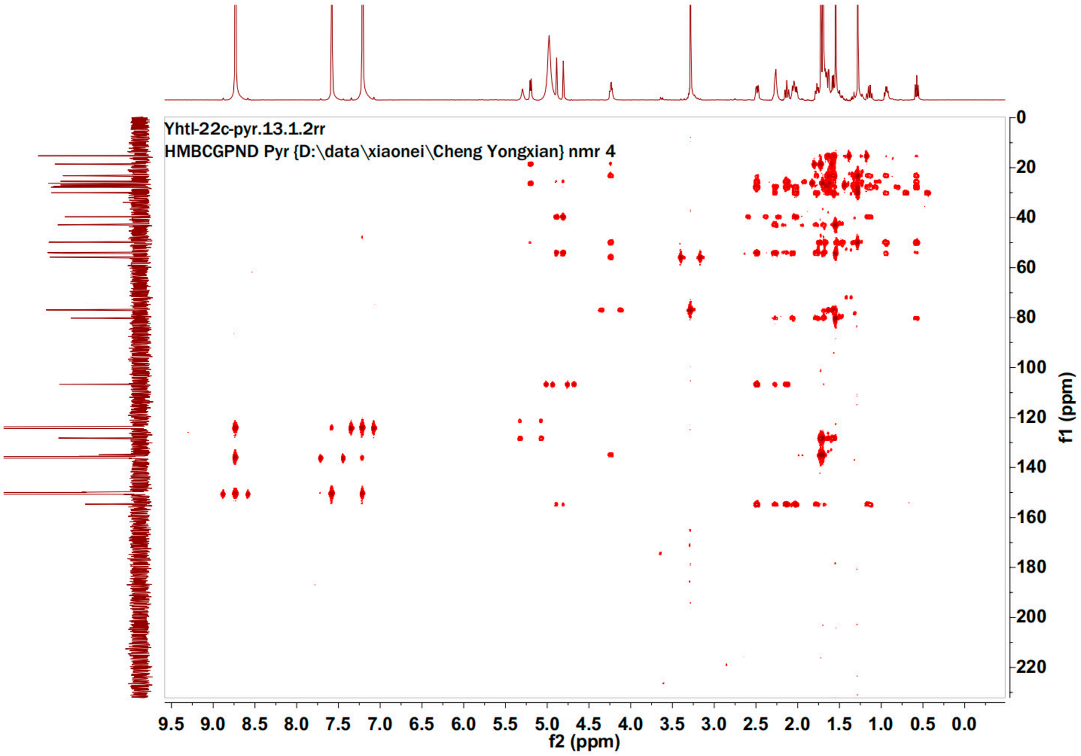

Figure S41. HMBC spectrum of **4** in Pyridine-d_5.

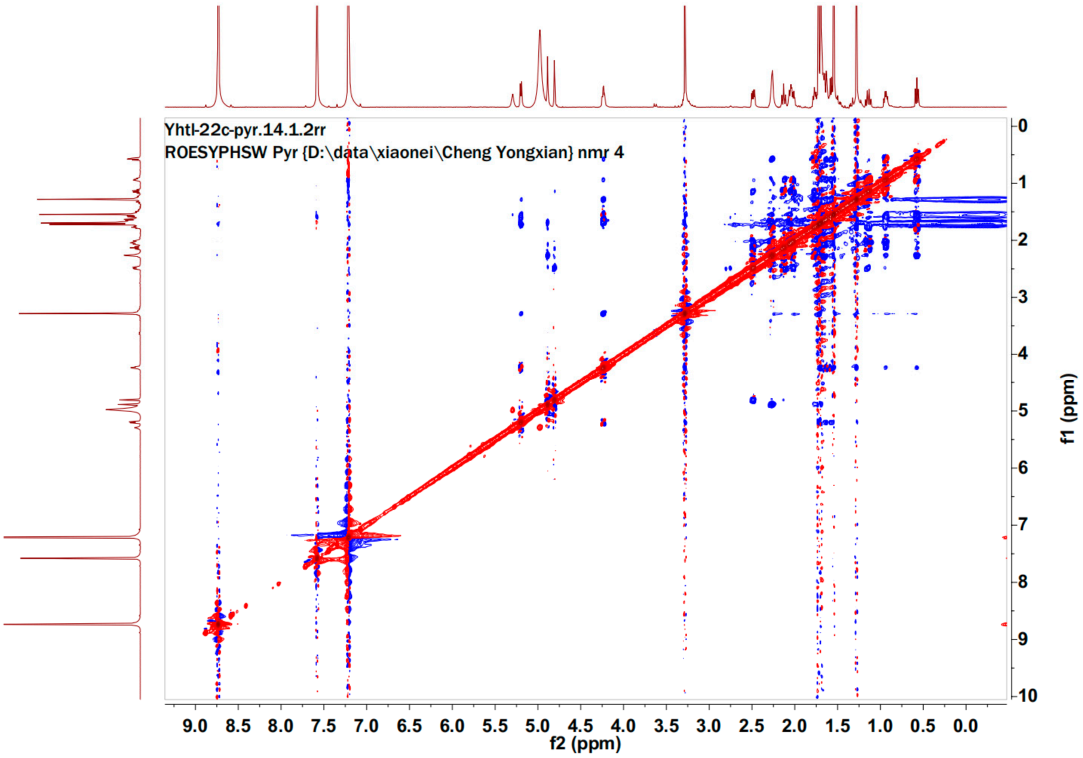

Figure S42. ROESY spectrum of **4** in Pyridine-d_5.

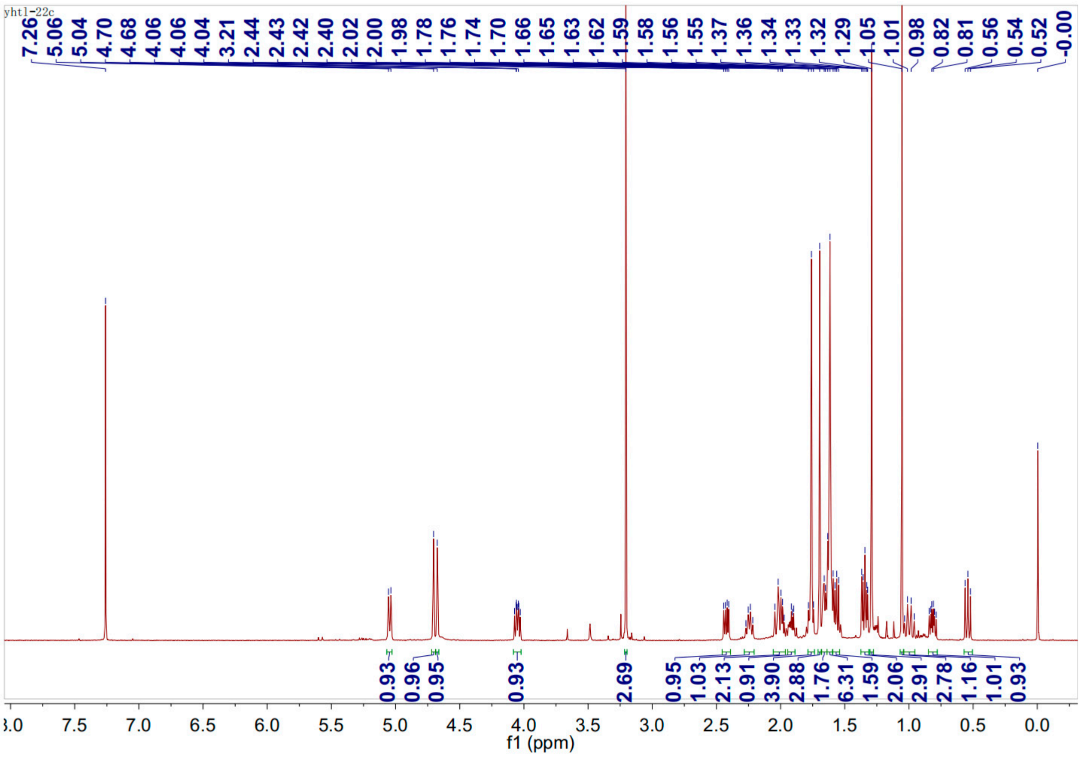

Figure S43. ^{1}H NMR spectrum of **4** in $CDCl_3$.

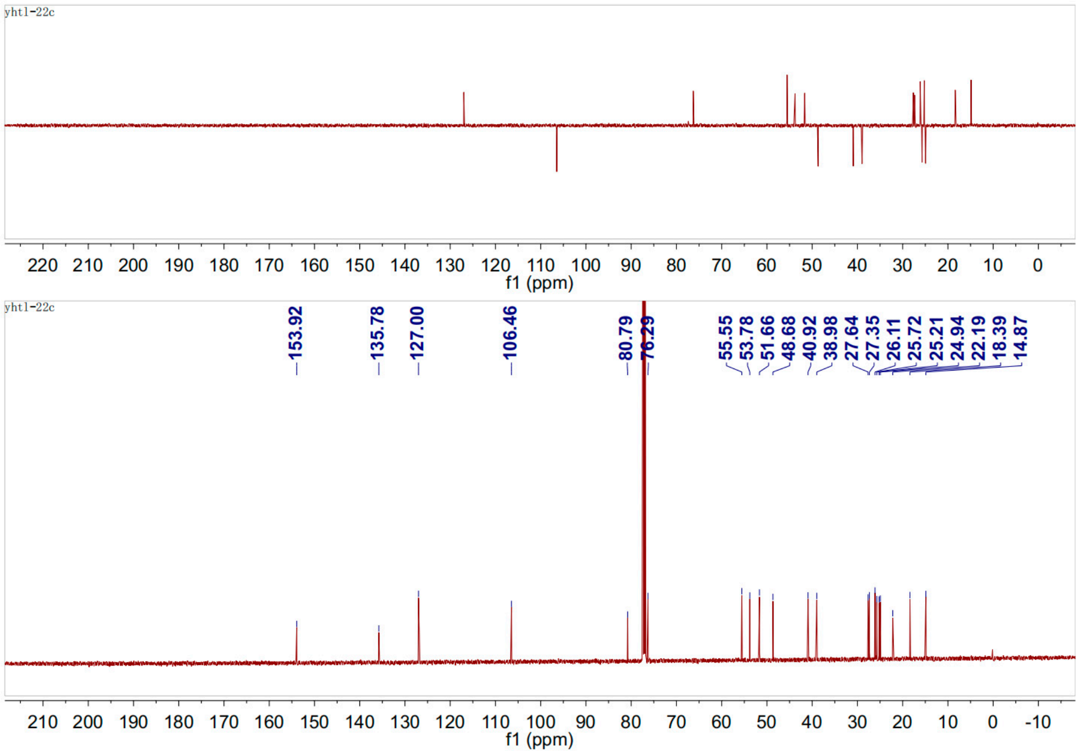

Figure S44. ^{13}C NMR and DEPT spectra of **4** in $CDCl_3$.

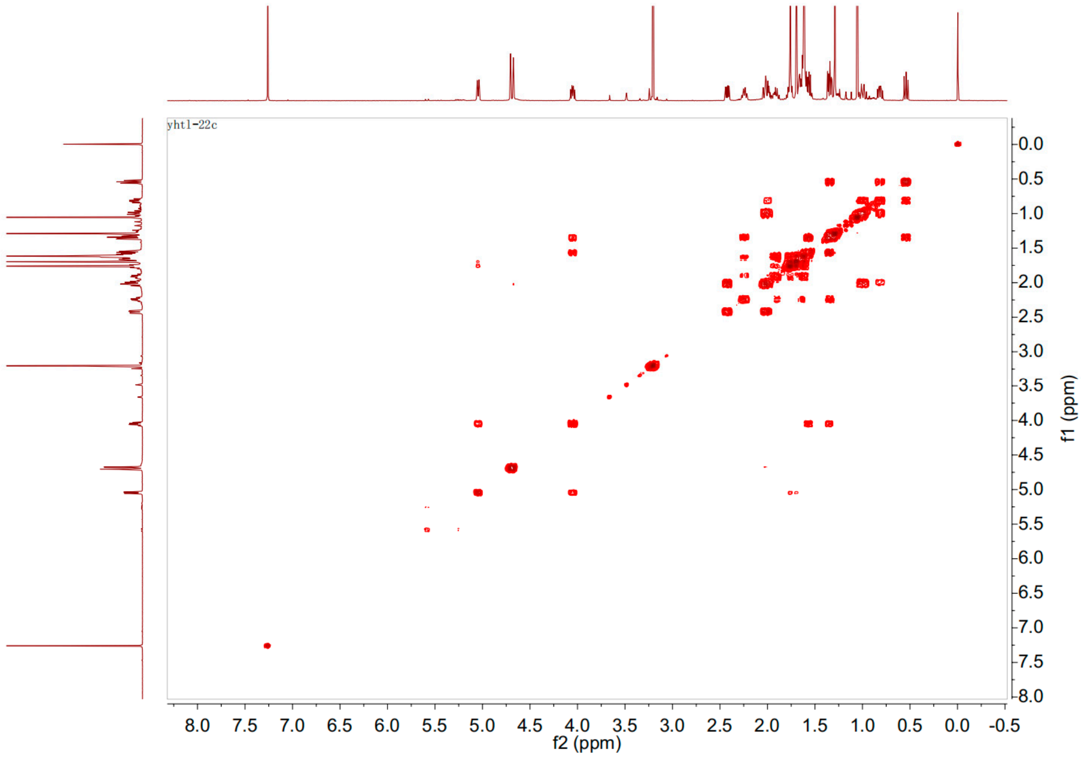

Figure S45. ^{1}H-^{1}H COSY spectrum of **4** in $CDCl_3$.

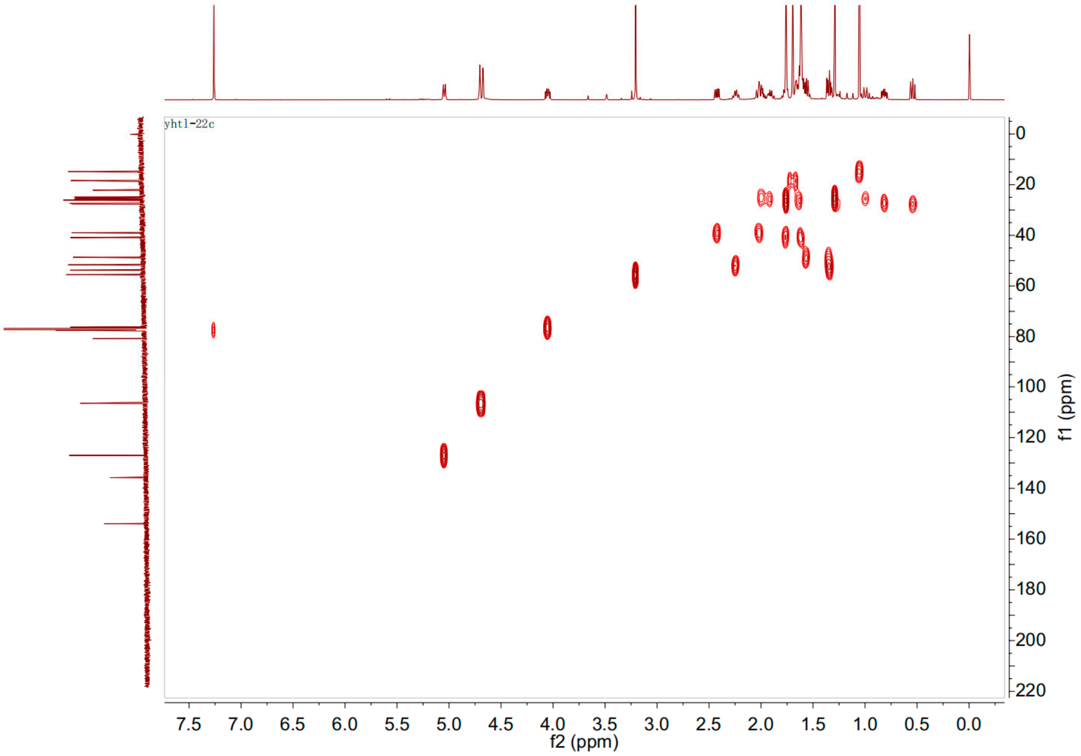

Figure S46. HSQC spectrum of **4** in $CDCl_3$.

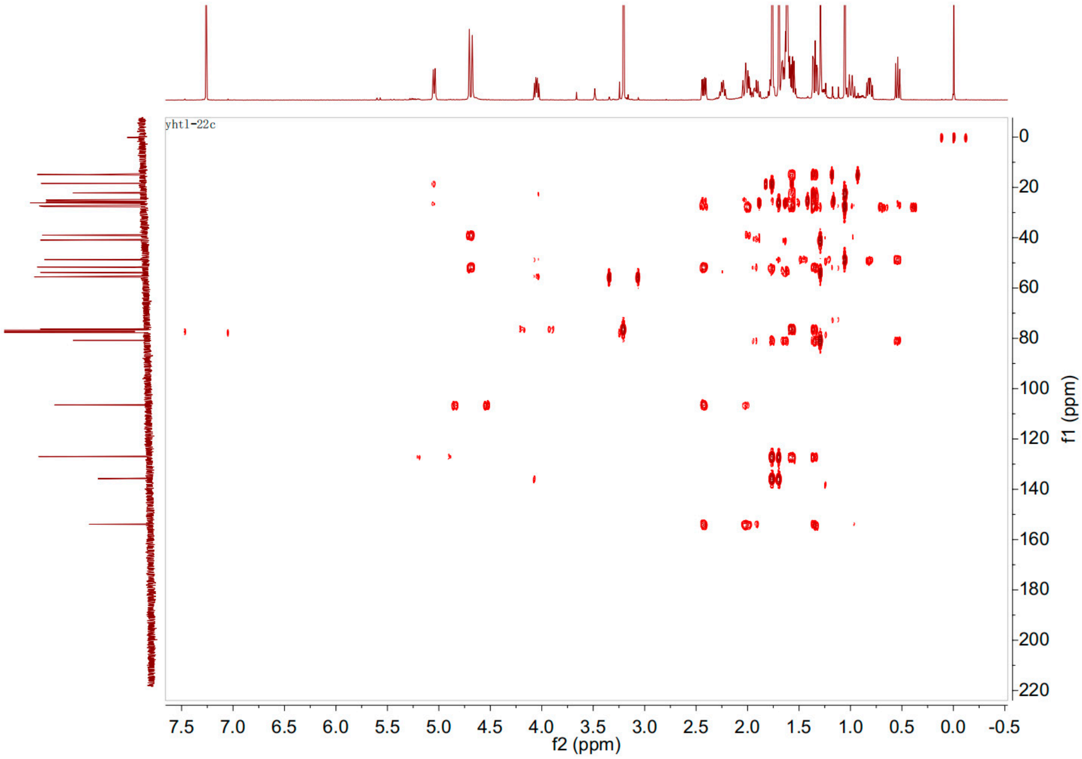

Figure S47. HMBC spectrum of **4** in $CDCl_3$.

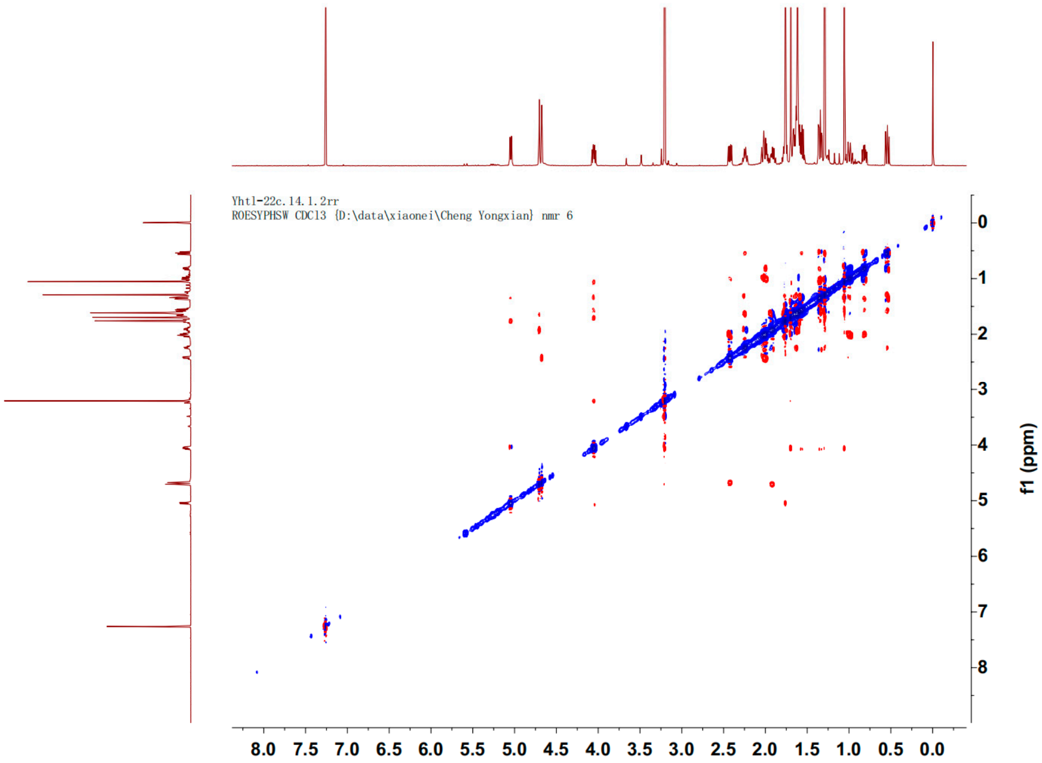

Figure S48. ROESY spectrum of **4** in $CDCl_3$.

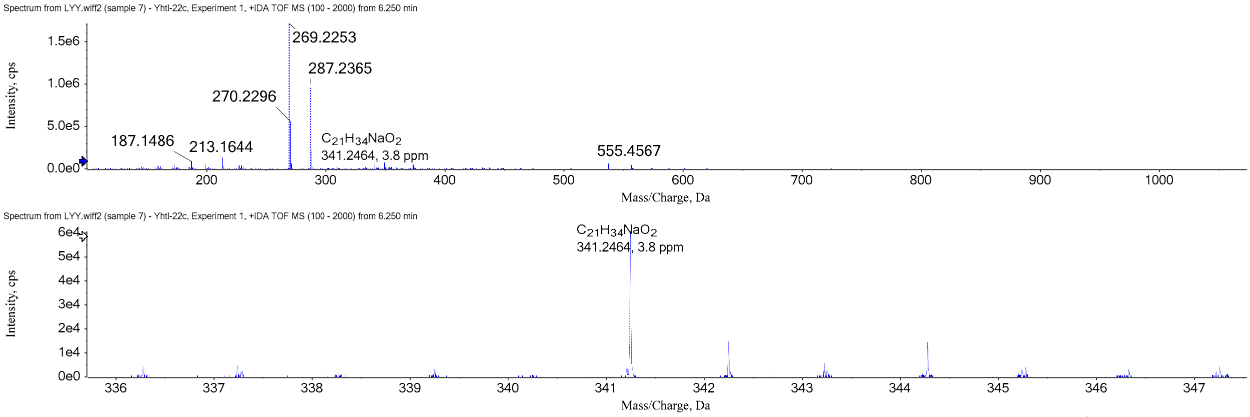

Hit	Formula	m/z	RDB	ppm	MS Rank	MSMS ppm	MSMS Rank	Found
1	C21H34O2	341.2451	5.0	3.8	1			NA/NA

Figure S49. HREIMS of **4**.

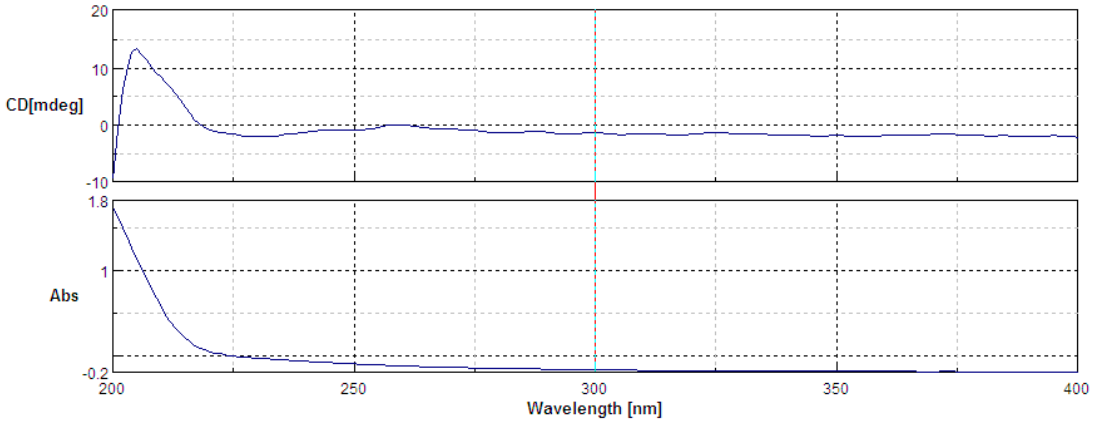

Figure S50. CD and UV spectra of **4**.

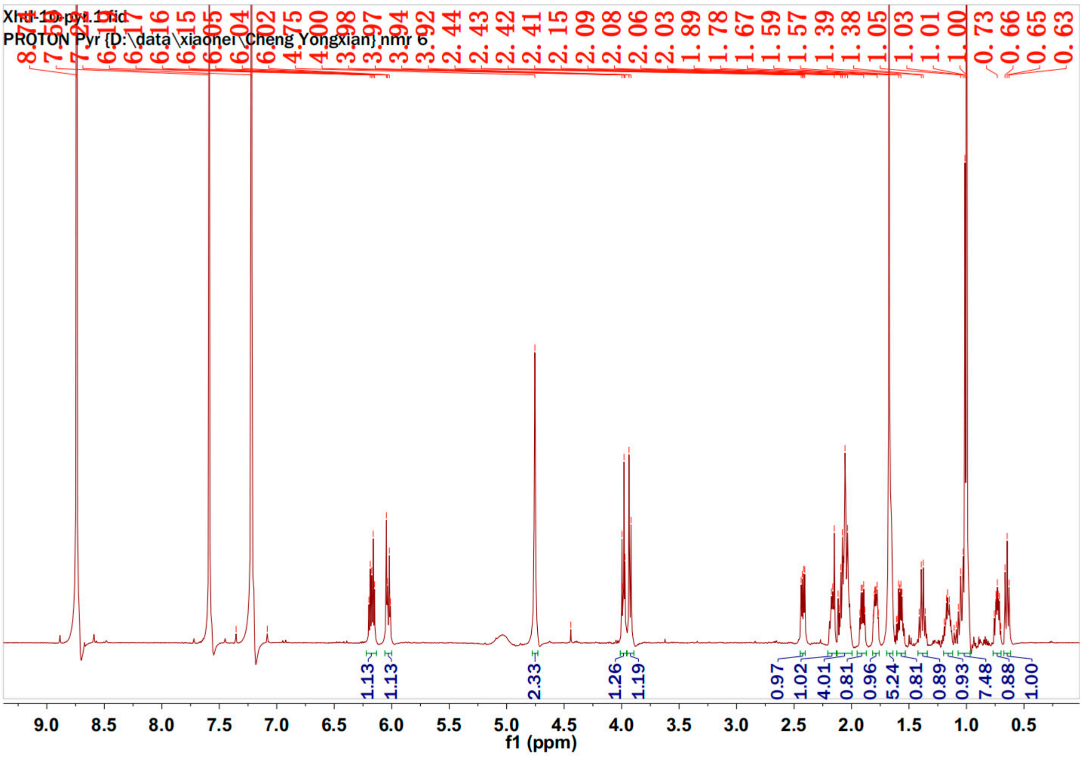

Figure S51. ^{1}H NMR spectrum of **5** in Pyridine-d_5.

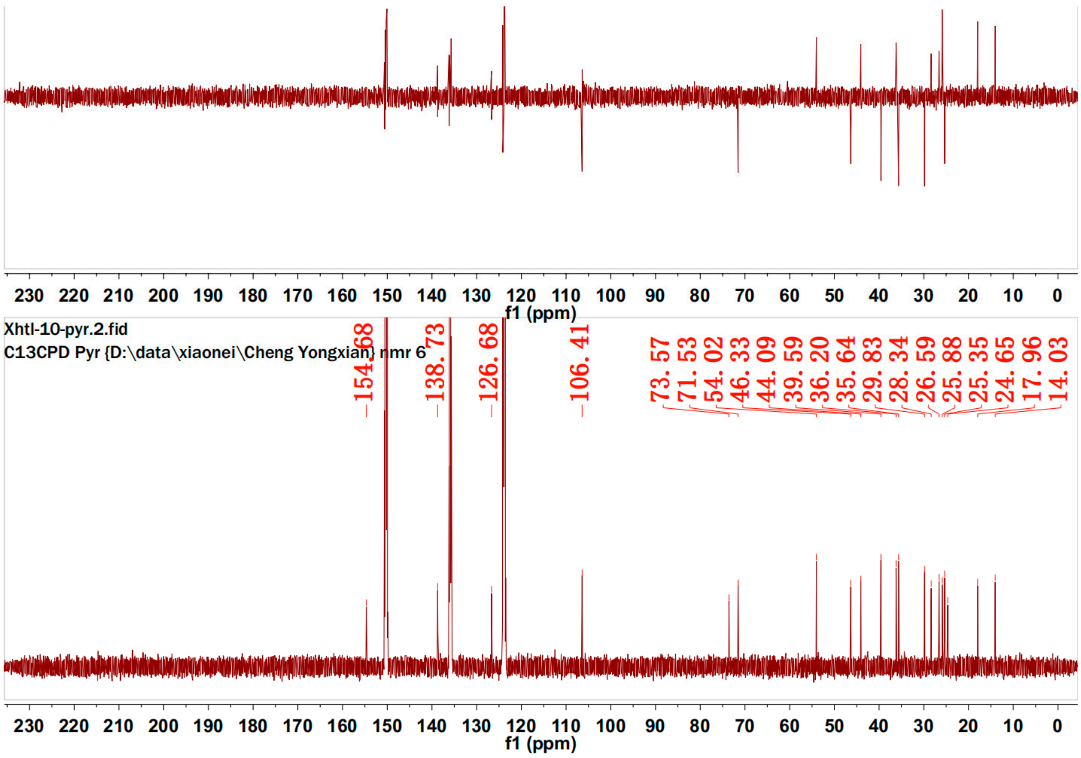

Figure S52. ^{13}C NMR and DEPT spectra of **5** in Pyridine-d_5.

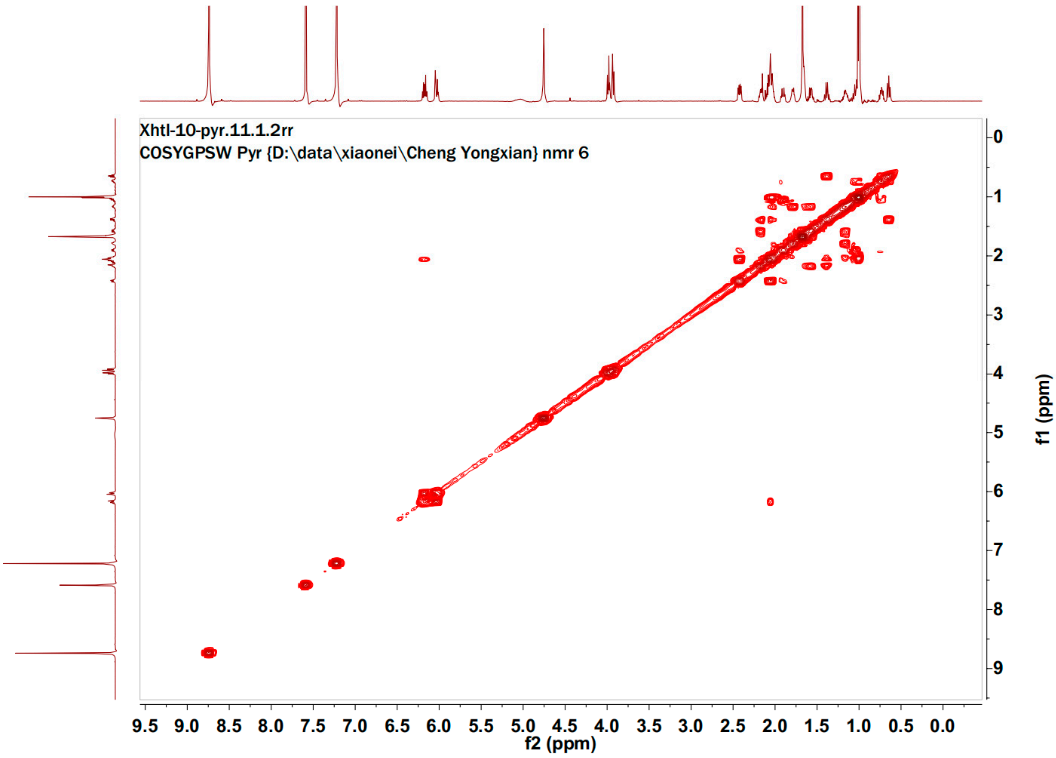

Figure S53. ^{1}H-^{1}H COSY spectrum of **5** in Pyridine-d_5.

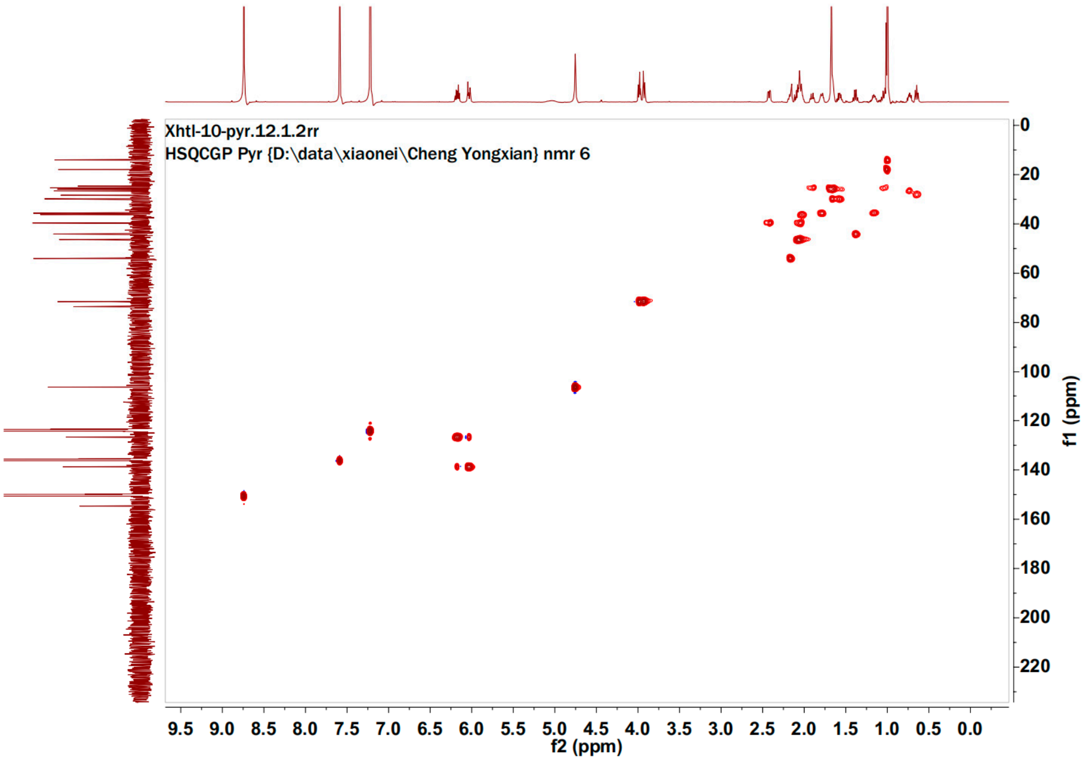

Figure S54. HSQC spectrum of **5** in Pyridine-d_5.

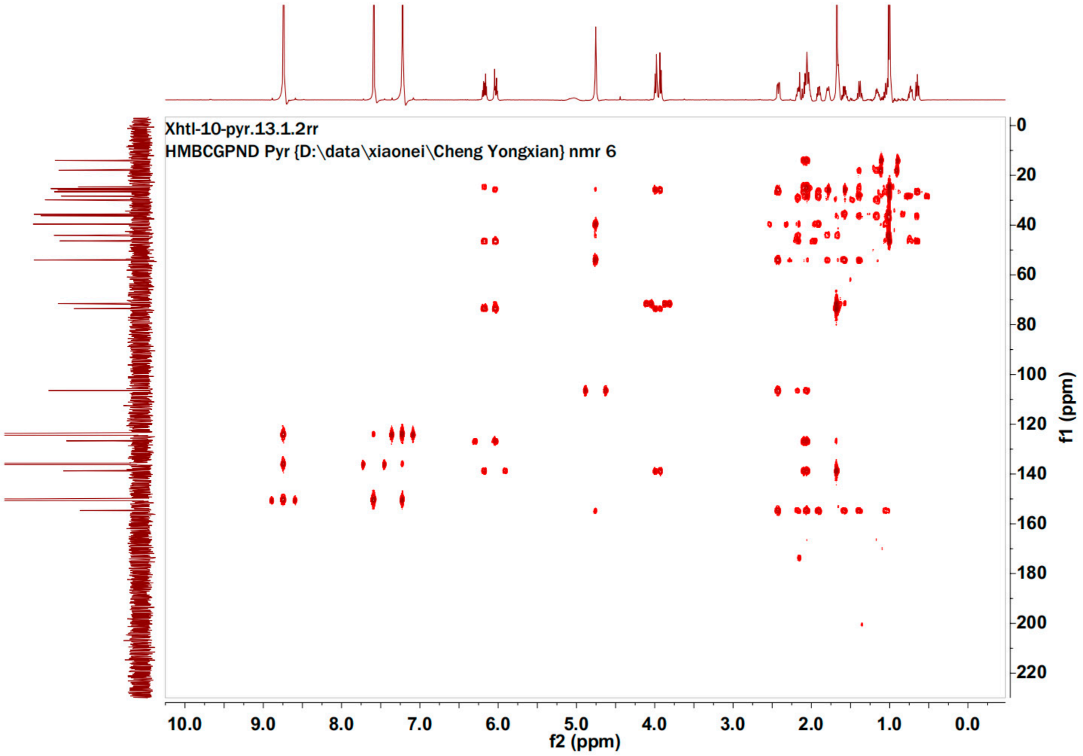

Figure S55. HMBC spectrum of **5** in Pyridine-d_5.

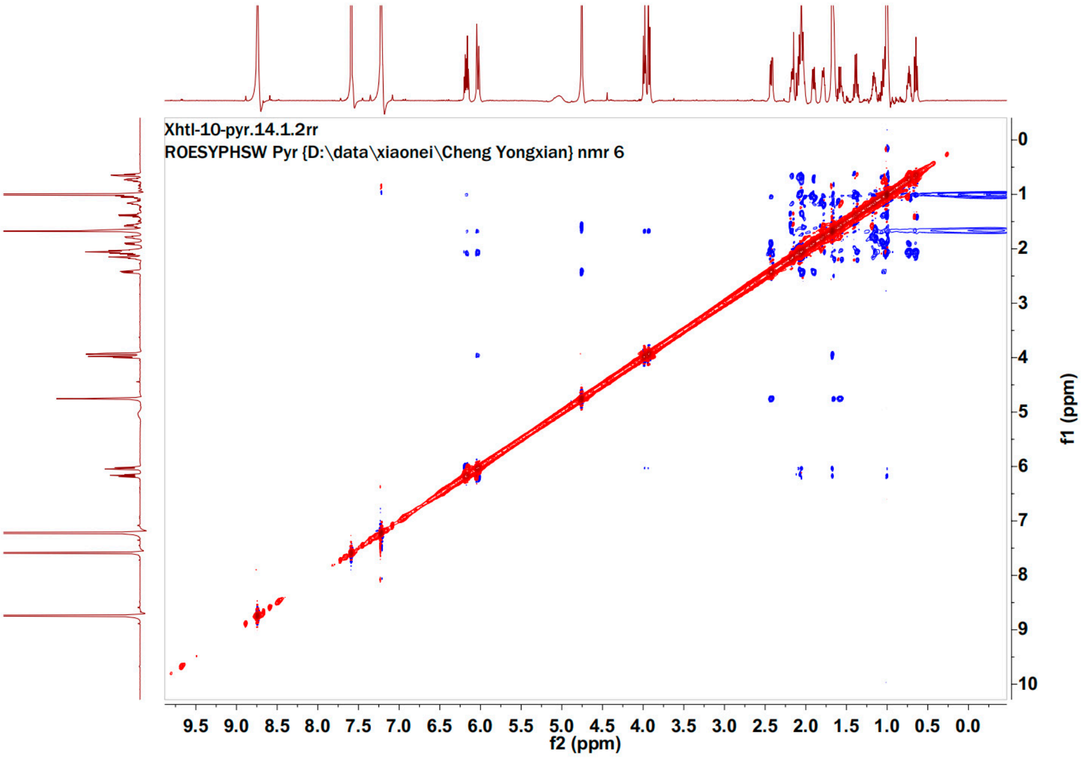

Figure S56. ROESY spectrum of **5** in Pyridine-d_5.

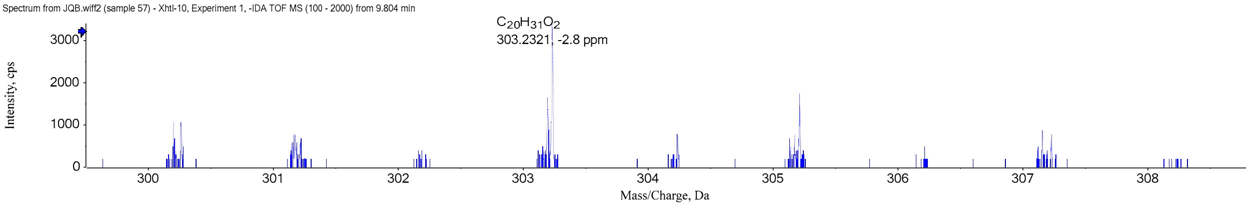

Hit	Formula	m/z	RDB	ppm	MS Rank	MSMS ppm	MSMS Rank	Found
1	C20H32O2	303.2330	5.0	-2.8	1			NA/NA

Figure S57. HREIMS of **5**.

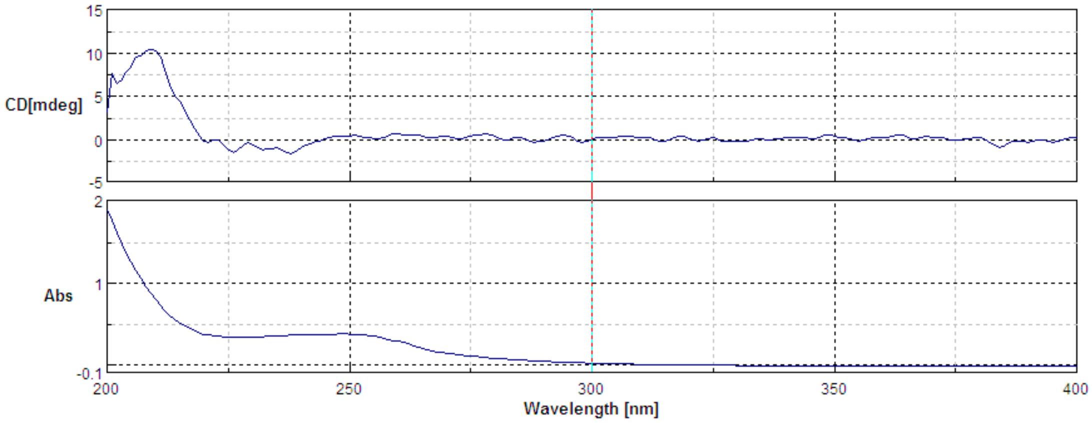

Figure S58. CD and UV spectra of **5**.

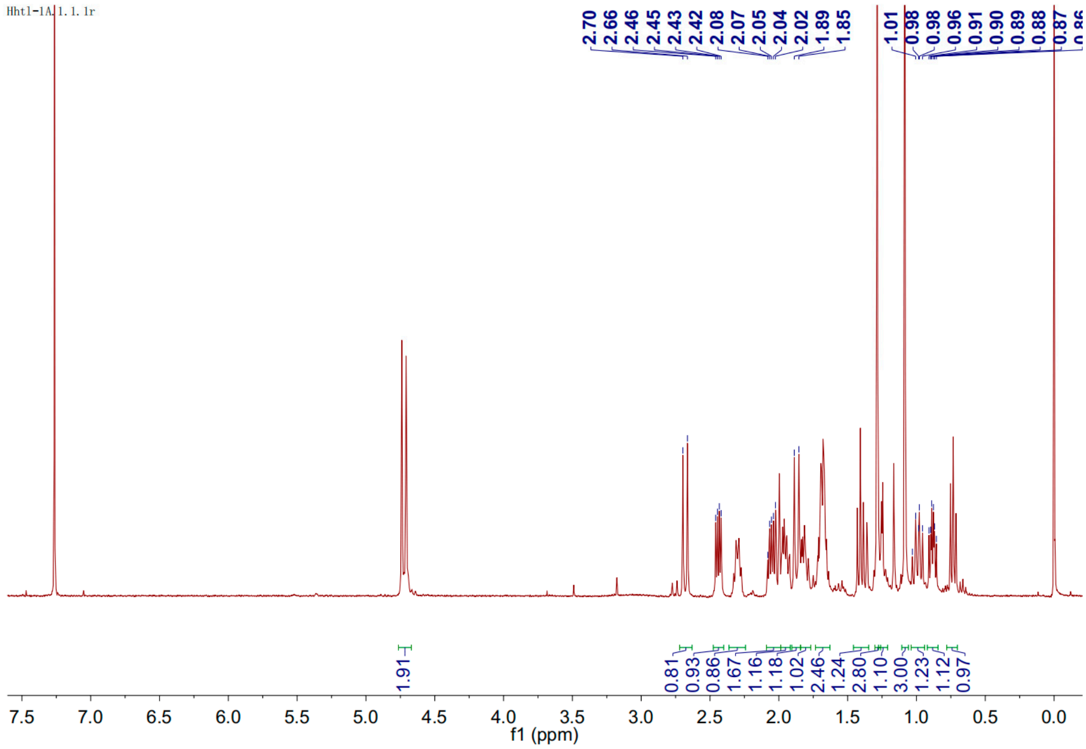

Figure S59. ^{1}H NMR spectrum of **6** in $CDCl_3$.

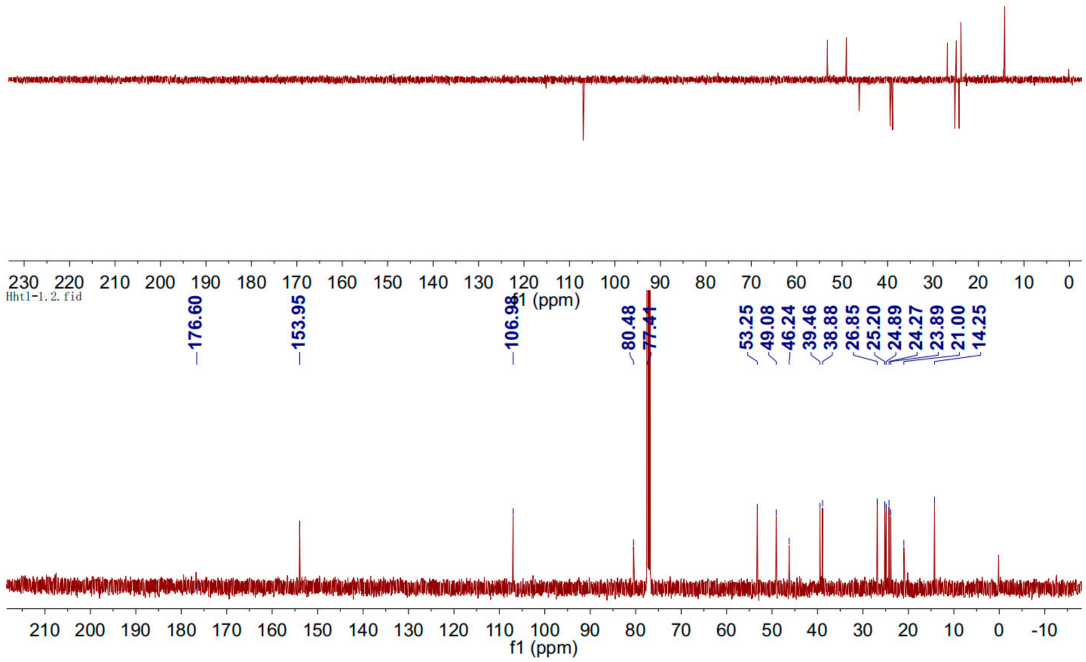

Figure S60. ^{13}C NMR and DEPT spectra of **6** in $CDCl_3$.

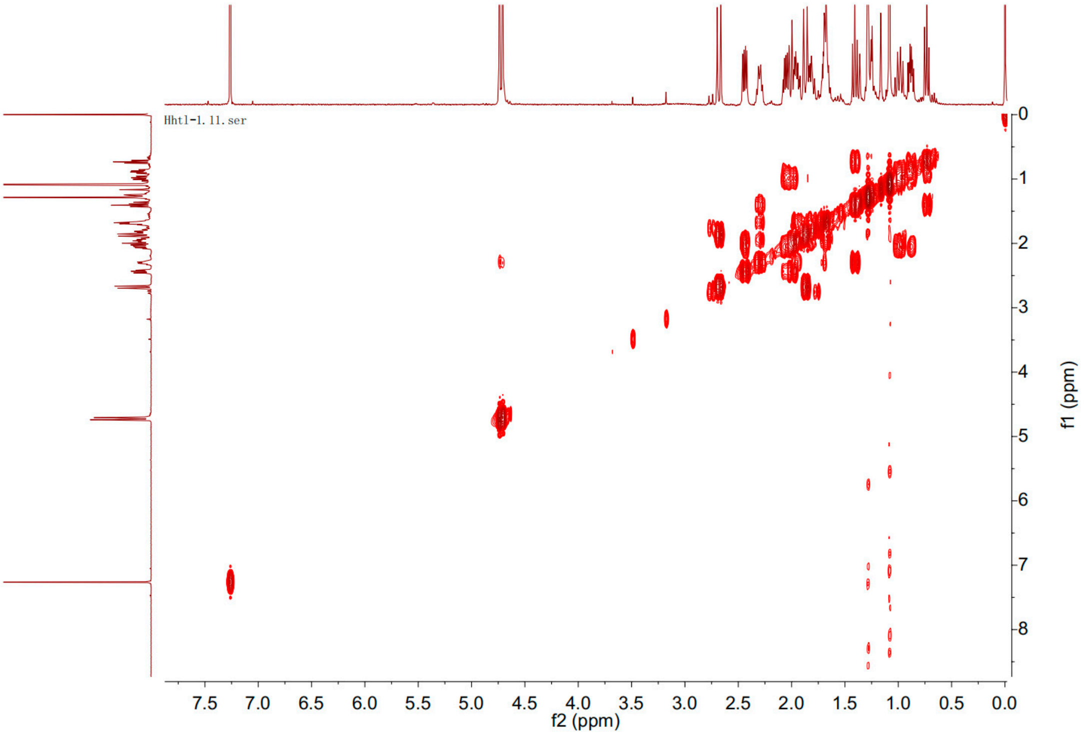

Figure S61. ^{1}H-^{1}H COSY spectrum of **6** in $CDCl_3$.

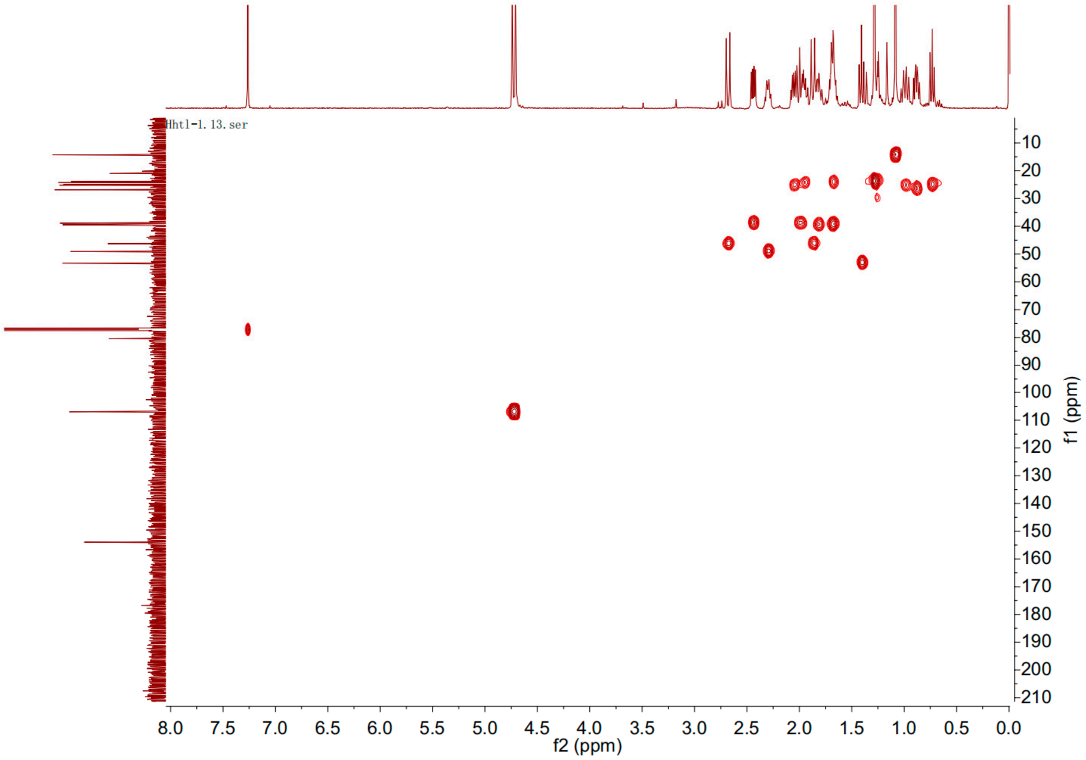

Figure S62. HSQC spectrum of **6** in $CDCl_3$.

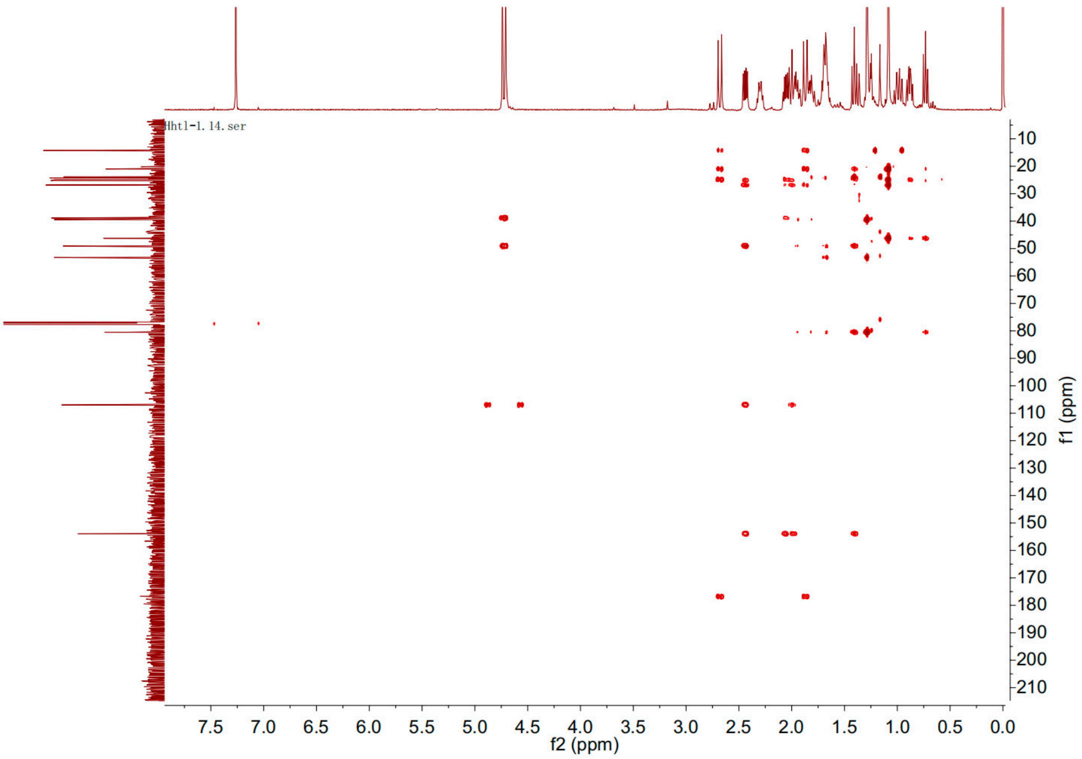

Figure S63. HMBC spectrum of **6** in $CDCl_3$.

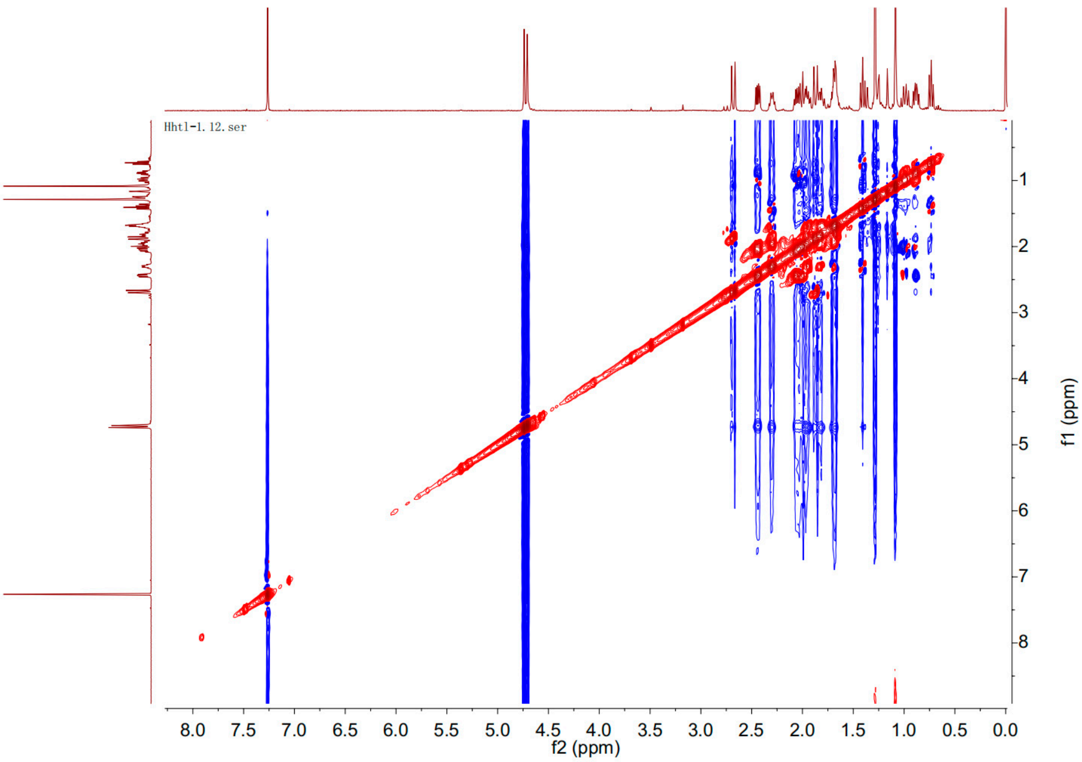

Figure S64. HMBC spectrum of **6** in $CDCl_3$.

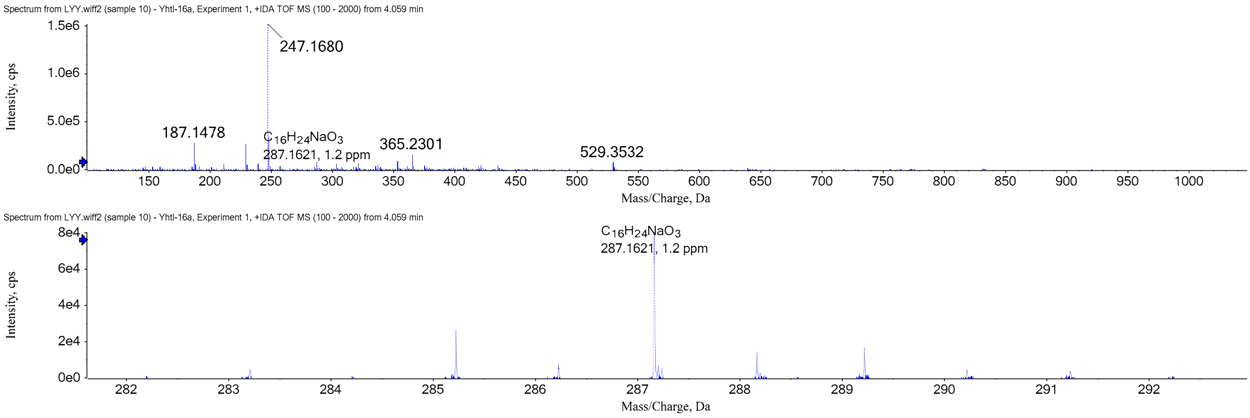

Hit	Formula	m/z	RDB	ppm	MS Rank	MSMS ppm	MSMS Rank	Found
1	C16H23NaO3	287.1618	5.0	1.2	1			NA/NA

Figure S65. HREIMS of **6**.

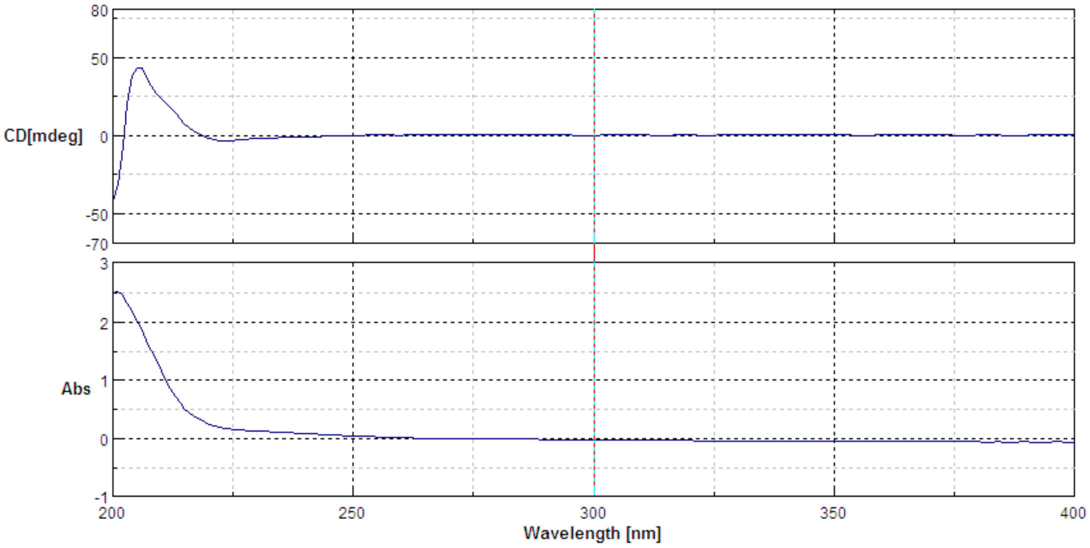

Figure S66. CD and UV spectra of **6**.

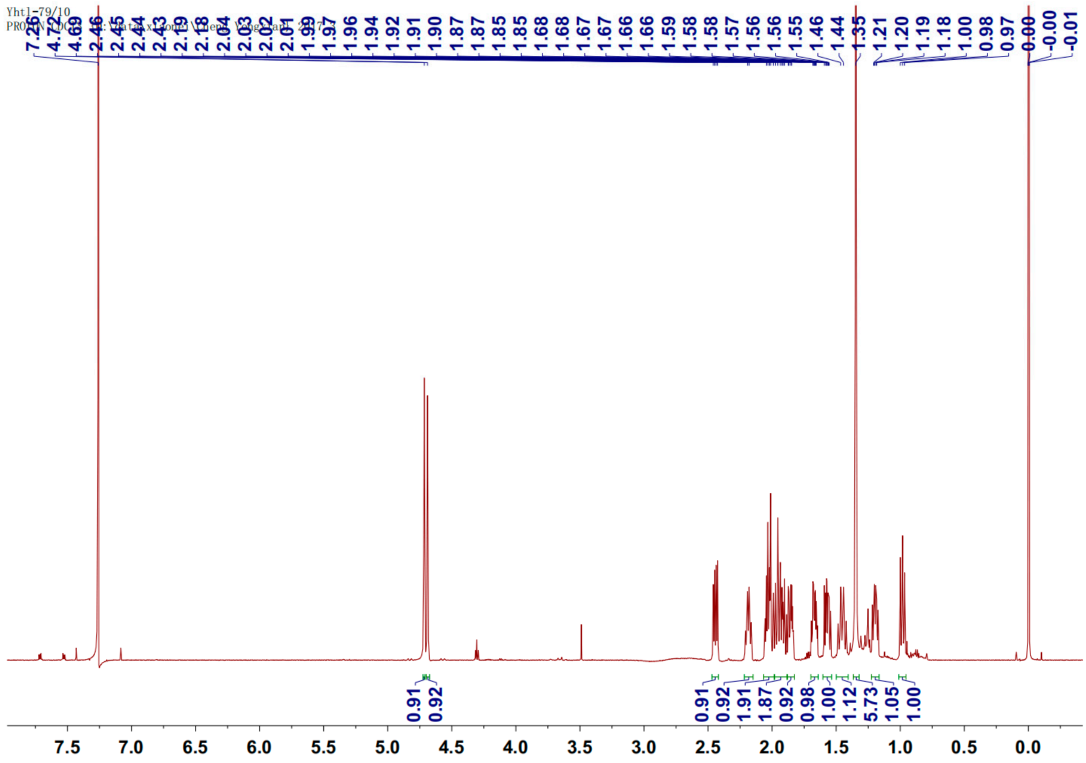

Figure S67. ^{1}H NMR spectrum of **7** in $CDCl_3$.

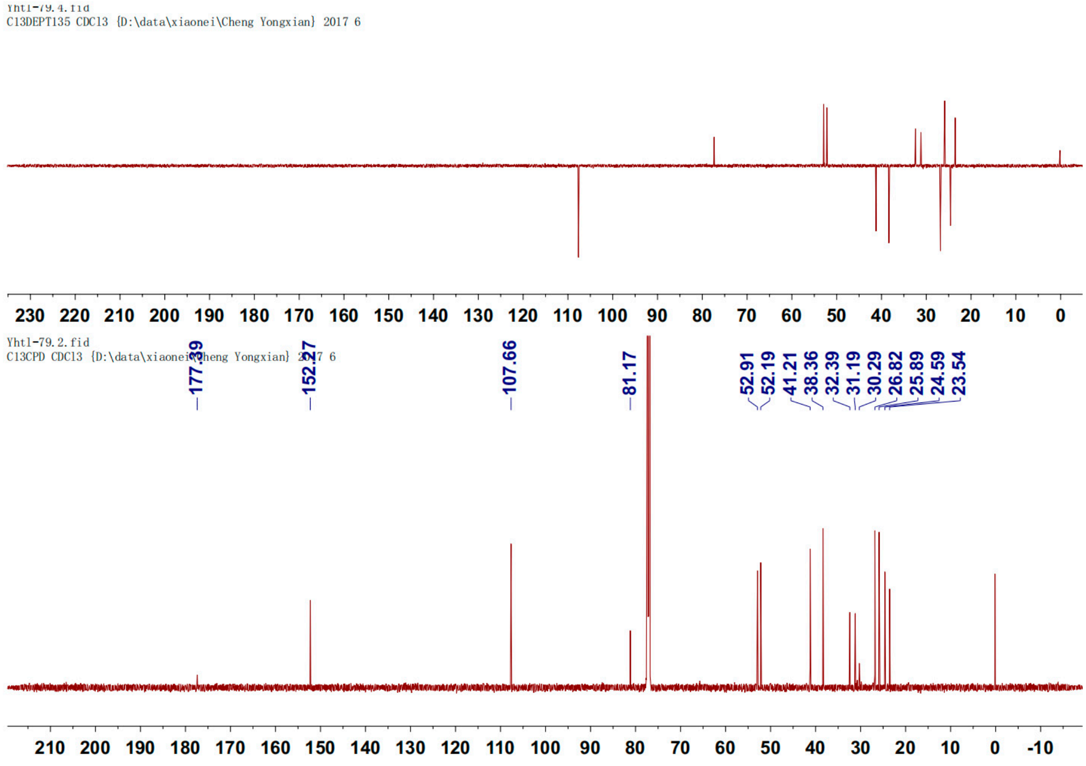

Figure S68. ^{13}C NMR and DEPT spectra of **7** in $CDCl_3$.

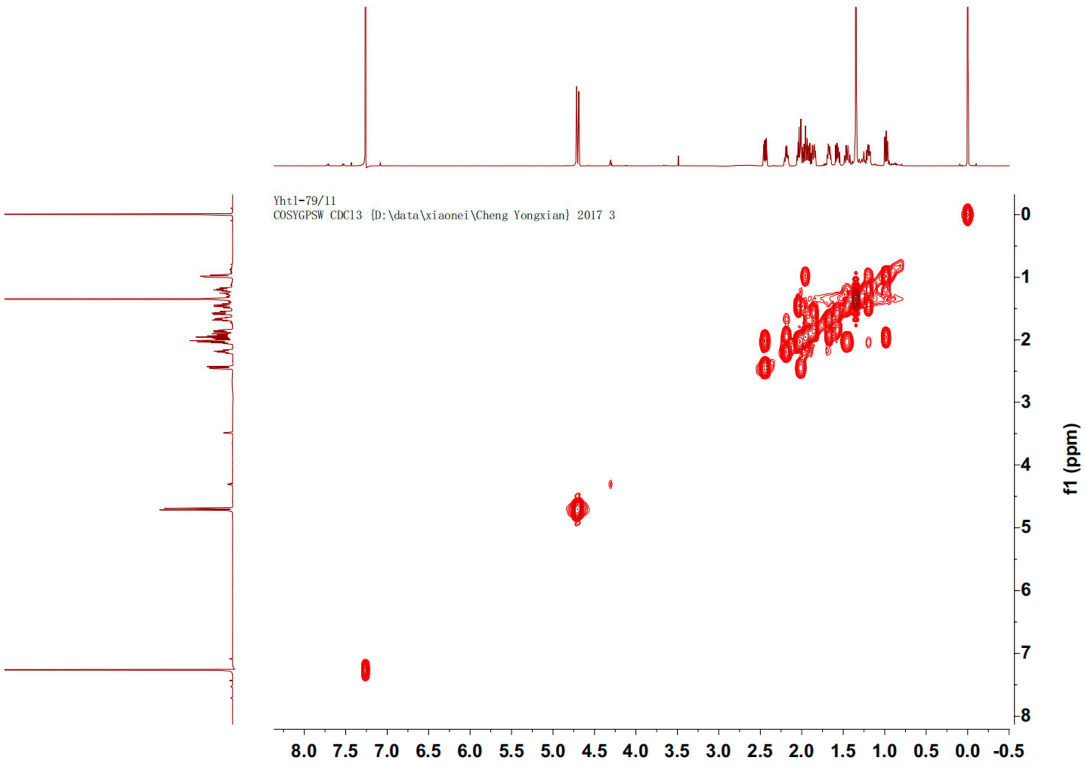

Figure S69. ^{1}H-^{1}H COSY spectrum of **7** in $CDCl_3$.

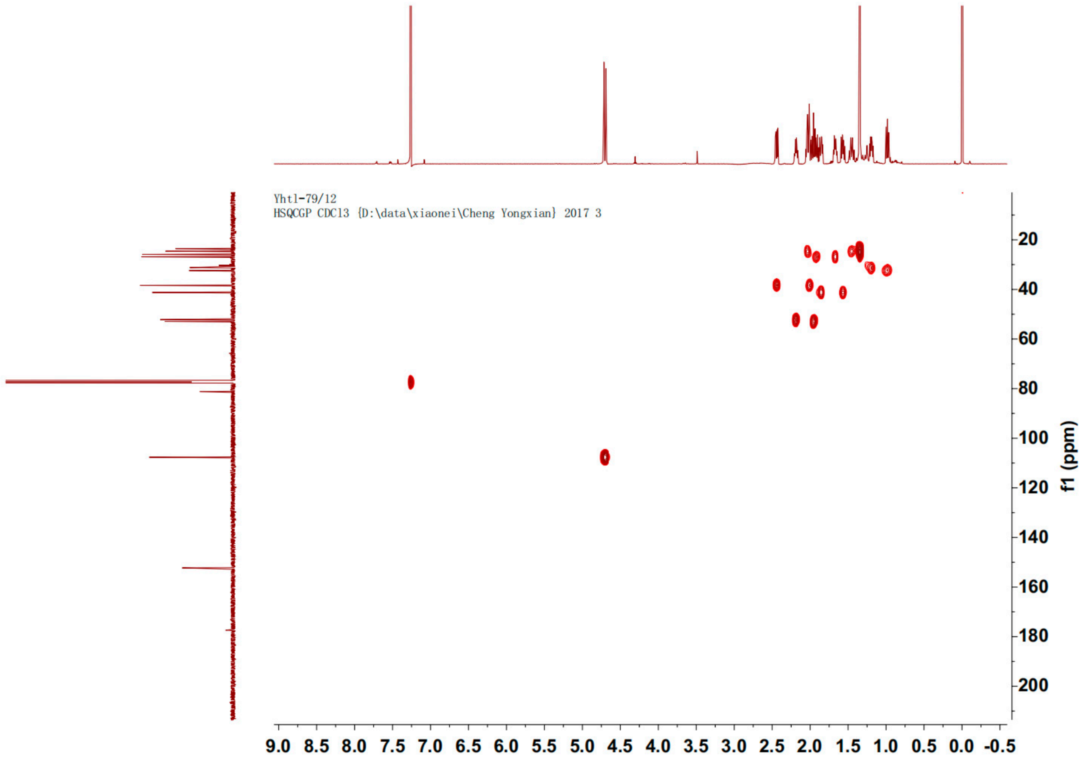

Figure S70. HSQC spectrum of **7** in $CDCl_3$.

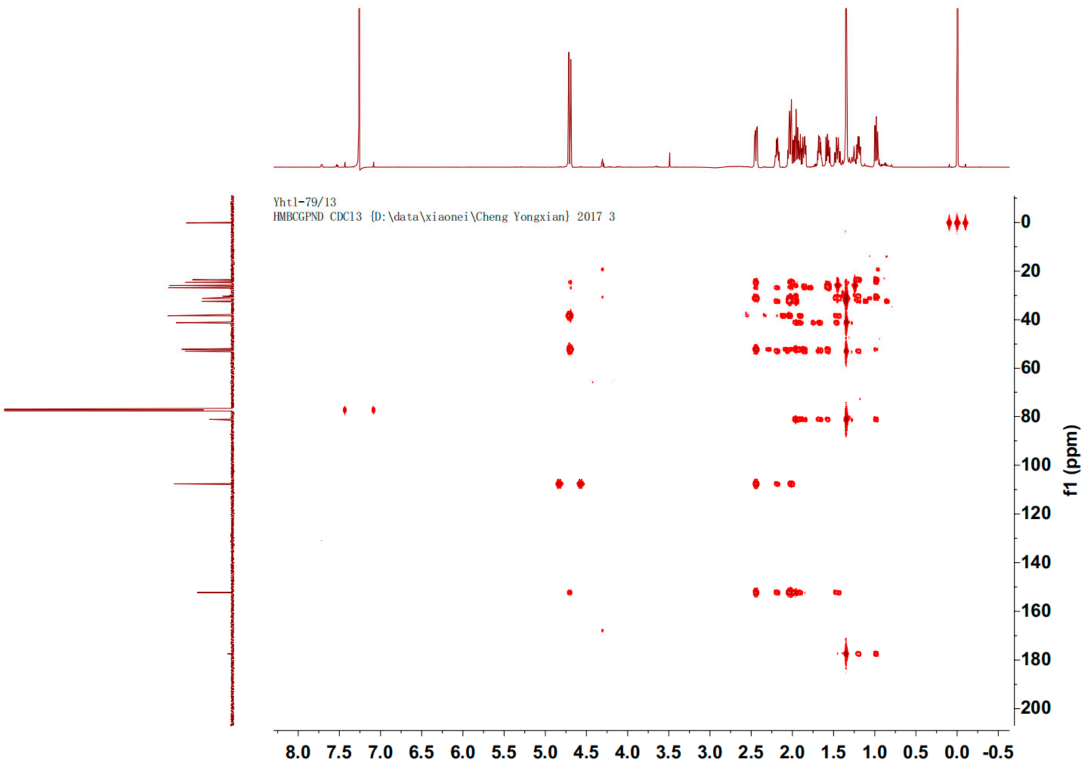

Figure S71. HMBC spectrum of **7** in CDCl3.

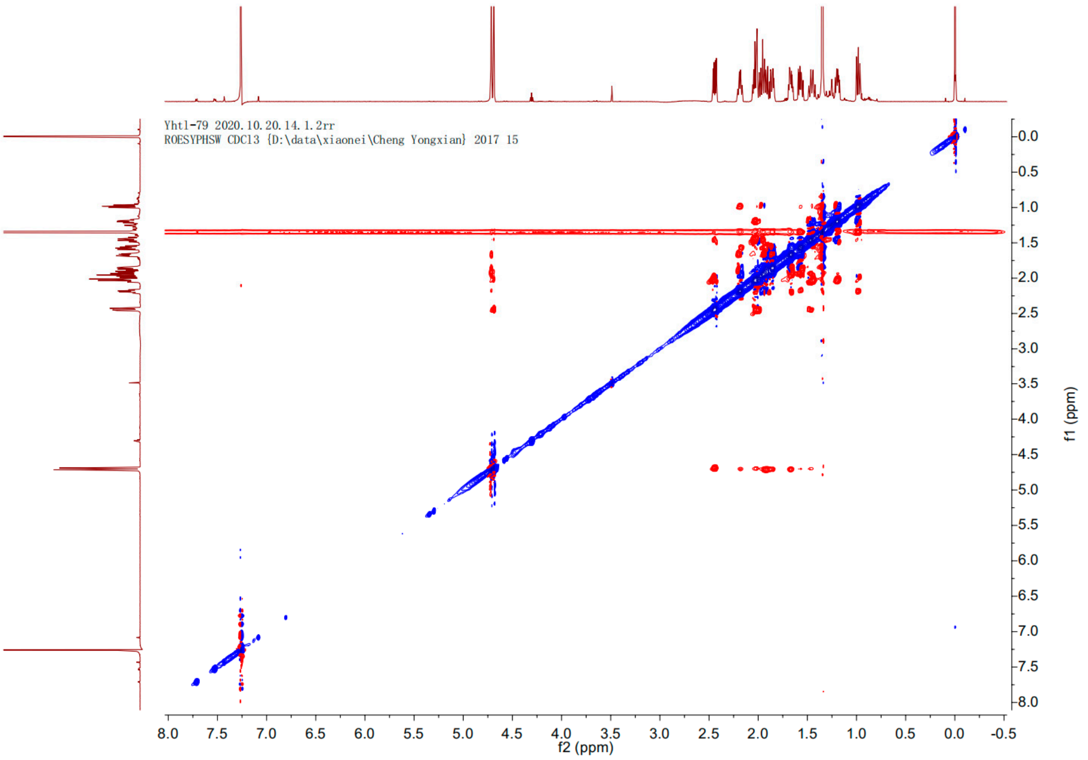

Figure S72. HMBC spectrum of **7** in CDCl3.

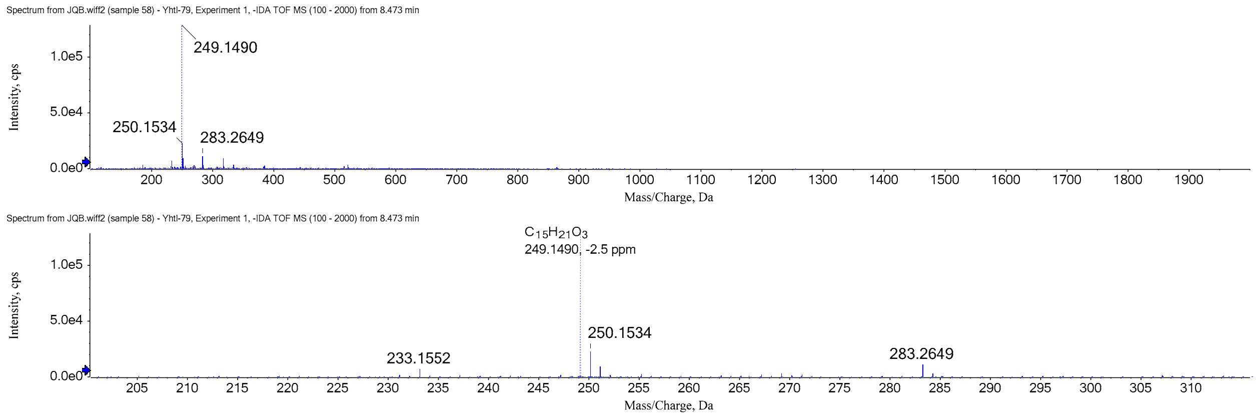

Hit	Formula	m/z	RDB	ppm	MS Rank	MSMS ppm	MSMS Rank	Found
1	C15H22O3	249.1496	5.0	-2.5	1			NA/NA

Figure S73. HREIMS of **7**.

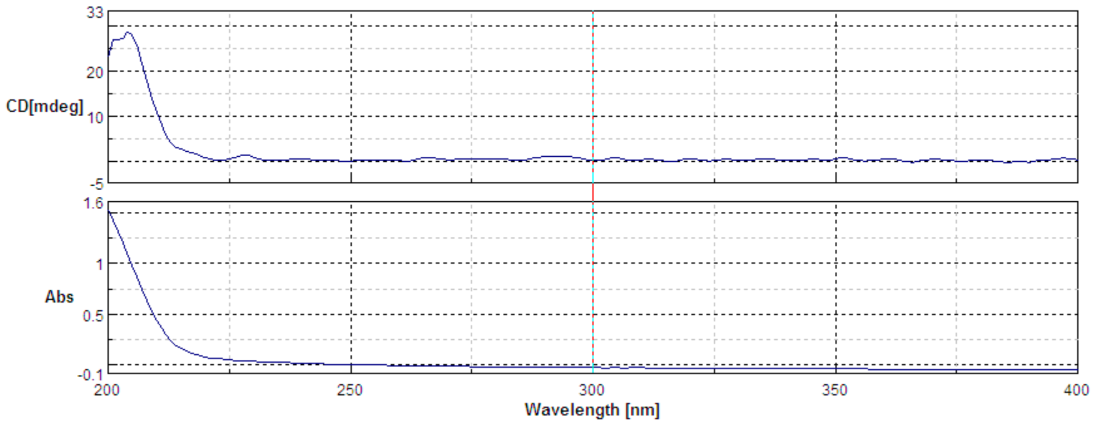

Figure S74. CD and UV spectra of **7**.

X-ray crystallographic data of 1.

Compound **1**: data for $C_{20}H_{30}O_2$ (M =302.44 g/mol): monoclinic, space group P2$_1$ (no. 4), a = 6.19090(10) Å, b = 15.92400(10) Å, c = 9.18400(10) Å, β = 104.1980(10)°, V = 877.738(18) Å^3, Z = 2, T = 100.00(10) K, μ(Cu Kα) = 0.553 mm^{-1}, $Dcalc$ = 1.144 g/cm^3, 8089 reflections measured (9.934° ≤ 2Θ ≤ 148.818°), 3454 unique (R_{int} = 0.0185, R_{sigma} = 0.0210) which were used in all calculations. The final R_1 was 0.0293 (I > 2σ(I)) and wR_2 was 0.0766 (all data).

Table 1 Crystal data and structure refinement for Madhu Babu_YHTL-91C_collect.

Identification code	Madhu Babu_YHTL-91C_collect
Empirical formula	$C_{20}H_{30}O_2$
Formula weight	302.44
Temperature/K	100.00(10)

Crystal system	monoclinic
Space group	$P2_1$
a/Å	6.19090(10)
b/Å	15.92400(10)
c/Å	9.18400(10)
α/°	90
β/°	104.1980(10)
γ/°	90
Volume/Å^3	877.738(18)
Z	2
ρ_{calc}g/cm^3	1.144
μ/mm^{-1}	0.553
F(000)	332.0
Crystal size/mm^3	0.1 × 0.09 × 0.08
Radiation	Cu Kα (λ = 1.54184)
2Θ range for data collection/°	9.934 to 148.818
Index ranges	-7 ≤ h ≤ 6, -19 ≤ k ≤ 19, -9 ≤ l ≤ 11
Reflections collected	8089
Independent reflections	3454 [R_{int} = 0.0185, R_{sigma} = 0.0210]
Data/restraints/parameters	3454/1/204
Goodness-of-fit on F^2	1.030
Final R indexes [I>=2σ (I)]	R_1 = 0.0293, wR_2 = 0.0763
Final R indexes [all data]	R_1 = 0.0296, wR_2 = 0.0766
Largest diff. peak/hole / e Å^{-3}	0.17/-0.16
Flack parameter	-0.07(7)

Table 2 Fractional Atomic Coordinates (×10^4) and Equivalent Isotropic Displacement Parameters (Å^2×10^3) for Madhu Babu_YHTL-91C_collect. U_{eq} is defined as 1/3 of of the trace of the orthogonalised U_{IJ} tensor.

Atom	*x*	*y*	*z*	U(eq)
O18	2194(2)	4198.8(8)	1656.7(14)	29.1(3)
O20	-837(2)	4253.4(8)	3717.7(13)	28.2(3)
C16	3782(3)	4571.8(11)	1381.8(19)	23.8(3)
C6	3213(3)	5541.4(10)	4352.9(18)	21.6(3)
C18	5234(3)	3436.5(10)	-65.9(18)	23.3(3)
C14	511(3)	6061.9(10)	1817.1(18)	23.8(3)
C17	5343(3)	4196.3(11)	579.4(19)	26.0(3)
C13	2824(3)	5863.5(10)	2759.7(18)	21.6(3)
C5	1380(3)	5398.8(10)	5133.6(18)	21.1(3)
C4	593(3)	4477.5(10)	5140.2(18)	23.9(3)

Table 2 Fractional Atomic Coordinates (×10^4^) and Equivalent Isotropic Displacement Parameters (Å^2^×10^3^) for Madhu Babu_YHTL-91C_collect. U_{eq} is defined as 1/3 of of the trace of the orthogonalised U_{IJ} tensor.

Atom	*x*	*y*	*z*	U(eq)
C8	2845(3)	7163.6(11)	4627(2)	26.9(3)
C1	1952(3)	5675.5(11)	6805.3(19)	27.0(4)
C20	7057(3)	3159.8(12)	-773(2)	30.7(4)
C10	1598(3)	6614.8(11)	6918.1(19)	27.7(4)
C3	-770(3)	4514.0(13)	6317(2)	30.8(4)
C9	3174(3)	7180.4(12)	6343(2)	30.3(4)
C15	4329(3)	5475.1(11)	1873(2)	25.4(3)
C7	3942(3)	6420.2(10)	4079.4(19)	24.1(3)
C12	-47(4)	6954.4(13)	7417(2)	35.1(4)
C19	3421(3)	2807.6(11)	-150(2)	32.1(4)
C11	2492(3)	3843.4(11)	5574(2)	29.7(4)
C2	602(4)	5074.2(13)	7565(2)	36.0(4)

Table 3 Anisotropic Displacement Parameters (Å^2^×10^3^) for Madhu Babu_YHTL-91C_collect. The Anisotropic displacement factor exponent takes the form: $-2\pi^2[h^2a^{*2}U_{11}+2hka^*b^*U_{12}+\ldots]$.

Atom	U_{11}	U_{22}	U_{33}	U_{23}	U_{13}	U_{12}
O18	30.6(6)	26.0(6)	35.3(6)	-6.7(5)	17.1(5)	-6.4(5)
O20	28.7(6)	30.7(6)	24.2(6)	0.2(5)	4.5(5)	-7.1(5)
C16	24.3(8)	25.3(8)	23.0(7)	-1.7(6)	7.9(6)	-2.1(6)
C6	19.2(7)	20.2(7)	24.9(8)	-1.7(6)	4.6(6)	1.1(6)
C18	24.9(8)	26.8(8)	18.0(7)	1.1(6)	4.9(6)	3.8(6)
C14	25.9(8)	22.6(8)	23.5(7)	2.9(6)	7.4(6)	-0.6(6)
C17	24.5(8)	28.5(8)	26.8(8)	-2.7(7)	9.8(6)	-2.8(7)
C13	22.3(7)	19.2(7)	24.8(8)	-1.9(6)	8.8(6)	-2.6(6)
C5	19.7(7)	22.1(8)	20.5(7)	1.3(6)	3.1(6)	3.0(6)
C4	23.6(7)	24.2(8)	22.9(8)	2.2(6)	3.9(6)	-0.6(6)
C8	31.5(8)	19.6(7)	30.6(8)	-3.0(7)	9.6(7)	-1.5(7)
C1	30.1(8)	28.6(9)	21.4(8)	-0.6(7)	4.5(6)	3.0(7)
C20	32.0(9)	33.2(10)	29.5(9)	-1.4(7)	12.5(7)	6.5(7)
C10	31.3(9)	29.2(9)	21.0(7)	-2.7(6)	2.9(7)	3.2(7)
C3	28.0(8)	37.5(10)	28.3(8)	5.9(7)	9.7(7)	-1.5(7)
C9	33.0(9)	27.2(8)	29.8(9)	-9.3(7)	6.1(7)	-0.9(7)
C15	24.8(8)	24.1(8)	30.2(8)	-3.2(6)	12.5(7)	-3.6(6)
C7	21.8(7)	21.8(8)	29.0(8)	-3.8(6)	6.6(7)	-3.0(6)
C12	41.1(10)	34.4(10)	32.0(10)	0.3(8)	13.3(8)	8.2(8)

Table 3 Anisotropic Displacement Parameters (Å^2×10^3) for Madhu Babu_YHTL-91C_collect. The Anisotropic displacement factor exponent takes the form: $-2\pi^2[h^2a^{*2}U_{11}+2hka^*b^*U_{12}+\ldots]$.

Atom	U_{11}	U_{22}	U_{33}	U_{23}	U_{13}	U_{12}
C19	36.6(9)	25.4(9)	37.1(10)	-7.3(7)	14.0(8)	-2.4(7)
C11	31.9(9)	22.9(8)	33.4(9)	4.8(7)	6.5(7)	3.6(7)
C2	45.3(11)	36.7(10)	28.2(9)	3.3(8)	13.4(8)	2.4(8)

Table 4 Bond Lengths for Madhu Babu_YHTL-91C_collect.

Atom	Atom	Length/Å	Atom	Atom	Length/Å
O18	C16	1.227(2)	C13	C7	1.524(2)
O20	C4	1.4320(19)	C5	C4	1.546(2)
C16	C17	1.477(2)	C5	C1	1.552(2)
C16	C15	1.521(2)	C4	C3	1.526(2)
C6	C13	1.513(2)	C4	C11	1.527(2)
C6	C5	1.501(2)	C8	C9	1.539(2)
C6	C7	1.510(2)	C8	C7	1.511(2)
C18	C17	1.342(2)	C1	C10	1.519(2)
C18	C20	1.499(2)	C1	C2	1.545(3)
C18	C19	1.492(2)	C10	C9	1.515(3)
C14	C13	1.514(2)	C10	C12	1.330(3)
C13	C15	1.512(2)	C3	C2	1.534(3)

Table 5 Bond Angles for Madhu Babu_YHTL-91C_collect.

Atom	Atom	Atom	Angle/°	Atom	Atom	Atom	Angle/°
O18	C16	C17	124.17(16)	O20	C4	C3	109.27(13)
O18	C16	C15	121.91(15)	O20	C4	C11	109.72(14)
C17	C16	C15	113.91(14)	C3	C4	C5	101.30(14)
C5	C6	C13	123.60(13)	C3	C4	C11	111.39(14)
C5	C6	C7	120.76(14)	C11	C4	C5	113.91(13)
C7	C6	C13	60.55(11)	C7	C8	C9	113.38(15)
C17	C18	C20	119.66(16)	C10	C1	C5	110.20(14)
C17	C18	C19	125.70(16)	C10	C1	C2	118.43(15)
C19	C18	C20	114.64(15)	C2	C1	C5	104.65(14)
C18	C17	C16	128.12(16)	C9	C10	C1	116.44(15)
C6	C13	C14	121.86(13)	C12	C10	C1	124.04(18)
C6	C13	C7	59.64(11)	C12	C10	C9	119.44(17)
C14	C13	C7	120.99(14)	C4	C3	C2	104.50(15)
C15	C13	C6	114.15(14)	C10	C9	C8	114.34(14)
C15	C13	C14	113.51(14)	C13	C15	C16	115.13(13)

Table 5 Bond Angles for Madhu Babu_YHTL-91C_collect.

Atom	Atom	Atom	Angle/°	Atom	Atom	Atom	Angle/°
C15	C13	C7	116.51(14)	C6	C7	C13	59.82(10)
C6	C5	C4	114.92(13)	C6	C7	C8	119.58(14)
C6	C5	C1	114.42(14)	C8	C7	C13	124.51(14)
C4	C5	C1	105.14(13)	C3	C2	C1	106.53(15)
O20	C4	C5	110.96(13)				

Table 6 Torsion Angles for Madhu Babu_YHTL-91C_collect.

A	B	C	D	Angle/°	A	B	C	D	Angle/°
O18	C16	C17	C18	5.2(3)	C4	C5	C1	C10	-151.06(14)
O18	C16	C15	C13	1.9(2)	C4	C5	C1	C2	-22.71(17)
O20	C4	C3	C2	-158.29(14)	C4	C3	C2	C1	27.78(19)
C6	C13	C15	C16	73.36(18)	C1	C5	C4	O20	155.41(13)
C6	C13	C7	C8	-107.04(18)	C1	C5	C4	C3	39.51(15)
C6	C5	C4	O20	-77.87(17)	C1	C5	C4	C11	-80.18(16)
C6	C5	C4	C3	166.23(14)	C1	C10	C9	C8	69.5(2)
C6	C5	C4	C11	46.54(19)	C20	C18	C17	C16	-177.39(16)
C6	C5	C1	C10	81.92(17)	C10	C1	C2	C3	120.29(17)
C6	C5	C1	C2	-149.73(15)	C9	C8	C7	C6	61.6(2)
C14	C13	C15	C16	-72.32(19)	C9	C8	C7	C13	133.42(17)
C14	C13	C7	C6	111.21(16)	C15	C16	C17	C18	-175.70(17)
C14	C13	C7	C8	4.2(2)	C15	C13	C7	C6	-103.75(16)
C17	C16	C15	C13	-177.30(14)	C15	C13	C7	C8	149.21(16)
C13	C6	C5	C4	98.88(18)	C7	C6	C13	C14	-109.79(17)
C13	C6	C5	C1	-139.30(15)	C7	C6	C13	C15	107.71(15)
C13	C6	C7	C8	115.06(17)	C7	C6	C5	C4	171.93(14)
C5	C6	C13	C14	-0.5(2)	C7	C6	C5	C1	-66.25(19)
C5	C6	C13	C15	-143.02(15)	C7	C13	C15	C16	140.07(15)
C5	C6	C13	C7	109.27(18)	C7	C8	C9	C10	-80.87(19)
C5	C6	C7	C13	-113.79(16)	C12	C10	C9	C8	-107.40(19)
C5	C6	C7	C8	1.3(2)	C19	C18	C17	C16	2.1(3)
C5	C4	C3	C2	-41.14(17)	C11	C4	C3	C2	80.33(18)
C5	C1	C10	C9	-67.65(19)	C2	C1	C10	C9	171.98(15)
C5	C1	C10	C12	109.11(19)	C2	C1	C10	C12	-11.3(3)
C5	C1	C2	C3	-2.90(19)					

Table 7 Hydrogen Atom Coordinates (Å×10^4) and Isotropic Displacement Parameters (Å^2×10^3) for Madhu Babu_YHTL-91C_collect.

Atom	*x*	*y*	*z*	U(eq)

Table 7 Hydrogen Atom Coordinates (Å×10⁴) and Isotropic Displacement Parameters (Å²×10³) for Madhu Babu_YHTL-91C_collect.

Atom	*x*	*y*	*z*	U(eq)
H20	-119	4250	3077	42
H6	4443	5139	4633	26
H14A	-184	5555	1363	36
H14B	614	6457	1047	36
H14C	-365	6300	2444	36
H17	6551	4528	513	31
H5	95	5732	4604	25
H8A	1261	7152	4154	32
H8B	3449	7676	4315	32
H1	3537	5561	7230	32
H20A	8101	3610	-729	46
H20B	6428	3009	-1802	46
H20C	7807	2683	-239	46
H3A	-958	3957	6696	37
H3B	-2227	4756	5897	37
H9A	4692	7014	6811	36
H9B	2980	7752	6651	36
H15A	5851	5496	2478	30
H15B	4257	5815	985	30
H7	5554	6481	4213	29
H12A	-223	7535	7411	42
H12B	-1022	6612	7772	42
H19A	2400	3009	406	48
H19B	4055	2285	271	48
H19C	2642	2724	-1180	48
H11A	1895	3300	5702	45
H11B	3497	4015	6497	45
H11C	3279	3817	4795	45
H2A	1596	4736	8320	43
H2B	-372	5390	8045	43

Experimental

Single crystals of $C_{20}H_{30}O_2$ compound **1** were colorless crystals. A suitable crystal was selected and on a XtaLAB AFC12 (RINC): Kappa single diffractometer. The crystal was kept at 100.00(10) K during data collection. Using Olex2 [1], the structure was solved with the SHELXT [2] structure solution program using Intrinsic

Phasing and refined with the SHELXL [3] refinement package using Least Squares minimisation.

1. Dolomanov, O.V., Bourhis, L.J., Gildea, R.J, Howard, J.A.K. & Puschmann, H. (2009), J. Appl. Cryst. 42, 339-341.
2. Sheldrick, G.M. (2015). Acta Cryst. A71, 3-8.
3. Sheldrick, G.M. (2015). Acta Cryst. C71, 3-8.

Refinement model description

Number of restraints - 1, number of constraints - unknown.

Details:
1. Fixed Uiso
At 1.2 times of:
All C(H) groups, All C(H,H) groups
At 1.5 times of:
All C(H,H,H) groups, All O(H) groups
2.a Ternary CH refined with riding coordinates:
C6(H6), C5(H5), C1(H1), C7(H7)
2.b Secondary CH2 refined with riding coordinates:
C8(H8A,H8B), C3(H3A,H3B), C9(H9A,H9B), C15(H15A,H15B), C2(H2A,H2B)
2.c Aromatic/amide H refined with riding coordinates:
C17(H17)
2.d X=CH2 refined with riding coordinates:
C12(H12A,H12B)
2.e Idealised Me refined as rotating group:
C14(H14A,H14B,H14C), C20(H20A,H20B,H20C), C19(H19A,H19B,H19C), C11(H11A,H11B,
H11C)
2.f Idealised tetrahedral OH refined as rotating group:
O20(H20)

This report has been created with Olex2, compiled on 2020.11.12 svn.r5f609507 for OlexSys. Please let us know if there are any errors or if you would like to have additional features.

ECD calculation for compounds 1–7

Molecular Merck force field (MMFF) and DFT/TDDFT calculations were performed with Spartan'14 software package (Wavefunction Inc., Irvine, CA, USA) and Gaussian09 [1] program package, Conflex conformational search generated low-energy conformers within a 10 kcal/mol energy was finished by software

CONFLEX 7. The predominant conformers were optimized by DFT calculations at B3LYP/6-311g(d,p) level with PCM in MeOH. The optimized conformers of **1**–**7** were used for the ECD calculation at the B3LYP/6-311g(d,p) level with PCM in MeOH. For comparisons of the calculated curves and experimental CD spectra, the program SpecDis 1.62 (Bruhn et al., 2013) was used. Percentages for each conformation are shown in Table S1.

Selected conformation of 1 and their percentage

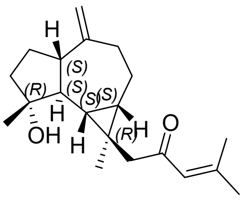

Figure S75. Model compound of **1**

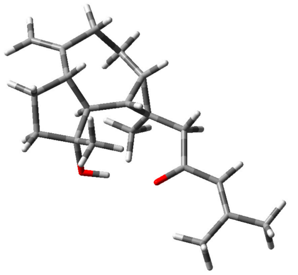

1-1 (49.65%)

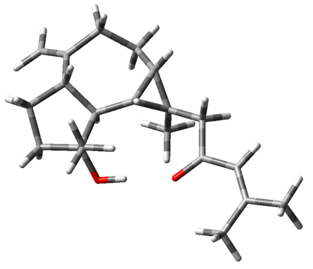

1-2 (49.86%)

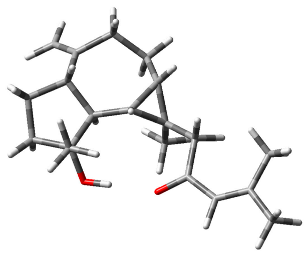

1-3 (0.16%)

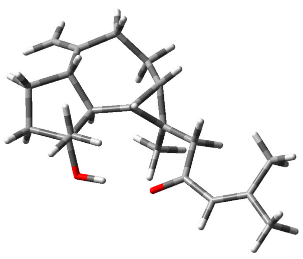

1-4 (0.16%)

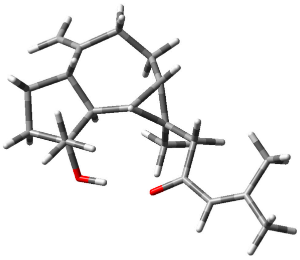

1-5 (0.16%)

Figure S76. The lowest energy conformers of **1** (the relative populations are in parentheses).

Selected conformation of 2 and their percentage

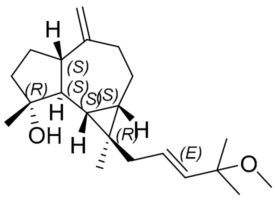

Figure S77. Model compound of **2**

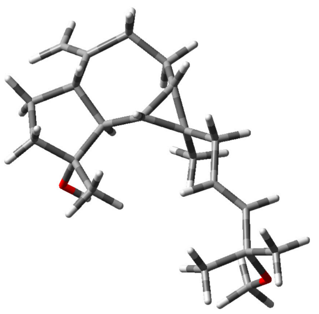

2-1 (8.32%)

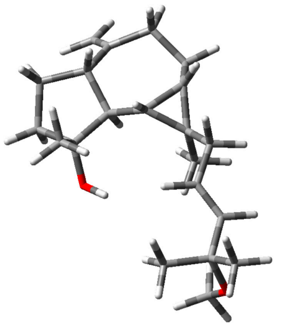

2-2 (2.42%)

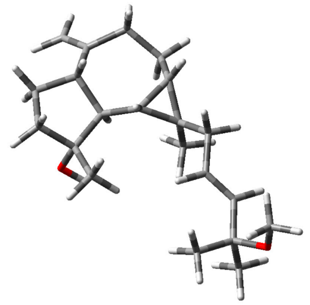

2-3 (7.15%)

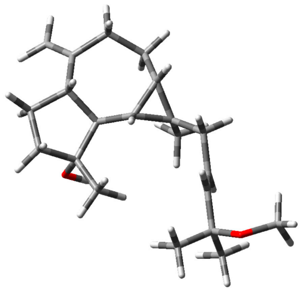

2-4 (3.11%)

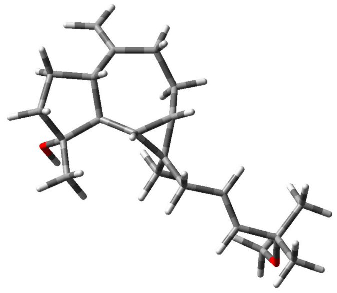

2-5 (33.36%)

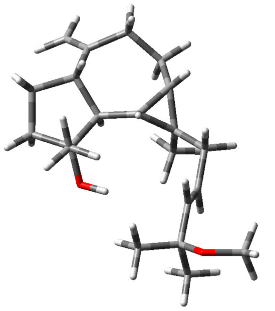

2-6 (1.64%)

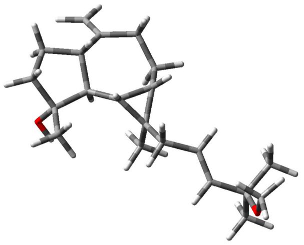

2-7 (27.86%)

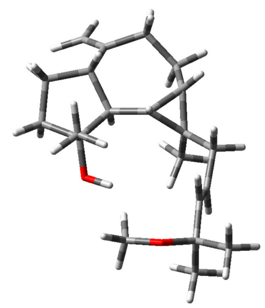

2-8 (0.51%)

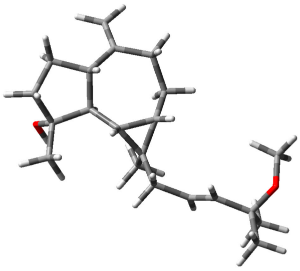

2-9 (12.94%)

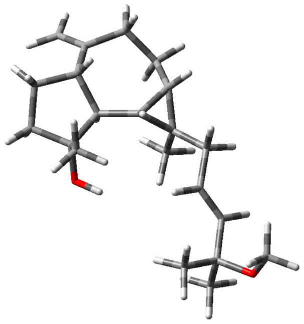

2-10 (2.71%)

Figure S78. The lowest energy conformers of **2** (the relative populations are in parentheses).

Selected conformation of 3 and 4 and their percentage

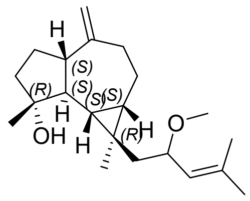

Figure S79. Model compounds of **3** and **4**

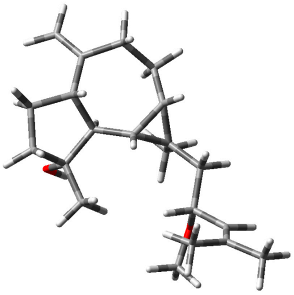

3/4-1 (7.36%)

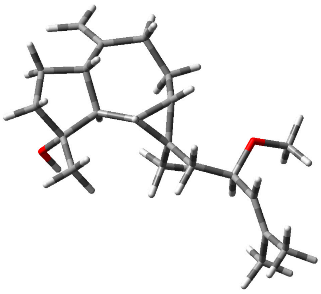

3/4-2 (84.13%)

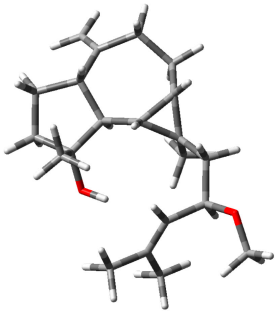

3/4-3 (0.31%)

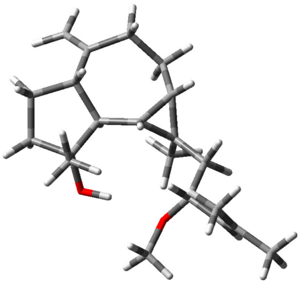

3/4-4 (4.91%)

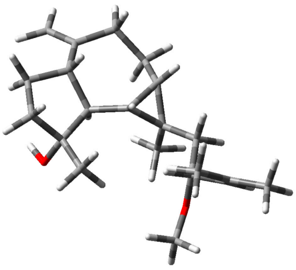

3/4-5 (3.29%)

Figure S80. The lowest energy conformers of **3/4** (the relative populations are in parentheses).

Selected conformation of 5 and their percentage

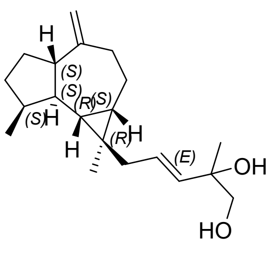

Figure S81. Model compound of **5**

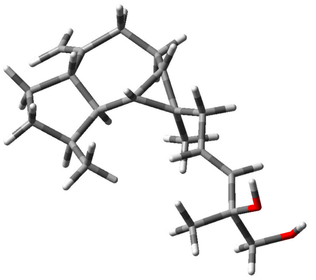

5-1 (1.24%)

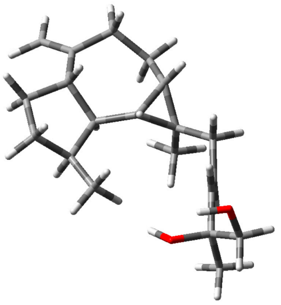

5-2 (0.64%)

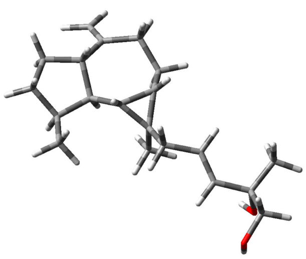

5-3 (6.08%)

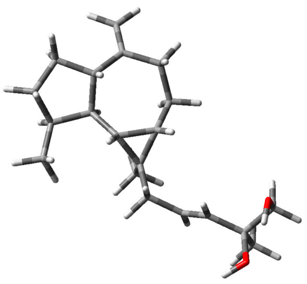 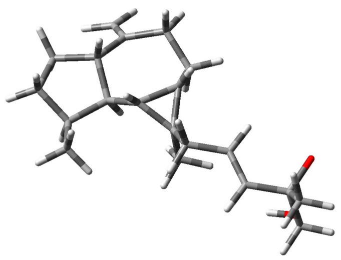 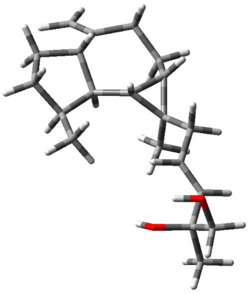

5-4 (2.79%) **5-5** (85.77%) **5-6** (3.49%)

Figure S82. The lowest energy conformers of **5** (the relative populations are in parentheses).

Selected conformation of 6 and their percentage

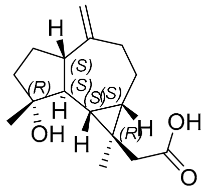

Figure S83. Model compound of **6**

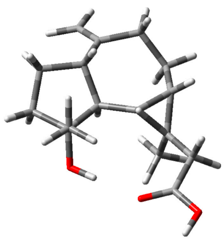 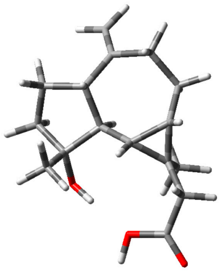 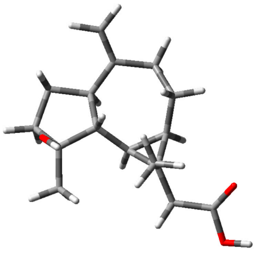 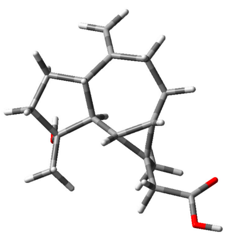

6-1 (74.50%) **6-2** (2.31%) **6-3** (7.53%) **6-4** (3.55%)

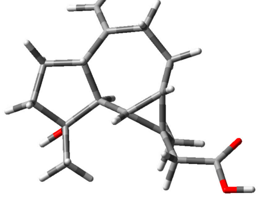

6-5 (12.10%)

Figure S84. The lowest energy conformers of **6** (the relative populations are in parentheses).

Selected conformation of 7 and their percentage

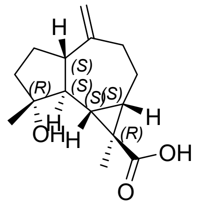

Figure S85. Model compound of **7**

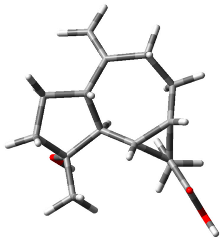

7-1 (16.14%)

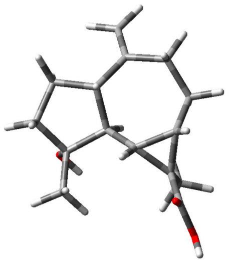

7-2 (16.12%)

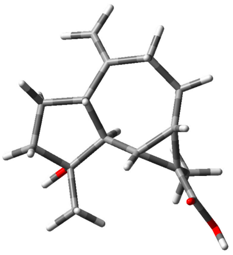

7-3 (35.09%)

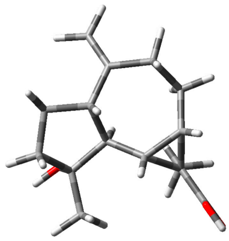

7-4 (26.22%)

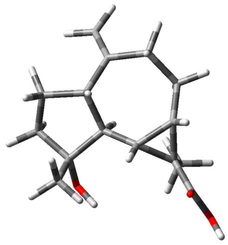

7-5 (3.21%)

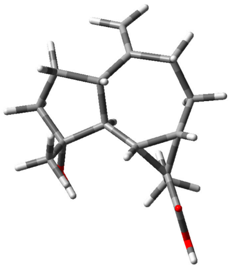

7-6 (3.21%)

Figure S86. The lowest energy conformers of **7** (the relative populations are in parentheses).

Table S3. Extracted heats and weighting factors of the optimized conformers of **1**–**7**, at B3LYP/6-311g(d,p) level

		B3LYP/6-311g(d,p)	
	Conformer	Extracted heats	Boltzmann-calculated contribution (%)
**1**	**1-1**	-930.44087	49.65%
	**1-2**	-930.44088	49.86%
	**1-3**	-930.43547	0.16%
	**1-4**	-930.43548	0.16%
	**1-5**	-930.43547	0.16%
**2**	**2-1**	-970.89336	8.32%
	**2-2**	-970.8922	2.42%
	**2-3**	-970.89322	7.15%
	**2-4**	-970.89243	3.11%
	**2-5**	-970.89467	33.36%
	**2-6**	-970.89183	1.64%
	**2-7**	-970.8945	27.86%
	**2-8**	-970.89072	0.51%
	**2-9**	-970.89378	12.94%
	**2-10**	-970.8923	2.71%
**3/4**	**3/4-1**	-970.89477	7.36%
	**3/4-2**	-970.89707	84.13%
	**3/4-3**	-970.89178	0.31%
	**3/4-4**	-970.89439	4.91%
	**3/4-5**	-970.89401	3.29%

**5**	**5-1**	-931.6045	1.24%
	**5-2**	-931.60387	0.64%
	**5-3**	-931.606	6.08%
	**5-4**	-931.60526	2.79%
	**5-5**	-931.6085	85.77%
	**5-6**	-931.60547	3.49%
**6**	**6-1**	-849.71333	74.50%
	**6-2**	-849.71005	2.31%
	**6-3**	-849.71117	7.53%
	**6-4**	-849.71046	3.55%
	**6-5**	-849.71162	12.10%
**7**	**7-1**	-810.41677	16.14%
	**7-2**	-810.41677	16.12%
	**7-3**	-810.4175	35.09%
	**7-4**	-810.41723	26.22%
	**7-5**	-810.41524	3.21%
	**7-6**	-810.41524	3.21%

Table S4. The Cartesian coordinates of the lowest energy conformers for **1–7**

**1-1**	X axis(Å)	Y axis(Å)	Z axis(Å)	**1-2**	X axis(Å)	Y axis(Å)	Z axis(Å)
C	-4.9271	-0.5501	0.8062	C	-4.9987	-0.3624	0.2389
C	-3.8663	-0.6957	-0.0074	C	-3.8288	-0.5256	-0.4039
C	-3.4578	-2.0945	-0.4536	C	-3.4334	-1.9154	-0.8888
C	-3.0094	0.4471	-0.5473	C	-2.83	0.5893	-0.7062
C	-3.4994	1.9051	-0.3588	C	-3.2595	2.0613	-0.4856
C	-2.1574	-2.5839	0.2007	C	-2.2845	-2.5367	-0.0804
C	-1.5919	0.4308	0.0779	C	-1.5407	0.4293	0.1383
C	-2.3784	2.6421	0.3727	C	-2.2435	2.662	0.4843
C	-1.1035	1.8935	-0.0226	C	-0.9628	1.8582	0.2475
O	-0.0823	2.1521	0.9351	O	-0.1127	1.9751	1.3834
C	-0.609	2.356	-1.3977	C	-0.21	2.3858	-0.9787
C	-0.9367	-2.0579	-0.4996	C	-0.9393	-2.0467	-0.537
C	-0.6864	-0.5708	-0.5875	C	-0.5948	-0.5783	-0.4585
C	0.2859	-1.4499	0.1952	C	0.1893	-1.5783	0.3878
C	1.655	-1.6454	-0.4477	C	1.6315	-1.8145	-0.0505
C	0.3512	-1.418	1.7071	C	0.0129	-1.669	1.8883
C	6.464	-0.3091	-0.3887	C	6.3484	-0.2773	-0.5815
C	5.042	0.0168	-0.014	C	4.9041	0.0852	-0.3544
C	4.0216	-0.6837	-0.5444	C	4.0056	-0.8679	-0.0413
C	4.9065	1.1448	0.9736	C	4.6025	1.5508	-0.5179
C	2.5687	-0.4353	-0.2935	C	2.557	-0.6418	0.2555
O	2.1463	0.6879	-0.0234	O	2.1554	0.4254	0.7159

H	-5.2653	0.4092	1.1784	H	-5.3367	0.5903	0.6278
H	-5.494	-1.41	1.1529	H	-5.664	-1.2022	0.4211
H	-3.3784	-2.1202	-1.5476	H	-3.1808	-1.8713	-1.9556
H	-4.2518	-2.8071	-0.1939	H	-4.2977	-2.5883	-0.8128
H	-2.9347	0.2873	-1.6331	H	-2.5847	0.4993	-1.7748
H	-3.6465	2.3588	-1.3474	H	-3.2026	2.5931	-1.4443
H	-4.4469	2.0229	0.1691	H	-4.2771	2.2092	-0.121
H	-2.1709	-2.3516	1.2689	H	-2.4565	-2.3792	0.9878
H	-2.1248	-3.6785	0.1319	H	-2.3023	-3.6235	-0.2306
H	-1.6873	0.1923	1.1445	H	-1.8262	0.1161	1.1501
H	-2.5348	2.585	1.4579	H	-2.5891	2.5354	1.5186
H	-2.348	3.7064	0.1162	H	-2.1096	3.7377	0.3282
H	0.7169	1.6547	0.648	H	0.6898	1.4338	1.2049
H	-0.4223	3.4364	-1.3904	H	0.0331	3.4469	-0.8478
H	0.3488	1.8934	-1.6561	H	0.7507	1.8774	-1.1086
H	-1.3227	2.1403	-2.1982	H	-0.7835	2.2804	-1.9042
H	-0.7623	-2.6262	-1.4107	H	-0.6549	-2.5561	-1.455
H	-0.3933	-0.2381	-1.5793	H	-0.1295	-0.1937	-1.3618
H	2.1384	-2.518	0.0072	H	2.0161	-2.7093	0.4526
H	1.553	-1.8426	-1.5218	H	1.6756	-1.9927	-1.1319
H	-0.6166	-1.2948	2.1958	H	-1.0084	-1.4964	2.2313
H	0.7817	-2.3542	2.0787	H	0.3037	-2.6668	2.2345
H	0.9874	-0.5959	2.05	H	0.6477	-0.9384	2.3991
H	6.5249	-1.1177	-1.1248	H	6.5375	-1.3455	-0.4311
H	6.9519	0.5706	-0.8213	H	6.647	-0.0278	-1.6049
H	7.0286	-0.6216	0.4958	H	6.9902	0.274	0.1136
H	4.2196	-1.5128	-1.2175	H	4.3118	-1.9085	0.0122
H	5.6644	1.0508	1.7594	H	3.5446	1.7584	-0.6931
H	5.0529	2.106	0.4711	H	4.9289	2.1046	0.3679
H	3.9416	1.1589	1.4853	H	5.1366	1.9511	-1.387
**1-3**	X axis(Å)	Y axis(Å)	Z axis(Å)	**1-4**	X axis(Å)	Y axis(Å)	Z axis(Å)
C	-4.76	-1.3445	0.624	C	-4.725	-1.2934	0.7728
C	-3.6419	-1.2449	-0.1166	C	-3.6778	-1.1566	-0.0598
C	-2.8953	-2.5139	-0.5096	C	-3.0216	-2.4034	-0.6406
C	-3.0357	0.062	-0.6224	C	-3.0696	0.1745	-0.4955
C	-3.8582	1.3692	-0.4921	C	-3.8243	1.487	-0.1655
C	-1.5671	-2.6913	0.2404	C	-1.6346	-2.6907	-0.0467
C	-1.6962	0.376	0.0893	C	-1.6593	0.3745	0.1139
C	-2.9805	2.3525	0.2818	C	-2.8464	2.3558	0.6243
C	-1.5474	1.9103	-0.0216	C	-1.4625	1.9067	0.1496
O	-0.67	2.4057	0.9843	O	-0.4854	2.268	1.1199
C	-1.0894	2.4571	-1.3786	C	-1.1013	2.5805	-1.1788
C	-0.4521	-1.9025	-0.3852	C	-0.5562	-1.877	-0.7039

C	-0.543	-0.3982	-0.4901	C	-0.5967	-0.3678	-0.6516
C	0.5455	-1.0251	0.3778	C	0.5462	-1.1114	0.035
C	1.9682	-0.9071	-0.1669	C	1.9099	-0.9821	-0.6369
C	0.492	-0.969	1.8895	C	0.6442	-1.2059	1.5421
C	6.48	0.1224	0.3038	C	6.4486	0.0711	-0.3895
C	5.0694	-0.136	-0.1634	C	5.0092	-0.1069	0.0145
C	4.0514	0.5722	0.361	C	4.0264	0.4758	-0.6962
C	4.9639	-1.1747	-1.2433	C	4.8075	-0.9295	1.2575
C	2.5873	0.4584	0.1013	C	2.581	0.3607	-0.3729
O	1.9088	1.4865	0.1752	O	1.9734	1.3645	-8.00E-04
H	-5.334	-0.4879	0.9551	H	-5.2303	-0.4556	1.2374
H	-5.1364	-2.3107	0.9494	H	-5.1078	-2.2746	1.0407
H	-2.7356	-2.5229	-1.595	H	-2.9678	-2.3129	-1.7327
H	-3.522	-3.3888	-0.2917	H	-3.6574	-3.2766	-0.443
H	-2.8555	-0.0806	-1.6982	H	-2.9942	0.1323	-1.5922
H	-4.0502	1.7674	-1.4968	H	-4.0806	1.9929	-1.1054
H	-4.8351	1.2701	-0.0168	H	-4.763	1.371	0.3782
H	-1.7086	-2.4657	1.3007	H	-1.6641	-2.5647	1.0389
H	-1.2813	-3.7497	0.1946	H	-1.395	-3.7474	-0.2198
H	-1.8054	0.135	1.1541	H	-1.679	0.0246	1.1535
H	-3.1853	2.2749	1.3575	H	-2.9609	2.1699	1.7003
H	-3.1778	3.3922	-2.00E-04	H	-3.0267	3.425	0.4704
H	0.2374	2.1093	0.7465	H	0.3872	1.9651	0.7809
H	-1.1543	3.5516	-1.3881	H	-1.1244	3.6713	-1.0699
H	-0.0376	2.2231	-1.5714	H	-0.0796	2.3369	-1.4873
H	-1.6857	2.0746	-2.212	H	-1.7809	2.3059	-1.9908
H	-0.0858	-2.4212	-1.2685	H	-0.3006	-2.3149	-1.6663
H	-0.2628	-0.0144	-1.467	H	-0.3997	0.1046	-1.6099
H	2.5796	-1.6899	0.295	H	2.5534	-1.7977	-0.2913
H	1.968	-1.0656	-1.2506	H	1.8118	-1.083	-1.725
H	-0.509	-1.0852	2.3078	H	-0.3155	-1.3212	2.0483
H	1.1119	-1.7671	2.3121	H	1.2593	-2.0689	1.8197
H	0.8789	-0.0123	2.2546	H	1.1159	-0.3091	1.9561
H	6.5247	0.8695	1.1034	H	6.5529	0.674	-1.2978
H	6.9242	-0.8013	0.6893	H	7.0088	0.5703	0.4078
H	7.0955	0.4861	-0.5256	H	6.91	-0.9029	-0.5827
H	4.2619	1.3608	1.0819	H	4.2383	1.1219	-1.542
H	4.0692	-1.0534	-1.8567	H	5.0801	-1.9726	1.0676
H	5.8124	-1.0968	-1.9323	H	5.4388	-0.5496	2.0679
H	4.9697	-2.1788	-0.8084	H	3.7776	-0.9082	1.6229
**1-5**	X axis(Å)	Y axis(Å)	Z axis(Å)				
C	-4.8195	-1.1771	-0.0013				
C	-3.5819	-1.1182	-0.5241				

C	-2.8377	-2.4144	-0.8239				
C	-2.8308	0.1687	-0.8584				
C	-3.5964	1.5148	-0.8281				
C	-1.6653	-2.6797	0.1332				
C	-1.6337	0.3797	0.1028				
C	-2.8531	2.4066	0.1646				
C	-1.4067	1.9074	0.1231				
O	-0.7347	2.3046	1.3136				
C	-0.6604	2.5013	-1.0759				
C	-0.4255	-1.9307	-0.2674				
C	-0.4294	-0.422	-0.3137				
C	0.4738	-1.1376	0.6883				
C	1.9723	-1.0643	0.3887				
C	0.1758	-1.1464	2.1724				
C	6.0384	0.5385	-1.2629				
C	4.8669	-0.0791	-0.5391				
C	3.8257	0.6952	-0.178				
C	5.0248	-1.5467	-0.2647				
C	2.5296	0.3503	0.4804				
O	1.9156	1.2687	1.0317				
H	-5.4	-0.2993	0.255				
H	-5.2963	-2.1298	0.2133				
H	-2.4954	-2.4051	-1.8663				
H	-3.5322	-3.2611	-0.7439				
H	-2.4621	0.0498	-1.888				
H	-3.5508	1.9692	-1.8264				
H	-4.6561	1.4523	-0.5759				
H	-1.9739	-2.4749	1.1621				
H	-1.424	-3.7495	0.0988				
H	-1.9452	0.0853	1.1124				
H	-3.2725	2.2813	1.1714				
H	-2.9471	3.469	-0.0841				
H	0.177	1.9372	1.2757				
H	-0.678	3.5966	-1.0301				
H	0.3982	2.2227	-1.0662				
H	-1.0891	2.1945	-2.0344				
H	0.0536	-2.4344	-1.1039				
H	0.0201	-0.0167	-1.216				
H	2.4993	-1.6984	1.1071				
H	2.162	-1.4459	-0.6207				
H	-0.8874	-1.1681	2.4169				
H	0.6288	-2.0306	2.634				
H	0.597	-0.2616	2.6592				

H	5.8872	1.6028	-1.4726				
H	6.9492	0.4447	-0.6622				
H	6.198	0.0328	-2.221				
H	3.8732	1.7648	-0.3829				
H	4.5812	-1.8372	0.6895				
H	4.5848	-2.1382	-1.073				
H	6.0849	-1.8148	-0.1915				
**2-1**	X axis(Å)	Y axis(Å)	Z axis(Å)	**2-2**	X axis(Å)	Y axis(Å)	Z axis(Å)
C	-3.2774	0.4676	-0.5868	C	-3.19	0.6366	-0.5482
C	-1.8595	0.4363	0.0583	C	-1.7639	0.4417	0.0284
C	-1.0251	-0.6384	-0.5998	C	-1.036	-0.6932	-0.6459
C	-1.4076	-2.0866	-0.3913	C	-1.5093	-2.1178	-0.4753
C	-2.5895	-2.4592	0.4579	C	-2.7328	-2.4283	0.3402
C	-3.9117	-2.0091	-0.1818	C	-4.0011	-1.7977	-0.2555
C	-4.2388	-0.5376	0.0361	C	-4.1739	-0.3277	0.1097
C	-3.6588	1.9518	-0.5403	C	-3.451	2.1595	-0.4455
C	-2.3518	2.6422	-0.9069	C	-2.233	2.7533	0.2577
C	-1.3074	1.8875	-0.0777	C	-1.0826	1.8189	-0.1206
C	-5.349	-0.1963	0.7142	C	-5.1505	0.0284	0.9628
C	-0.0707	-1.5751	0.1452	C	-0.1538	-1.6956	0.0996
C	1.1752	-2.0605	-0.5956	C	1.1012	-2.1709	-0.6295
C	0.155	-1.4139	1.6306	C	0.0383	-1.6177	1.5981
C	2.243	-1.0163	-0.7744	C	2.1455	-1.0974	-0.7918
C	0.0958	2.059	-0.6465	C	-0.5309	2.1556	-1.5081
O	-1.2903	2.4864	1.2299	O	-0.0217	1.9611	0.8198
C	3.4201	-1.0256	-0.1257	C	3.2977	-1.0553	-0.1013
C	4.553	-0.0234	-0.2477	C	4.3942	-0.0101	-0.195
C	5.7908	-0.7803	-0.7581	C	5.6654	-0.7124	-0.7007
C	4.2966	1.1606	-1.195	C	4.1019	1.1777	-1.127
O	4.9044	0.4228	1.0739	O	4.7133	0.4301	1.1367
C	3.9873	1.31	1.6993	C	3.7557	1.2705	1.7674
H	-0.7869	-0.3788	-1.6284	H	-0.7761	-0.4444	-1.6713
H	-1.3835	-2.7183	-1.2771	H	-1.5017	-2.7345	-1.3717
H	-1.9803	0.2213	1.1262	H	-1.8484	0.2395	1.1035
H	-3.1955	0.2	-1.6516	H	-3.1942	0.3972	-1.6218
H	-2.5225	-2.0765	1.4802	H	-2.632	-2.136	1.3892
H	-2.598	-3.5529	0.5432	H	-2.8546	-3.5187	0.3416
H	-4.7129	-2.6294	0.2409	H	-4.013	-1.9047	-1.3474
H	-3.9054	-2.2152	-1.2596	H	-4.8631	-2.3697	0.1121
H	-4.4598	2.1962	-1.2452	H	-3.5182	2.5744	-1.4597
H	-3.9711	2.2668	0.4621	H	-4.3753	2.449	0.0571
H	-2.3939	3.7102	-0.67	H	-2.386	2.7521	1.3448
H	-2.1635	2.5264	-1.9811	H	-2.0537	3.7936	-0.0336

H	-5.6401	0.8346	0.8834	H	-5.3187	1.0496	1.2824
H	-6.0139	-0.9485	1.1293	H	-5.8178	-0.7117	1.3966
H	0.9041	-2.4203	-1.5968	H	0.8436	-2.5351	-1.6323
H	1.5766	-2.9369	-0.0706	H	1.516	-3.0382	-0.1002
H	-0.7415	-1.1447	2.1916	H	-0.8457	-1.2899	2.1473
H	0.5324	-2.3494	2.0575	H	0.3133	-2.6031	1.9897
H	0.8964	-0.6308	1.8187	H	0.844	-0.9195	1.8454
H	2.0161	-0.2363	-1.4951	H	1.92	-0.3363	-1.5329
H	0.8341	1.5467	-0.0213	H	0.3596	1.5604	-1.7351
H	0.3822	3.1174	-0.648	H	-0.2042	3.2015	-1.545
H	0.1782	1.6901	-1.6729	H	-1.2653	2.0005	-2.3039
H	-0.6892	1.9585	1.784	H	0.5704	1.1979	0.6928
H	3.6102	-1.828	0.5875	H	3.4921	-1.8409	0.6292
H	6.6626	-0.1162	-0.7973	H	6.512	-0.0156	-0.7192
H	6.0638	-1.6002	-0.0821	H	5.9605	-1.5316	-0.0334
H	5.6307	-1.1978	-1.7584	H	5.5332	-1.1204	-1.709
H	5.1246	1.8776	-1.1501	H	4.8956	1.9304	-1.0545
H	3.3911	1.7141	-0.9235	H	3.1666	1.684	-0.863
H	4.1851	0.8306	-2.2339	H	4.0244	0.8618	-2.1733
H	4.0184	2.2952	1.2263	H	3.7386	2.2587	1.3001
H	2.9679	0.9155	1.6898	H	2.7575	0.8259	1.7566
H	4.2955	1.432	2.7416	H	4.0587	1.4009	2.8102
**2-3**	X axis(Å)	Y axis(Å)	Z axis(Å)	**2-4**	X axis(Å)	Y axis(Å)	Z axis(Å)
C	-3.3816	0.3744	-0.6423	C	-3.109	0.5365	-0.7798
C	-2.0047	0.4288	0.0855	C	-1.913	0.4094	0.2101
C	-1.0539	-0.5579	-0.5523	C	-0.9157	-0.5955	-0.3178
C	-1.337	-2.0371	-0.4111	C	-1.3142	-2.0523	-0.3905
C	-2.5338	-2.5262	0.3541	C	-2.6754	-2.5049	0.0548
C	-3.8477	-2.1544	-0.3494	C	-3.7814	-1.9615	-0.8619
C	-4.2976	-0.7197	-0.1074	C	-4.1826	-0.5234	-0.5626
C	-3.8783	1.8233	-0.5782	C	-3.5203	2.008	-0.6537
C	-2.6093	2.6211	-0.847	C	-2.178	2.7229	-0.5808
C	-1.5597	1.9214	0.0229	C	-1.3658	1.8581	0.3894
C	-5.467	-0.4851	0.5142	C	-5.44	-0.252	-0.1697
C	-0.0767	-1.4471	0.2205	C	-0.1816	-1.625	0.544
C	1.2428	-1.8134	-0.459	C	1.226	-2.037	0.11
C	0.0497	-1.3209	1.7213	C	-0.3739	-1.6469	2.0427
C	2.2579	-0.7041	-0.4851	C	2.3105	-1.0436	0.4324
C	-0.1432	2.2172	-0.4552	C	0.1322	2.0724	0.2118
O	-1.6666	2.4774	1.345	O	-1.6864	2.2972	1.7208
C	3.3976	-0.7119	0.2272	C	3.0912	-0.4616	-0.4951
C	4.4732	0.3574	0.263	C	4.2319	0.5144	-0.2707
C	4.2685	1.5402	-0.6981	C	3.8879	1.8218	-1.0038

C	4.5263	0.9134	1.6961	C	4.5336	0.8593	1.1969
O	5.7485	-0.2752	0.0576	O	5.3974	0.0072	-0.9454
C	6.0139	-0.734	-1.261	C	6.0252	-1.1202	-0.3503
H	-0.7761	-0.246	-1.5561	H	-0.4097	-0.2218	-1.2048
H	-1.2131	-2.6345	-1.3122	H	-1.0396	-2.5752	-1.3047
H	-2.1691	0.1707	1.1379	H	-2.3094	0.0772	1.1766
H	-3.2179	0.1484	-1.7074	H	-2.7416	0.401	-1.8088
H	-2.5553	-2.1753	1.3896	H	-2.8964	-2.2479	1.0945
H	-2.4632	-3.6199	0.4057	H	-2.6875	-3.6008	0.004
H	-4.6221	-2.8467	0.0061	H	-4.6554	-2.6167	-0.7525
H	-3.7642	-2.3225	-1.4307	H	-3.478	-2.0342	-1.9141
H	-4.6537	2.0291	-1.3228	H	-4.1159	2.3469	-1.5072
H	-4.2711	2.0819	0.412	H	-4.0882	2.2007	0.2641
H	-2.3507	2.5536	-1.9107	H	-2.3018	3.7534	-0.2325
H	-2.747	3.675	-0.5844	H	-1.7176	2.7405	-1.5761
H	-5.8444	0.5151	0.6969	H	-5.7855	0.7545	0.0389
H	-6.0951	-1.2982	0.8669	H	-6.1769	-1.0399	-0.0419
H	1.0625	-2.1126	-1.4997	H	1.2336	-2.25	-0.9671
H	1.6547	-2.7032	0.0343	H	1.4771	-2.9875	0.5979
H	-0.8964	-1.1428	2.2352	H	-1.3983	-1.4509	2.3639
H	0.4762	-2.2382	2.1411	H	-0.0931	-2.6266	2.4439
H	0.7135	-0.4886	1.9777	H	0.2579	-0.8899	2.5184
H	2.0211	0.1296	-1.1392	H	2.4596	-0.8304	1.4868
H	0.5957	1.7445	0.1998	H	0.7011	1.4741	0.9308
H	0.0586	3.2942	-0.4119	H	0.3963	3.1179	0.4106
H	0.0275	1.8875	-1.484	H	0.4691	1.8302	-0.8002
H	-1.0442	1.9937	1.9159	H	-1.2148	1.7135	2.3403
H	3.6016	-1.5706	0.8671	H	2.9231	-0.7126	-1.5425
H	5.1368	2.2091	-0.6781	H	4.7267	2.5265	-0.9531
H	4.1545	1.2086	-1.7361	H	3.7038	1.6451	-2.0707
H	3.3796	2.1252	-0.4367	H	3.0053	2.3099	-0.5782
H	5.3371	1.6446	1.7987	H	5.4244	1.4941	1.2695
H	4.7383	0.1203	2.4239	H	4.7403	-0.0354	1.7944
H	3.5862	1.3993	1.9807	H	3.6967	1.3873	1.6678
H	6.9361	-1.3216	-1.2326	H	6.5279	-0.8379	0.5786
H	5.213	-1.3759	-1.6377	H	5.3166	-1.9327	-0.1687
H	6.1777	0.1084	-1.9384	H	6.789	-1.4837	-1.0438
**2-5**	X axis(Å)	Y axis(Å)	Z axis(Å)	**2-6**	X axis(Å)	Y axis(Å)	Z axis(Å)
C	3.5823	0.5641	-0.4931	C	-3.0674	0.6921	-0.7129
C	2.471	-0.3473	0.1029	C	-1.8108	0.4305	0.1579
C	1.2063	-0.214	-0.7093	C	-0.936	-0.6525	-0.4207
C	0.4493	1.0935	-0.7003	C	-1.4297	-2.0789	-0.5032
C	0.9451	2.258	0.1083	C	-2.8105	-2.4491	-0.0414

C	2.2756	2.8002	-0.4363	C	-3.903	-1.7451	-0.8594
C	3.4994	2.0044	-6.00E-04	C	-4.1688	-0.3171	-0.3994
C	4.8687	-0.2198	-0.2076	C	-3.3649	2.2014	-0.5319
C	4.4597	-1.6546	-0.5149	C	-2.3376	2.72	0.4714
C	3.079	-1.7826	0.1388	C	-1.1247	1.8085	0.2789
C	4.4478	2.5991	0.7453	C	-5.32	-0.0335	0.2352
C	-0.2027	-0.159	-0.116	C	-0.2566	-1.7341	0.4191
C	-1.3093	-0.8162	-0.9392	C	1.1325	-2.1749	-0.0378
C	-0.4139	-0.2982	1.3739	C	-0.4368	-1.7941	1.92
C	-2.6386	-0.1152	-0.8547	C	2.2502	-1.2712	0.4139
C	2.2611	-2.9079	-0.4883	C	-0.2866	2.2557	-0.9218
O	3.2898	-2.1463	1.5134	O	-0.3	1.8593	1.439
C	-3.7599	-0.6903	-0.3868	C	2.9246	-0.4432	-0.4025
C	-5.1367	-0.066	-0.2569	C	4.0781	0.4761	-0.044
C	-5.2298	1.4235	-0.6266	C	3.616	1.9236	-0.2804
C	-6.0906	-0.8459	-1.1774	C	4.5875	0.3712	1.403
O	-5.62	-0.3097	1.0762	O	5.1369	0.2607	-0.9938
C	-5.0012	0.4401	2.1128	C	5.8553	-0.9586	-0.8647
H	1.3319	-0.6464	-1.6993	H	-0.4312	-0.3162	-1.3224
H	0.0831	1.442	-1.6638	H	-1.2	-2.6093	-1.4253
H	2.2978	-0.0285	1.1368	H	-2.1376	0.1413	1.1647
H	3.4723	0.6016	-1.5878	H	-2.8173	0.5553	-1.7754
H	1.0439	2.0275	1.1732	H	-2.9704	-2.2633	1.024
H	0.1897	3.0503	0.0373	H	-2.9189	-3.5326	-0.1771
H	2.3742	3.8393	-0.0956	H	-4.8235	-2.3367	-0.7683
H	2.2519	2.8422	-1.5326	H	-3.6511	-1.7436	-1.9272
H	5.7028	0.1087	-0.8355	H	-3.2163	2.7079	-1.4946
H	5.1736	-0.1429	0.8427	H	-4.3795	2.4507	-0.2174
H	5.1897	-2.3645	-0.1127	H	-2.1066	3.7792	0.3165
H	4.3974	-1.7915	-1.6012	H	-2.7276	2.6285	1.4934
H	5.3475	2.0869	1.0681	H	-5.5672	0.9532	0.6077
H	4.3541	3.6338	1.0626	H	-6.0629	-0.8034	0.4264
H	-1.4001	-1.8644	-0.6272	H	1.1544	-2.2664	-1.1319
H	-1.0226	-0.8447	-1.9985	H	1.3281	-3.1849	0.3439
H	0.3922	0.1181	1.9803	H	-1.4441	-1.549	2.2604
H	-1.334	0.211	1.6778	H	-0.21	-2.8023	2.2833
H	-0.5058	-1.3564	1.6417	H	0.2422	-1.0935	2.4166
H	-2.6484	0.9128	-1.2064	H	2.5125	-1.3397	1.4662
H	1.2781	-2.992	-0.0119	H	0.6256	1.6579	-1.0117
H	2.7584	-3.8732	-0.3365	H	0.0491	3.2908	-0.7885
H	2.1208	-2.7701	-1.5645	H	-0.8343	2.1941	-1.8667
H	2.418	-2.1742	1.9449	H	0.3632	1.1518	1.3436
H	-3.707	-1.7237	-0.0441	H	2.6395	-0.4091	-1.4542

H	-6.23	1.8147	-0.4066	H	4.4411	2.6263	-0.113
H	-4.5235	2.0325	-0.0514	H	3.2937	2.0751	-1.318
H	-5.023	1.5904	-1.6896	H	2.7885	2.2013	0.3819
H	-7.1191	-0.4811	-1.0686	H	5.4807	0.9908	1.5443
H	-6.1165	-1.9107	-0.9146	H	4.875	-0.6538	1.6621
H	-5.8039	-0.7584	-2.2313	H	3.8283	0.6954	2.1237
H	-3.9123	0.3478	2.0876	H	6.4872	-0.9429	0.0273
H	-5.296	1.4916	2.0592	H	5.1877	-1.8243	-0.8438
H	-5.3541	0.0447	3.0696	H	6.5112	-1.0555	-1.7347
**2-7**	X axis(Å)	Y axis(Å)	Z axis(Å)	**2-8**	X axis(Å)	Y axis(Å)	Z axis(Å)
C	3.6147	0.5041	-0.5965	C	-2.993	0.7475	-0.6807
C	2.5238	-0.3626	0.0962	C	-1.7184	0.434	0.147
C	1.2038	-0.1921	-0.6149	C	-0.9144	-0.6868	-0.4636
C	0.495	1.1412	-0.5602	C	-1.4868	-2.085	-0.5134
C	1.0935	2.2945	0.1932	C	-2.8553	-2.3856	0.0293
C	2.3941	2.785	-0.4615	C	-3.9587	-1.6449	-0.741
C	3.6208	1.9506	-0.1155	C	-4.1302	-0.1967	-0.2993
C	4.8921	-0.3221	-0.4053	C	-3.2049	2.2728	-0.5207
C	4.4107	-1.7442	-0.6627	C	-2.1351	2.7484	0.4579
C	3.0831	-1.818	0.1002	C	-0.9703	1.7801	0.246
C	4.6455	2.518	0.5457	C	-5.2346	0.1591	0.3805
C	-0.1505	-0.0831	0.0879	C	-0.2461	-1.8033	0.3392
C	-1.3412	-0.7078	-0.6372	C	1.0752	-2.3461	-0.2013
C	-0.2461	-0.2039	1.5915	C	-0.3432	-1.8547	1.8474
C	-2.6352	0.0373	-0.4486	C	2.3081	-1.6017	0.2414
C	2.1796	-2.9193	-0.4466	C	-0.1343	2.1793	-0.9726
O	3.3907	-2.1768	1.4577	O	-0.1232	1.799	1.3912
C	-3.7269	-0.4933	0.1288	C	3.0384	-0.8107	-0.564
C	-5.068	0.1755	0.3648	C	4.3372	-0.0937	-0.2375
C	-5.2909	0.2456	1.885	C	4.847	-0.2621	1.204
C	-5.2125	1.5969	-0.2032	C	5.4167	-0.6423	-1.187
O	-6.1016	-0.6965	-0.1262	O	4.1868	1.2882	-0.6033
C	-6.2137	-0.7998	-1.5391	C	3.4762	2.1041	0.3172
H	1.2353	-0.637	-1.6068	H	-0.4392	-0.3759	-1.3902
H	0.0649	1.4942	-1.4952	H	-1.3395	-2.6258	-1.4464
H	2.4442	-0.0289	1.1368	H	-2.0212	0.1571	1.1649
H	3.419	0.536	-1.6794	H	-2.7951	0.5773	-1.7493
H	1.269	2.07	1.2494	H	-2.9476	-2.1759	1.0984
H	0.3624	3.1122	0.1734	H	-3.0191	-3.4648	-0.0812
H	2.5551	3.8228	-0.1416	H	-4.8993	-2.1895	-0.5855
H	2.2844	2.8184	-1.5529	H	-3.7679	-1.6758	-1.821
H	5.6841	-0.0286	-1.1013	H	-3.0425	2.7551	-1.4935
H	5.2823	-0.2469	0.6165	H	-4.2005	2.5816	-0.1979

H	5.1457	-2.4759	-0.3119	H	-2.5077	2.6808	1.4882
H	4.2571	-1.8882	-1.7391	H	-1.8578	3.7946	0.2911
H	5.55	1.9774	0.8019	H	-5.4116	1.1643	0.7436
H	4.6129	3.5581	0.8578	H	-6.0074	-0.5665	0.6204
H	-1.44	-1.7508	-0.3105	H	1.0437	-2.3764	-1.2988
H	-1.1438	-0.7513	-1.7163	H	1.1773	-3.3924	0.1143
H	0.6155	0.1975	2.1276	H	-1.3075	-1.5335	2.2447
H	-1.1272	0.3301	1.9626	H	-0.1738	-2.8786	2.1979
H	-0.3417	-1.2572	1.8764	H	0.415	-1.21	2.3032
H	-2.6428	1.0612	-0.8123	H	2.6246	-1.7858	1.2645
H	1.2356	-2.9648	0.1077	H	0.7448	1.5352	-1.078
H	2.6541	-3.9001	-0.3238	H	0.2555	3.1968	-0.8512
H	1.9583	-2.7861	-1.5097	H	-0.7036	2.1414	-1.9059
H	2.5563	-2.1671	1.9585	H	0.4445	1.0096	1.3295
H	-3.6769	-1.524	0.4802	H	2.6932	-0.6587	-1.587
H	-6.2809	0.6585	2.1133	H	5.7404	0.3505	1.3724
H	-5.2664	-0.7532	2.3381	H	4.1038	0.0607	1.9417
H	-4.5361	0.8667	2.3804	H	5.1017	-1.305	1.4234
H	-6.2385	1.9615	-0.0761	H	6.3642	-0.1086	-1.0458
H	-5.0005	1.6314	-1.2776	H	5.138	-0.493	-2.2376
H	-4.5349	2.3015	0.2918	H	5.5966	-1.7114	-1.0273
H	-6.9234	-1.6017	-1.7617	H	3.2236	3.0421	-0.1848
H	-6.6134	0.1247	-1.9644	H	2.5516	1.628	0.6486
H	-5.2578	-1.0522	-2.0062	H	4.1069	2.3433	1.178
**2-9**	X axis(Å)	Y axis(Å)	Z axis(Å)	**2-10**	X axis(Å)	Y axis(Å)	Z axis(Å)
C	3.1264	0.9063	-0.6823	C	-3.3376	0.5092	-0.6155
C	2.4589	-0.2745	0.0801	C	-1.9447	0.4346	0.0645
C	1.0323	-0.4441	-0.3813	C	-1.0618	-0.5959	-0.5937
C	0.0162	0.628	-0.0662	C	-1.4129	-2.0635	-0.503
C	0.4112	1.858	0.6986	C	-2.6466	-2.5189	0.2244
C	1.3852	2.7347	-0.1032	C	-3.933	-1.9965	-0.434
C	2.8333	2.2639	-0.0536	C	-4.2684	-0.5601	-0.0493
C	4.5915	0.4678	-0.793	C	-3.753	1.9961	-0.5075
C	4.4716	-1.0143	-1.1235	C	-2.6674	2.6794	0.3185
C	3.379	-1.5085	-0.1684	C	-1.3994	1.8779	0.0177
C	3.7723	3.0586	0.4902	C	-5.3279	-0.3195	0.743
C	-0.1453	-0.7647	0.5425	C	-0.1394	-1.5457	0.1709
C	-1.2496	-1.6463	-0.0402	C	1.1907	-1.8923	-0.4951
C	0.0811	-0.9951	2.0185	C	-0.0435	-1.5036	1.68
C	-2.6419	-1.2183	0.3417	C	2.1808	-0.7576	-0.4933
C	2.7186	-2.7852	-0.6808	C	-0.7653	2.3189	-1.3035
O	4.019	-1.8484	1.073	O	-0.4481	2.0935	1.0559
C	-3.5302	-0.7072	-0.5284	C	3.3096	-0.7471	0.2359

C	-4.939	-0.225	-0.2398	C	4.3574	0.3483	0.304
C	-5.9078	-1.0974	-1.0554	C	4.1297	1.5456	-0.6337
C	-5.3734	-0.2807	1.234	C	4.3835	0.8752	1.7488
O	-5.0817	1.1013	-0.7798	O	5.6498	-0.2476	0.0971
C	-4.3892	2.1342	-0.0912	C	5.9397	-0.6688	-1.2288
H	0.995	-0.7999	-1.4083	H	-0.7593	-0.282	-1.5892
H	-0.6621	0.898	-0.8734	H	-1.2954	-2.6399	-1.4183
H	2.4899	-0.0404	1.1501	H	-2.086	0.179	1.1221
H	2.7304	0.9454	-1.7088	H	-3.2412	0.2969	-1.6905
H	0.835	1.6327	1.6817	H	-2.6396	-2.2523	1.285
H	-0.5037	2.4337	0.8864	H	-2.6589	-3.6155	0.1893
H	1.3181	3.7552	0.2959	H	-4.7557	-2.658	-0.1321
H	1.0686	2.7984	-1.1521	H	-3.8697	-2.0743	-1.5266
H	5.1309	1.0158	-1.5718	H	-3.7656	2.4325	-1.5148
H	5.1306	0.5894	0.1539	H	-4.7441	2.1816	-0.0901
H	5.4284	-1.5264	-0.9789	H	-2.5699	3.7425	0.0746
H	4.1648	-1.131	-2.17	H	-2.9129	2.6221	1.387
H	4.8202	2.783	0.5353	H	-5.6101	0.673	1.0732
H	3.52	4.0264	0.9144	H	-5.9504	-1.1294	1.114
H	-1.0985	-2.6801	0.2951	H	1.0273	-2.1841	-1.5406
H	-1.1611	-1.6787	-1.1341	H	1.6131	-2.7783	-0.0044
H	0.8832	-0.3919	2.4471	H	-0.9853	-1.2775	2.1824
H	-0.8267	-0.7642	2.5852	H	0.2992	-2.4726	2.0586
H	0.3361	-2.0452	2.1977	H	0.6773	-0.7429	1.997
H	-2.9056	-1.346	1.3875	H	1.9334	0.08	-1.1387
H	1.9417	-3.1306	0.0102	H	0.197	1.8222	-1.4652
H	3.4531	-3.5966	-0.7461	H	-0.5439	3.3925	-1.2809
H	2.2757	-2.6549	-1.6727	H	-1.4081	2.1223	-2.1665
H	3.3211	-2.1063	1.7001	H	0.2387	1.4091	0.9632
H	-3.2264	-0.5927	-1.569	H	3.5259	-1.6098	0.8665
H	-6.9395	-0.7442	-0.9391	H	4.9784	2.2381	-0.5895
H	-5.6869	-1.042	-2.1286	H	4.0364	1.2339	-1.6799
H	-5.8672	-2.1487	-0.7495	H	3.2215	2.0993	-0.37
H	-6.3607	0.1779	1.3635	H	5.1741	1.6247	1.8742
H	-4.6862	0.2715	1.8847	H	4.6098	0.0727	2.4619
H	-5.4233	-1.3115	1.6023	H	3.4286	1.3304	2.0349
H	-4.4647	3.0459	-0.6909	H	6.8754	-1.2346	-1.2042
H	-4.8555	2.3346	0.8772	H	5.1584	-1.3206	-1.6289
H	-3.3291	1.8995	0.0374	H	6.09	0.193	-1.8846
**3/4-1**	X axis(Å)	Y axis(Å)	Z axis(Å)	**3/4-2**	X axis(Å)	Y axis(Å)	Z axis(Å)
C	-5.1686	-0.8302	-0.2534	C	4.5662	2.1558	0.7637
C	-3.8814	-0.8954	-0.6374	C	3.4703	1.7506	0.0973
C	-3.2485	-2.2501	-0.9243	C	2.2988	2.7087	-0.0789

C	-2.9924	0.3225	-0.8555	C	3.3265	0.3787	-0.5522
C	-3.6396	1.7091	-0.762	C	4.5427	-0.555	-0.5333
C	-2.0665	-2.5921	-0.0058	C	0.9953	2.2379	0.584
C	-1.8012	0.4024	0.1487	C	2.2053	-0.4857	0.0934
C	-2.4324	2.6323	-0.6709	C	3.9397	-1.8959	-0.9314
C	-1.5234	1.9242	0.3386	C	2.6472	-1.9671	-0.1102
O	-1.973	2.3042	1.651	O	2.9873	-2.5146	1.175
C	-0.081	2.3988	0.2261	C	1.635	-2.9228	-0.7353
C	-0.8023	-1.9156	-0.454	C	0.2758	1.231	-0.2654
C	-0.6396	-0.4136	-0.3768	C	0.8698	-0.1344	-0.5141
C	0.2516	-1.3189	0.479	C	-0.4394	-0.007	0.2701
C	1.7029	-1.5537	0.0286	C	-1.707	-0.4636	-0.4667
C	0.0689	-1.3521	1.9779	C	-0.4678	-0.3078	1.7509
C	5.7667	-0.0574	-1.9686	C	-6.3607	-1.486	-0.5601
C	4.4448	0.2386	-1.3086	C	-5.1656	-0.8505	0.1019
C	3.9621	-0.5766	-0.3493	C	-4.1885	-0.2923	-0.6406
C	3.763	1.4761	-1.8213	C	-5.1839	-0.915	1.6038
C	2.6828	-0.4397	0.4413	C	-2.9406	0.4109	-0.1648
O	3.0123	-0.6078	1.8275	O	-2.8234	1.6308	-0.9129
C	3.5063	0.5851	2.4249	C	-3.5504	2.7038	-0.3284
H	-0.4508	-2.3849	-1.3713	H	-0.1641	1.7249	-1.13
H	-0.202	0.0427	-1.2611	H	0.8197	-0.4532	-1.5528
H	-2.1391	-0.001	1.1107	H	2.2043	-0.2786	1.1692
H	-2.5944	0.2354	-1.8785	H	3.0826	0.5579	-1.6106
H	-5.6749	0.1074	-0.0526	H	5.4358	1.5231	0.9028
H	-5.7678	-1.7272	-0.1241	H	4.6304	3.1494	1.1982
H	-2.9382	-2.2817	-1.9765	H	2.142	2.8836	-1.1509
H	-4.0014	-3.0403	-0.806	H	2.5562	3.6849	0.3526
H	-4.2608	1.9362	-1.6341	H	5.3217	-0.2357	-1.2327
H	-4.254	1.8189	0.139	H	4.9852	-0.635	0.4665
H	-2.3311	-2.3726	1.032	H	1.2006	1.8744	1.5953
H	-1.8911	-3.6741	-0.0515	H	0.3299	3.1024	0.7003
H	-2.7307	3.6346	-0.3466	H	4.6305	-2.7165	-0.712
H	-1.9505	2.7079	-1.6532	H	3.7255	-1.8946	-2.007
H	-1.4573	1.7904	2.2972	H	2.1794	-2.5053	1.7172
H	-0.0171	3.4782	0.408	H	2.0353	-3.9433	-0.7583
H	0.5426	1.9266	0.9914	H	0.7167	-2.9679	-0.1393
H	0.3475	2.2014	-0.7603	H	1.3764	-2.6423	-1.7607
H	2.0311	-2.5182	0.4385	H	-1.5206	-0.4638	-1.5492
H	1.7178	-1.6527	-1.0653	H	-1.8994	-1.5056	-0.1795
H	-0.9728	-1.4136	2.2973	H	0.4574	-0.0606	2.274
H	0.5864	-2.2168	2.4066	H	-1.2657	0.255	2.2444
H	0.481	-0.444	2.4288	H	-0.6538	-1.3749	1.913

H	6.2392	-0.9606	-1.5683	H	-6.3187	-1.4149	-1.652
H	5.6291	-0.2036	-3.0449	H	-6.4197	-2.5475	-0.2982
H	6.4607	0.7757	-1.8158	H	-7.2816	-0.9939	-0.2306
H	4.5487	-1.4527	-0.0711	H	-4.282	-0.3201	-1.7265
H	4.3617	2.3613	-1.583	H	-6.0119	-0.3154	1.9957
H	3.6498	1.4193	-2.9093	H	-5.3221	-1.9504	1.9336
H	2.7652	1.627	-1.4056	H	-4.262	-0.5559	2.066
H	2.2076	0.5321	0.2971	H	-2.9834	0.6472	0.901
H	3.7257	0.3742	3.4751	H	-3.4263	3.5857	-0.9626
H	4.4284	0.9172	1.9395	H	-4.617	2.4689	-0.2659
H	2.751	1.3757	2.3821	H	-3.1554	2.935	0.6656
**3/4-3**	X axis(Å)	Y axis(Å)	Z axis(Å)	**3/4-4**	X axis(Å)	Y axis(Å)	Z axis(Å)
C	-4.8984	-0.6859	0.8389	C	-5.0807	-0.7243	-0.0954
C	-3.8418	-0.8613	0.0258	C	-3.8411	-0.8157	-0.6086
C	-3.3873	-2.2735	-0.3164	C	-3.2647	-2.1883	-0.9338
C	-3.0229	0.2618	-0.604	C	-2.9247	0.3691	-0.9011
C	-3.5891	1.7014	-0.5587	C	-3.5208	1.7965	-0.8718
C	-2.0456	-2.6418	0.3313	C	-2.1243	-2.6045	0.0076
C	-1.6179	0.359	0.0549	C	-1.7424	0.4068	0.1056
C	-2.5762	2.5252	0.2276	C	-2.7712	2.5428	0.2263
C	-1.2345	1.8497	-0.0622	C	-1.3817	1.9021	0.2398
O	-0.3028	2.2104	0.9519	O	-0.7573	2.1668	1.4923
C	-0.6741	2.2983	-1.4142	C	-0.4937	2.4839	-0.8618
C	-0.8771	-2.0902	-0.4347	C	-0.8129	-1.9827	-0.3826
C	-0.637	-0.6044	-0.5739	C	-0.6085	-0.4854	-0.3409
C	0.3661	-1.4645	0.2027	C	0.2016	-1.383	0.5966
C	1.6764	-1.8531	-0.4889	C	1.6812	-1.6112	0.2511
C	0.4702	-1.3621	1.7064	C	-0.0755	-1.4056	2.083
C	2.9926	2.8582	-0.4549	C	5.789	-0.289	-1.7628
C	3.067	1.4984	0.1927	C	4.4009	0.0283	-1.2703
C	2.866	0.3807	-0.5352	C	3.9089	-0.5794	-0.172
C	3.3993	1.5381	1.659	C	3.6647	1.0387	-2.1047
C	2.9343	-1.058	-0.087	C	2.5675	-0.3723	0.4886
O	4.0347	-1.677	-0.7762	O	2.7927	-0.2281	1.8976
C	5.2889	-1.4334	-0.1511	C	3.193	1.088	2.2623
H	-0.732	-2.6776	-1.3402	H	-0.4216	-2.4912	-1.2617
H	-0.3632	-0.2945	-1.5786	H	-0.1105	-0.0869	-1.2207
H	-1.7148	0.1342	1.1248	H	-2.1057	0.0753	1.0868
H	-2.918	0.0094	-1.6694	H	-2.5426	0.2198	-1.9218
H	-5.2588	0.2896	1.1428	H	-5.5418	0.2165	0.1805
H	-5.4364	-1.5334	1.2554	H	-5.6807	-1.611	0.091
H	-3.3392	-2.3875	-1.4065	H	-2.9321	-2.2045	-1.9792
H	-4.1379	-2.996	0.03	H	-4.0557	-2.9459	-0.8573

H	-3.6505	2.0889	-1.584	H	-3.3212	2.2818	-1.8362
H	-4.593	1.8031	-0.1428	H	-4.601	1.8554	-0.7272
H	-2.051	-2.3422	1.3826	H	-2.4076	-2.3962	1.0427
H	-1.9481	-3.7347	0.3308	H	-2.0002	-3.6927	-0.0573
H	-2.5928	3.5827	-0.0566	H	-3.2707	2.396	1.1927
H	-2.804	2.4829	1.3006	H	-2.7429	3.6228	0.0474
H	0.4788	1.6422	0.8283	H	-0.0439	1.5137	1.6031
H	-0.5432	3.3866	-1.4311	H	-0.4221	3.5732	-0.7595
H	0.3199	1.877	-1.5909	H	0.5319	2.1119	-0.7783
H	-1.319	2.019	-2.2525	H	-0.8649	2.2603	-1.8662
H	1.862	-2.915	-0.2733	H	2.0473	-2.4612	0.8416
H	1.5573	-1.8106	-1.5812	H	1.7552	-1.9057	-0.8043
H	-0.4886	-1.2488	2.2148	H	-1.1358	-1.3733	2.3391
H	0.9474	-2.2602	2.113	H	0.3342	-2.3181	2.5291
H	1.0737	-0.4947	1.989	H	0.3887	-0.5472	2.5758
H	2.7563	2.7986	-1.5224	H	6.2992	-1.0207	-1.1276
H	2.2181	3.4657	0.0241	H	5.7472	-0.7007	-2.7765
H	3.9517	3.3771	-0.3567	H	6.4007	0.6191	-1.7803
H	2.6589	0.4899	-1.6	H	4.5355	-1.3095	0.3411
H	4.3501	2.059	1.8143	H	4.1479	2.0177	-2.0238
H	2.6185	2.0762	2.2065	H	3.6739	0.7357	-3.1573
H	3.4962	0.55	2.1122	H	2.6173	1.1553	-1.8198
H	3.0878	-1.1645	0.9898	H	2.0545	0.519	0.1186
H	6.0605	-1.9593	-0.7201	H	3.3263	1.1147	3.3472
H	5.5294	-0.3667	-0.1513	H	4.1429	1.3559	1.791
H	5.2898	-1.8213	0.8721	H	2.4194	1.8128	1.9912
**3/4-5**	X axis(Å)	Y axis(Å)	Z axis(Å)				
C	-5.1762	-0.8067	-0.2408				
C	-3.894	-0.8572	-0.6441				
C	-3.2705	-2.2009	-0.9994				
C	-3.0031	0.365	-0.826				
C	-3.6504	1.7461	-0.6699				
C	-2.0747	-2.5864	-0.1173				
C	-1.7971	0.4005	0.1651				
C	-2.4472	2.6688	-0.5406				
C	-1.5157	1.9139	0.4148				
O	-1.8955	2.2417	1.7634				
C	-0.0752	2.3876	0.2789				
C	-0.8167	-1.8933	-0.5562				
C	-0.6475	-0.3964	-0.4145				
C	0.2527	-1.3422	0.3864				
C	1.6965	-1.5574	-0.0967				
C	0.092	-1.4415	1.8852				

C	5.7345	0.1345	-2.0068				
C	4.438	0.3706	-1.276				
C	3.955	-0.5567	-0.4248				
C	3.7833	1.686	-1.5923				
C	2.6979	-0.5028	0.4106				
O	3.0685	-0.8333	1.7567				
C	3.5339	0.2947	2.4883				
H	-0.4789	-2.3231	-1.4975				
H	-0.2193	0.0978	-1.2827				
H	-2.1182	-0.0434	1.1146				
H	-2.6191	0.319	-1.8569				
H	-5.6838	0.1199	0.0015				
H	-5.7737	-1.7093	-0.1434				
H	-2.9782	-2.1879	-2.0571				
H	-4.0242	-2.9933	-0.9027				
H	-4.2734	2.0098	-1.5305				
H	-4.2667	1.8165	0.2333				
H	-2.3216	-2.4127	0.9334				
H	-1.9033	-3.6657	-0.2141				
H	-2.7456	3.65	-0.1575				
H	-1.9806	2.8015	-1.5242				
H	-2.7931	1.9042	1.924				
H	-0.0014	3.4599	0.497				
H	0.5663	1.8869	1.0104				
H	0.3259	2.2253	-0.7252				
H	2.0105	-2.561	0.2197				
H	1.7001	-1.5545	-1.1952				
H	-0.9451	-1.5102	2.2178				
H	0.6091	-2.3286	2.2657				
H	0.5179	-0.5577	2.3701				
H	6.1875	-0.8291	-1.751				
H	5.5672	0.146	-3.0889				
H	6.4576	0.9179	-1.7574				
H	4.5243	-1.476	-0.2842				
H	4.428	2.5124	-1.2757				
H	3.6169	1.7727	-2.6714				
H	2.8146	1.8183	-1.1078				
H	2.2268	0.4814	0.3912				
H	3.7968	-0.0376	3.4961				
H	4.4248	0.7257	2.0225				
H	2.7465	1.0505	2.568				
**5-1**	X axis(Å)	Y axis(Å)	Z axis(Å)	**5-2**	X axis(Å)	Y axis(Å)	Z axis(Å)
C	-3.5384	0.6317	-0.2929	C	-3.0864	0.9049	-0.5942

C	-2.0981	0.5601	0.2988	C	-1.9547	0.5404	0.4129
C	-1.2272	-0.2745	-0.6061	C	-1.013	-0.4494	-0.2243
C	-1.5254	-1.7538	-0.7216	C	-1.5274	-1.8383	-0.5322
C	-2.6366	-2.3875	0.0691	C	-2.9353	-2.2396	-0.1894
C	-4.0198	-1.9027	-0.3924	C	-3.9709	-1.4805	-1.033
C	-4.4146	-0.5323	0.1447	C	-4.2571	-0.0656	-0.5453
C	-3.9949	2.0589	0.0319	C	-3.3532	2.389	-0.3172
C	-2.7294	2.8696	-0.2071	C	-1.9519	2.9365	-0.0903
C	-1.6537	2.0334	0.4883	C	-1.3215	1.8923	0.8335
C	-5.5052	-0.4053	0.9209	C	-5.4974	0.2677	-0.1467
C	-0.1927	-1.2965	-0.1293	C	-0.4015	-1.6578	0.487
C	1.0382	-1.5231	-1.0062	C	0.9861	-2.1139	0.0337
C	0.096	-1.4632	1.3451	C	-0.6541	-1.8898	1.9588
C	2.0823	-0.4439	-0.9036	C	2.1268	-1.2694	0.5385
C	-0.2459	2.4088	0.0559	C	0.1956	1.9812	0.8659
C	3.3044	-0.6302	-0.3762	C	2.9568	-0.5781	-0.2622
C	4.4078	0.3926	-0.2124	C	4.1359	0.2805	0.1426
C	4.8727	0.4687	1.2627	C	5.4326	-0.1998	-0.5538
C	4.0716	1.7994	-0.7168	C	4.3727	0.3912	1.6518
H	-1.0432	0.2315	-1.5506	H	-0.438	0.0127	-1.0234
H	-1.5131	-2.1652	-1.7287	H	-1.2623	-2.2375	-1.5091
H	-2.1477	0.1181	1.3004	H	-2.4081	0.1141	1.3151
H	-3.4797	0.5752	-1.3912	H	-2.6867	0.8534	-1.6191
O	5.4734	-0.7668	1.6684	O	5.3388	-0.0271	-1.9727
O	5.521	-0.0964	-0.9784	O	3.8585	1.5994	-0.3565
H	-2.5353	-2.2384	1.1478	H	-3.1693	-2.1231	0.8724
H	-2.5754	-3.4707	-0.0941	H	-3.0296	-3.3113	-0.4045
H	-4.7577	-2.6489	-0.0701	H	-4.8998	-2.0658	-1.0245
H	-4.0691	-1.8808	-1.4885	H	-3.6499	-1.436	-2.0815
H	-4.8185	2.3874	-0.6094	H	-3.8553	2.8841	-1.1541
H	-4.3044	2.1651	1.0781	H	-3.9542	2.5382	0.5873
H	-2.804	3.8777	0.2121	H	-1.9698	3.932	0.3637
H	-2.5288	2.9582	-1.2816	H	-1.4094	3.0027	-1.0412
H	-1.7248	2.2502	1.5645	H	-1.6766	2.1068	1.8522
H	-5.8396	0.5493	1.3112	H	-5.7577	1.2654	0.1883
H	-6.1089	-1.2663	1.1941	H	-6.3057	-0.4583	-0.1453
H	0.7433	-1.5893	-2.0616	H	1.0214	-2.1596	-1.0628
H	1.4673	-2.5024	-0.7583	H	1.1453	-3.1448	0.3747
H	-0.783	-1.3711	1.9852	H	-1.6664	-1.6425	2.2825
H	0.5296	-2.4514	1.5328	H	-0.4848	-2.9431	2.2072
H	0.8142	-0.7077	1.6804	H	0.0283	-1.2837	2.5634
H	1.7847	0.5258	-1.2913	H	2.2636	-1.2614	1.616
H	0.4967	1.8013	0.5817	H	0.6159	1.2295	1.5406

H	-0.0422	3.4584	0.2944	H	0.5117	2.965	1.2295
H	-0.1014	2.2863	-1.0217	H	0.6326	1.8459	-0.1279
H	3.5533	-1.6224	0.0034	H	2.7891	-0.6265	-1.3391
H	5.641	1.24	1.3861	H	6.2905	0.3978	-0.2266
H	4.0433	0.694	1.941	H	5.6435	-1.2533	-0.3427
H	4.9411	2.461	-0.6249	H	5.1926	1.0874	1.8643
H	3.8146	1.7896	-1.7825	H	3.4931	0.7989	2.1637
H	3.2419	2.2455	-0.1583	H	4.6224	-0.5774	2.0978
H	6.0345	-1.0239	0.9088	H	4.9262	0.8536	-2.0822
H	5.1914	-0.2385	-1.8838	H	3.0031	1.8634	0.0263
**5-3**	X axis(Å)	Y axis(Å)	Z axis(Å)	**5-4**	X axis(Å)	Y axis(Å)	Z axis(Å)
C	-3.8748	-0.1897	-0.4056	C	3.4204	0.6936	-0.5852
C	-2.704	0.5721	0.2819	C	2.7226	-0.4775	0.1667
C	-1.4427	0.3855	-0.519	C	1.2821	-0.5648	-0.2624
C	-0.8207	-0.9905	-0.5935	C	0.3401	0.5561	0.112
C	-1.4323	-2.1516	0.1402	C	0.8189	1.7279	0.9225
C	-2.8022	-2.54	-0.4385	C	1.826	2.5905	0.1454
C	-3.9532	-1.6498	0.0158	C	3.2441	2.0314	0.1183
C	-5.0869	0.7151	-0.155	C	4.8413	0.1695	-0.8244
C	-4.5025	2.1072	-0.349	C	4.5966	-1.2897	-1.1817
C	-3.1852	2.0392	0.4268	C	3.5787	-1.7362	-0.1299
C	-4.9763	-2.1794	0.7094	C	4.2531	2.7368	0.6593
C	-0.0548	0.149	0.0782	C	0.1048	-0.8457	0.6741
C	1.1225	0.7447	-0.6912	C	-1.0607	-1.6372	0.0832
C	0.1465	0.1741	1.5759	C	0.3477	-1.1466	2.1347
C	2.3727	-0.0941	-0.6455	C	-2.4192	-1.154	0.5219
C	-2.2126	3.1407	0.0321	C	2.8443	-3.0088	-0.5252
C	3.5342	0.3242	-0.1138	C	-3.3029	-0.5661	-0.3031
C	4.8386	-0.4375	-0.0181	C	-4.6774	-0.0316	0.0363
C	5.9984	0.3708	-0.6494	C	-4.8031	1.4582	-0.3664
C	4.8133	-1.8491	-0.612	C	-5.1011	-0.2056	1.4978
H	-1.5079	0.9056	-1.472	H	1.205	-0.8923	-1.2966
H	-0.4827	-1.3091	-1.5771	H	-0.3328	0.8985	-0.6718
H	-2.5815	0.1815	1.2981	H	2.7977	-0.2953	1.2444
H	-3.7148	-0.1917	-1.4951	H	2.9645	0.8068	-1.5812
O	6.236	1.5755	0.0882	O	-4.7053	1.6088	-1.7876
O	5.1262	-0.5562	1.3852	O	-5.6032	-0.7665	-0.7812
H	-1.5207	-1.9768	1.2165	H	1.248	1.4338	1.8849
H	-0.7514	-3.0042	0.0259	H	-0.059	2.3428	1.1565
H	-3.0044	-3.5779	-0.1432	H	1.8347	3.5867	0.6069
H	-2.7659	-2.5385	-1.5353	H	1.484	2.7375	-0.887
H	-5.9061	0.5146	-0.8523	H	5.3525	0.7074	-1.6288
H	-5.4686	0.6186	0.8681	H	5.4588	0.2256	0.0796

H	-5.1672	2.8874	0.0344	H	5.5167	-1.8808	-1.1422
H	-4.3249	2.3008	-1.4138	H	4.1808	-1.3696	-2.1934
H	-3.4273	2.1912	1.489	H	4.1405	-1.9626	0.7883
H	-5.828	-1.5938	1.0369	H	5.2814	2.3937	0.6466
H	-4.9961	-3.2329	0.974	H	4.0796	3.697	1.1371
H	1.3153	1.7548	-0.3078	H	-0.9586	-2.692	0.3675
H	0.8549	0.8737	-1.7481	H	-1.0017	-1.6238	-1.0132
H	-0.7063	-0.1951	2.1478	H	1.2057	-0.6252	2.562
H	1.0071	-0.4428	1.8551	H	-0.525	-0.8646	2.7326
H	0.3403	1.1989	1.9107	H	0.5214	-2.2193	2.2721
H	2.2828	-1.0849	-1.0825	H	-2.6553	-1.3078	1.5709
H	-1.278	3.0582	0.5957	H	2.117	-3.2951	0.241
H	-2.645	4.1237	0.2484	H	3.5531	-3.8367	-0.6361
H	-1.9775	3.1168	-1.0365	H	2.3194	-2.9	-1.4793
H	3.5723	1.3298	0.3079	H	-3.0157	-0.428	-1.3466
H	6.932	-0.2014	-0.614	H	-5.7842	1.8538	-0.0814
H	5.7926	0.6339	-1.6922	H	-4.0306	2.075	0.1046
H	5.7702	-2.3569	-0.4428	H	-6.1304	0.142	1.6459
H	4.0538	-2.4725	-0.1255	H	-5.097	-1.2621	1.7905
H	4.6197	-1.8345	-1.6898	H	-4.4504	0.3516	2.1801
H	6.1818	1.2996	1.0257	H	-5.2466	0.8764	-2.1461
H	4.3442	-0.9708	1.792	H	-5.4466	-1.7096	-0.5954
**5-5**	X axis(Å)	Y axis(Å)	Z axis(Å)	**5-6**	X axis(Å)	Y axis(Å)	Z axis(Å)
C	3.7501	0.3133	-0.4179	C	-3.4546	0.6592	-0.4874
C	2.644	-0.4919	0.3249	C	-2.0911	0.5812	0.2637
C	1.3904	-0.5131	-0.5088	C	-1.1603	-0.3393	-0.4843
C	0.6384	0.7795	-0.7299	C	-1.5008	-1.8128	-0.5398
C	1.1131	2.0629	-0.1071	C	-2.7158	-2.3571	0.1595
C	2.4519	2.529	-0.7008	C	-4.0202	-1.8595	-0.4824
C	3.6735	1.8078	-0.1425	C	-4.4174	-0.445	-0.0777
C	5.0393	-0.436	-0.061	C	-3.8878	2.1185	-0.3044
C	4.6003	-1.8921	-0.1238	C	-2.5734	2.8711	-0.4518
C	3.2644	-1.8817	0.6225	C	-1.6124	2.0488	0.4087
C	4.6215	2.5029	0.5105	C	-5.5799	-0.2326	0.5638
C	-0.0287	-0.3594	0.0405	C	-0.2241	-1.3592	0.1675
C	-1.1233	-1.1398	-0.6843	C	1.0862	-1.6791	-0.5518
C	-0.2645	-0.2556	1.5294	C	-0.1049	-1.4375	1.6719
C	-2.4411	-0.419	-0.7643	C	2.1441	-0.6192	-0.4138
C	2.4153	-3.109	0.3259	C	-0.1525	2.3494	0.1111
C	-3.5791	-0.8532	-0.195	C	3.2987	-0.7856	0.255
C	-4.9322	-0.1697	-0.2495	C	4.4066	0.2335	0.4285
C	-5.404	0.2267	1.1691	C	5.7073	-0.2232	-0.2718
C	-5.9512	-1.0929	-0.9157	C	4.6656	0.4739	1.9177

H	1.5306	-1.1135	-1.4048	H	-0.8549	0.0965	-1.4327
H	0.2936	0.9651	-1.745	H	-1.3944	-2.2897	-1.512
H	2.4584	-0.0183	1.2953	H	-2.2667	0.2088	1.2794
H	3.6173	0.1929	-1.5044	H	-3.2792	0.5284	-1.5668
O	-4.5621	1.2587	1.7015	O	5.5342	-0.263	-1.6916
O	-4.8426	1.0197	-1.0394	O	4.0213	1.4802	-0.1668
H	1.1914	2.0045	0.9826	H	-2.727	-2.1408	1.2314
H	0.3542	2.827	-0.3164	H	-2.6796	-3.4501	0.0712
H	2.5432	3.6059	-0.5075	H	-4.8176	-2.5589	-0.1992
H	2.4434	2.4167	-1.7925	H	-3.9486	-1.9077	-1.5765
H	5.8514	-0.223	-0.7631	H	-4.6238	2.43	-1.052
H	5.3848	-0.2021	0.9527	H	-4.3045	2.3033	0.6925
H	5.3294	-2.5601	0.3448	H	-2.6537	3.9065	-0.1067
H	4.4685	-2.2057	-1.1665	H	-2.2544	2.8823	-1.501
H	3.4946	-1.9038	1.6979	H	-1.7905	2.3387	1.4549
H	5.5185	2.0412	0.9076	H	-5.9176	0.7558	0.8542
H	4.53	3.5739	0.6689	H	-6.2428	-1.0541	0.8208
H	-1.2421	-2.114	-0.193	H	0.9041	-1.8187	-1.6254
H	-0.8099	-1.3627	-1.7126	H	1.457	-2.646	-0.1879
H	0.5352	0.2483	2.0748	H	-1.0439	-1.2737	2.2032
H	-1.1858	0.3005	1.7312	H	0.265	-2.4251	1.9678
H	-0.3698	-1.256	1.9628	H	0.6029	-0.6862	2.0374
H	-2.4424	0.502	-1.3444	H	1.9367	0.3155	-0.9276
H	1.4637	-3.0662	0.865	H	0.5052	1.7545	0.7518
H	2.9374	-4.0175	0.6454	H	0.0651	3.4054	0.3048
H	2.2049	-3.2137	-0.743	H	0.1021	2.1517	-0.9345
H	-3.5535	-1.7867	0.366	H	3.4859	-1.7507	0.7252
H	-6.4155	0.645	1.1321	H	6.5164	0.4894	-0.0776
H	-5.4045	-0.6222	1.8612	H	6.03	-1.2134	0.0663
H	-6.9235	-0.5953	-1.0088	H	5.4196	1.2568	2.0595
H	-5.6402	-1.3521	-1.9349	H	3.7585	0.8251	2.4241
H	-6.0918	-2.0211	-0.3513	H	5.0111	-0.4346	2.4229
H	-3.6586	0.8948	1.745	H	4.9666	0.5086	-1.8912
H	-4.5823	1.7275	-0.4181	H	3.2247	1.7789	0.306
**6-1**	X axis(Å)	Y axis(Å)	Z axis(Å)	**6-2**	X axis(Å)	Y axis(Å)	Z axis(Å)
C	-2.189	0.3248	-0.5909	C	-2.2277	0.2818	-0.5561
C	-0.81	0.4091	0.1112	C	-0.8298	0.4081	0.0994
C	0.2153	-0.4779	-0.5438	C	0.2065	-0.4431	-0.5858
C	0.0931	-1.9836	-0.5232	C	0.1269	-1.9508	-0.563
C	-1.1051	-2.6427	0.0995	C	-1.0288	-2.6472	0.0981
C	-2.4139	-2.2479	-0.6006	C	-2.3708	-2.2963	-0.5611
C	-2.9662	-0.9067	-0.1322	C	-2.9508	-0.9735	-0.0748
C	-2.8164	1.7264	-0.3863	C	-2.8909	1.6636	-0.3247

C	-1.8118	2.5276	0.44	C	-1.8826	2.4957	0.4667
C	-0.4534	1.9124	0.0962	C	-0.5217	1.921	0.0722
C	-4.0733	-0.8827	0.6308	C	-4.0346	-0.9854	0.7213
C	1.226	-1.302	0.2507	C	1.2669	-1.239	0.174
C	2.6352	-1.3544	-0.3338	C	2.654	-1.2973	-0.4631
C	1.2212	-1.3284	1.7641	C	1.3116	-1.2862	1.6862
C	3.3997	-0.0639	-0.1457	C	3.5626	-0.1293	-0.1623
C	0.0783	2.4738	-1.2268	C	-0.0527	2.4971	-1.2679
O	0.4823	2.2206	1.1244	O	0.4504	2.2505	1.0594
O	2.9623	1.0034	0.2567	O	4.782	-0.1498	-0.2313
H	0.5175	-0.0834	-1.5101	H	0.4685	-0.0392	-1.5598
H	0.3571	-2.498	-1.4448	H	0.3742	-2.4565	-1.4944
H	-0.9373	0.116	1.1607	H	-0.9125	0.112	1.1526
H	-2.0445	0.2098	-1.6754	H	-2.1163	0.1736	-1.6451
O	4.6936	-0.1817	-0.505	O	2.9308	1.0222	0.1447
H	-1.188	-2.4519	1.1729	H	-1.0845	-2.457	1.1734
H	-0.9696	-3.7267	-0.0033	H	-0.8614	-3.7265	-0.0078
H	-3.1513	-3.0365	-0.4007	H	-3.0755	-3.1084	-0.3385
H	-2.2835	-2.2285	-1.6898	H	-2.2751	-2.2733	-1.6538
H	-2.9408	2.204	-1.3668	H	-3.0661	2.1382	-1.2989
H	-3.802	1.7414	0.0813	H	-3.8587	1.6473	0.1789
H	-2.0252	2.4104	1.5105	H	-1.9742	3.5664	0.2555
H	-1.8632	3.6004	0.2259	H	-2.053	2.3717	1.544
H	-4.5122	0.0297	1.0154	H	-4.4922	-0.0882	1.1194
H	-4.578	-1.8004	0.921	H	-4.5006	-1.9192	1.0251
H	3.1926	-2.1653	0.1486	H	3.155	-2.2064	-0.1099
H	2.5973	-1.56	-1.4101	H	2.574	-1.3651	-1.5546
H	0.2261	-1.2958	2.2105	H	0.3309	-1.261	2.1642
H	1.7029	-2.2458	2.1197	H	1.8065	-2.2072	2.0132
H	1.7789	-0.4775	2.1677	H	1.8785	-0.4401	2.0863
H	1.0885	2.1089	-1.4389	H	0.9628	2.1672	-1.5106
H	0.1645	3.5653	-1.1692	H	-0.0025	3.5911	-1.2145
H	-0.563	2.2282	-2.0783	H	-0.7121	2.2278	-2.0981
H	1.3363	1.8031	0.8787	H	1.253	1.7342	0.8393
H	5.0723	0.708	-0.343	H	3.6718	1.6557	0.2685
**6-3**	X axis(Å)	Y axis(Å)	Z axis(Å)	**6-4**	X axis(Å)	Y axis(Å)	Z axis(Å)
C	2.2329	0.5502	-0.6296	C	2.2296	0.5463	-0.6318
C	1.2067	-0.3584	0.1072	C	1.2009	-0.3597	0.1062
C	-0.1545	-0.2174	-0.5296	C	-0.159	-0.2132	-0.5328
C	-0.8941	1.0951	-0.4161	C	-0.8981	1.099	-0.4156
C	-0.2905	2.2554	0.3225	C	-0.2914	2.2569	0.3231
C	0.9588	2.7931	-0.3921	C	0.9583	2.7912	-0.393
C	2.2251	1.9887	-0.1257	C	2.2251	1.9851	-0.1286

C	3.5419	-0.2412	-0.5235	C	3.5405	-0.2419	-0.5214
C	3.0871	-1.6727	-0.7775	C	3.0933	-1.6784	-0.7574
C	1.8077	-1.7963	0.0579	C	1.8011	-1.7986	0.0607
C	3.2676	2.5751	0.4894	C	3.2699	2.5747	0.48
C	-1.4718	-0.1536	0.2473	C	-1.4746	-0.1511	0.2464
C	-2.684	-0.799	-0.4229	C	-2.6864	-0.7986	-0.4218
C	-1.4768	-0.3083	1.7506	C	-1.4738	-0.3153	1.7491
C	-3.9898	-0.0838	-0.1721	C	-3.9922	-0.0848	-0.1676
C	0.9068	-2.9166	-0.4535	C	0.902	-2.9086	-0.4747
O	2.2025	-2.1649	1.3899	O	2.1466	-2.1978	1.3981
O	-4.1625	1.0023	0.3538	O	-4.1638	1.0085	0.3439
H	-0.1634	-0.6458	-1.5292	H	-0.1703	-0.6421	-1.5318
H	-1.3877	1.4516	-1.318	H	-1.3947	1.4569	-1.3152
H	1.1728	-0.0424	1.1559	H	1.1642	-0.0427	1.1543
H	1.9767	0.5929	-1.6994	H	1.975	0.5869	-1.702
O	-5.0445	-0.7884	-0.6338	O	-5.0492	-0.7976	-0.6115
H	-0.0521	2.0225	1.3643	H	-0.0522	2.0233	1.3646
H	-1.045	3.0511	0.3537	H	-1.0438	3.0547	0.3555
H	1.1078	3.8301	-0.0638	H	1.1093	3.8283	-0.0659
H	0.7898	2.8399	-1.4754	H	0.7882	2.8376	-1.4762
H	4.285	0.0857	-1.2576	H	4.28	0.0794	-1.2617
H	3.9866	-0.1701	0.476	H	3.9912	-0.1567	0.474
H	3.8612	-2.3879	-0.481	H	3.8655	-2.3868	-0.4406
H	2.8765	-1.8048	-1.8457	H	2.8985	-1.8259	-1.8266
H	4.1993	2.0571	0.6883	H	4.2064	2.0641	0.673
H	3.2224	3.6091	0.8199	H	3.2229	3.6104	0.8064
H	-2.5422	-0.8388	-1.5097	H	-2.5474	-0.8377	-1.5089
H	-2.7782	-1.8286	-0.058	H	-2.7781	-1.8284	-0.0571
H	-0.6172	0.1431	2.2487	H	-0.6234	0.1513	2.2491
H	-2.3713	0.1509	2.1828	H	-2.377	0.1231	2.185
H	-1.4815	-1.3714	2.0148	H	-1.457	-1.3798	2.0066
H	-0.0024	-2.9974	0.1525	H	-0.0141	-2.9928	0.1203
H	1.4156	-3.8844	-0.3725	H	1.4055	-3.8799	-0.3995
H	0.6215	-2.7758	-1.5003	H	0.6301	-2.7555	-1.5233
H	1.4006	-2.1751	1.9409	H	2.7131	-1.5109	1.789
H	-5.8148	-0.2192	-0.4242	H	-5.8187	-0.229	-0.398
**6-5**	X axis(Å)	Y axis(Å)	Z axis(Å)				
C	2.2244	0.5584	-0.6278				
C	1.2019	-0.3551	0.1102				
C	-0.1588	-0.2133	-0.5305				
C	-0.8993	1.0987	-0.4137				
C	-0.2973	2.2563	0.3307				
C	0.9515	2.7998	-0.3803				

C	2.2197	1.9955	-0.1211				
C	3.5346	-0.2328	-0.5384				
C	3.0766	-1.6627	-0.7952				
C	1.8097	-1.7903	0.0599				
C	3.2646	2.5805	0.4913				
C	-1.4764	-0.1525	0.2455				
C	-2.6858	-0.7999	-0.4257				
C	-1.4784	-0.3166	1.7483				
C	-3.9941	-0.0913	-0.1705				
C	0.8981	-2.9119	-0.4297				
O	2.2074	-2.1338	1.3985				
O	-4.1707	1.0032	0.3366				
H	-0.1665	-0.6405	-1.53				
H	-1.3931	1.4578	-1.3142				
H	1.166	-0.0416	1.1596				
H	1.9611	0.606	-1.6958				
O	-5.0488	-0.811	-0.6094				
H	-0.0587	2.0191	1.3715				
H	-1.053	3.0508	0.3656				
H	1.0995	3.8348	-0.0452				
H	0.7817	2.8537	-1.4632				
H	4.2699	0.0978	-1.2787				
H	3.9896	-0.1661	0.4567				
H	3.8564	-2.3786	-0.5164				
H	2.8482	-1.7892	-1.8604				
H	4.196	2.0614	0.6876				
H	3.2194	3.6132	0.8256				
H	-2.5459	-0.8352	-1.5128				
H	-2.7749	-1.8314	-0.0647				
H	-0.6246	0.1423	2.2494				
H	-2.3784	0.1299	2.1829				
H	-1.4713	-1.3812	2.0062				
H	0.0055	-2.9934	0.2002				
H	1.4084	-3.8802	-0.3648				
H	0.587	-2.7714	-1.4691				
H	2.6575	-2.9952	1.3687				
H	-5.8196	-0.2437	-0.3965				
**7-1**	X axis(Å)	Y axis(Å)	Z axis(Å)	**7-2**	X axis(Å)	Y axis(Å)	Z axis(Å)
C	1.9	0.358	-0.6333	C	1.8981	0.3215	-0.638
C	0.7583	-0.366	0.1371	C	0.7423	-0.3646	0.1475
C	-0.5777	0.0086	-0.454	C	-0.5857	0.029	-0.4505
C	-1.0863	1.4226	-0.3217	C	-1.0555	1.4588	-0.3424
C	-0.2706	2.4687	0.3812	C	-0.2175	2.4944	0.35

C	1.0256	2.783	-0.3809	C	1.0869	2.7705	-0.4128
C	2.1456	1.7793	-0.1401	C	2.1817	1.7414	-0.1625
C	3.0619	-0.6391	-0.5557	C	3.0353	-0.7036	-0.5592
C	2.3698	-1.9749	-0.7928	C	2.3069	-2.0242	-0.7714
C	1.1064	-1.885	0.0713	C	1.0591	-1.891	0.109
C	3.2873	2.1851	0.4437	C	3.335	2.1248	0.4137
C	-1.8445	0.2813	0.3738	C	-1.8478	0.3506	0.3678
C	-1.8111	0.1412	1.8766	C	-1.8298	0.2523	1.8741
C	-3.129	-0.1622	-0.2521	C	-3.1638	-0.0027	-0.2513
C	0.0207	-2.8369	-0.4217	C	-0.0528	-2.8311	-0.3453
O	1.4647	-2.3162	1.3951	O	1.4296	-2.303	1.4357
O	-3.3851	-1.3201	-0.5348	O	-4.1035	0.7674	-0.3494
H	-0.7037	-0.4202	-1.446	H	-0.7186	-0.4114	-1.4363
H	-1.55	1.8517	-1.2082	H	-1.5073	1.8872	-1.2358
H	0.8113	-0.0533	1.1863	H	0.8034	-0.034	1.1906
H	1.6247	0.4395	-1.6961	H	1.6181	0.3986	-1.7
O	-4.0015	0.8414	-0.4589	O	-3.2311	-1.2778	-0.6783
H	-0.0377	2.2067	1.4172	H	0.0071	2.2386	1.3895
H	-0.8785	3.3808	0.4276	H	-0.8045	3.4206	0.3849
H	1.3574	3.7833	-0.0731	H	1.4428	3.7649	-0.113
H	0.8312	2.8471	-1.4591	H	0.895	2.8299	-1.4917
H	3.8307	-0.44	-1.3088	H	3.8006	-0.5338	-1.323
H	3.5365	-0.6428	0.4326	H	3.5207	-0.7065	0.4238
H	3.0209	-2.8087	-0.5111	H	2.9407	-2.8707	-0.4877
H	2.1161	-2.0706	-1.8556	H	2.0358	-2.1265	-1.8292
H	4.1243	1.5189	0.6226	H	4.1551	1.4397	0.5988
H	3.4254	3.2131	0.7669	H	3.4999	3.1528	0.7241
H	-2.6304	0.7167	2.3208	H	-2.5964	0.9095	2.2988
H	-1.9474	-0.9097	2.1543	H	-2.0579	-0.7745	2.1801
H	-0.8858	0.4837	2.3424	H	-0.8784	0.5239	2.3338
H	-0.8742	-2.7721	0.2068	H	-0.9328	-2.7387	0.3003
H	0.3647	-3.876	-0.3572	H	0.2735	-3.875	-0.2687
H	-0.2625	-2.6427	-1.4605	H	-0.3532	-2.6505	-1.3816
H	0.6615	-2.2779	1.9429	H	0.6439	-2.2101	2.002
H	-4.7782	0.398	-0.8571	H	-4.1375	-1.351	-1.0419
**7-3**	X axis(Å)	Y axis(Å)	Z axis(Å)	**7-4**	X axis(Å)	Y axis(Å)	Z axis(Å)
C	1.8884	0.3748	-0.6398	C	1.8884	0.3358	-0.6419
C	0.7573	-0.356	0.1409	C	0.7404	-0.3566	0.1504
C	-0.5811	0.0063	-0.4539	C	-0.5898	0.0295	-0.4496
C	-1.1007	1.4154	-0.315	C	-1.0683	1.4558	-0.3333
C	-0.2908	2.4618	0.3949	C	-0.2335	2.4905	0.3647
C	1.0016	2.793	-0.3681	C	1.0674	2.7798	-0.4002
C	2.1323	1.7959	-0.1461	C	2.1711	1.7556	-0.1661

C	3.0545	-0.6191	-0.5861	C	3.029	-0.6867	-0.5811
C	2.3607	-1.9568	-0.8113	C	2.3002	-2.0087	-0.7865
C	1.1205	-1.871	0.0869	C	1.0686	-1.8802	0.1181
C	3.2792	2.2066	0.4239	C	3.3288	2.1427	0.3985
C	-1.8513	0.2631	0.3723	C	-1.8535	0.3374	0.3701
C	-1.8165	0.106	1.8736	C	-1.8311	0.2223	1.8756
C	-3.1325	-0.1787	-0.2602	C	-3.1687	-0.0165	-0.2493
C	0.0225	-2.833	-0.3569	C	-0.0535	-2.8242	-0.3023
O	1.5065	-2.2622	1.4162	O	1.4537	-2.2598	1.4511
O	-3.3604	-1.3199	-0.6243	O	-4.1246	0.7383	-0.3051
H	-0.7018	-0.4225	-1.4462	H	-0.7192	-0.4088	-1.4363
H	-1.5674	1.8464	-1.1987	H	-1.5232	1.8869	-1.2234
H	0.8089	-0.0355	1.1878	H	0.8009	-0.0223	1.1922
H	1.5999	0.4602	-1.6989	H	1.5988	0.4164	-1.7012
O	-4.0377	0.8103	-0.3842	O	-3.2184	-1.272	-0.7345
H	-0.0535	2.1931	1.4281	H	-0.0044	2.2285	1.4016
H	-0.9055	3.3688	0.4504	H	-0.8254	3.4132	0.4082
H	1.3271	3.7922	-0.0501	H	1.4187	3.7735	-0.0928
H	0.8016	2.8681	-1.4445	H	0.8704	2.8483	-1.4777
H	3.8073	-0.4171	-1.3545	H	3.783	-0.5134	-1.3554
H	3.5491	-0.622	0.3922	H	3.5283	-0.6904	0.3948
H	3.0223	-2.7888	-0.5499	H	2.9422	-2.8541	-0.5192
H	2.0778	-2.0505	-1.8667	H	2.0083	-2.1078	-1.839
H	4.1215	1.5446	0.591	H	4.1533	1.4607	0.574
H	3.4141	3.2342	0.7498	H	3.4911	3.1704	0.7114
H	-2.6448	0.6639	2.3238	H	-2.6082	0.8614	2.3087
H	-1.9375	-0.9499	2.139	H	-2.0415	-0.8116	2.1702
H	-0.8974	0.4581	2.3442	H	-0.8845	0.5061	2.3376
H	-0.8448	-2.7701	0.3091	H	-0.9128	-2.7329	0.3708
H	0.3738	-3.8703	-0.3045	H	0.2759	-3.8679	-0.2374
H	-0.3039	-2.6465	-1.3843	H	-0.3848	-2.6457	-1.3294
H	1.7738	-3.197	1.3886	H	1.7184	-3.1955	1.4331
H	-4.8073	0.369	-0.7977	H	-4.1303	-1.345	-1.0831
**7-5**	X axis(Å)	Y axis(Å)	Z axis(Å)	**7-6**	X axis(Å)	Y axis(Å)	Z axis(Å)
C	1.9261	0.3215	-0.6454	C	1.9335	0.264	-0.6411
C	0.7653	-0.3613	0.1259	C	0.7435	-0.3644	0.1313
C	-0.5745	-0.0013	-0.4621	C	-0.5796	0.04	-0.4651
C	-1.0799	1.419	-0.3896	C	-1.0322	1.4784	-0.4104
C	-0.2632	2.5009	0.257	C	-0.1759	2.537	0.2236
C	1.0594	2.7488	-0.4849	C	1.1567	2.7237	-0.518
C	2.1474	1.7417	-0.1309	C	2.2052	1.6819	-0.1451
C	3.0957	-0.6894	-0.5789	C	3.0638	-0.7896	-0.5527
C	2.6157	-1.8326	0.3099	C	2.5327	-1.9056	0.3421

C	1.1001	-1.8677	0.1007	C	1.0188	-1.8829	0.1213
C	3.2073	2.1426	0.5931	C	3.2763	2.0538	0.5779
C	-1.8278	0.3119	0.3715	C	-1.8216	0.409	0.3628
C	-1.7781	0.2563	1.8802	C	-1.7842	0.3931	1.8727
C	-3.1128	-0.1816	-0.2124	C	-3.1375	0.0011	-0.2177
C	0.734	-2.6215	-1.1807	C	0.6331	-2.6329	-1.1565
O	0.4894	-2.5336	1.2018	O	0.3715	-2.5152	1.2214
O	-3.3566	-1.3595	-0.4118	O	-4.0567	0.7668	-0.4494
H	-0.7218	-0.4697	-1.4323	H	-0.7402	-0.4309	-1.4317
H	-1.5552	1.8052	-1.2895	H	-1.4964	1.872	-1.3132
H	0.8075	-0.0401	1.1741	H	0.7944	-0.0351	1.1765
H	1.6645	0.4155	-1.7097	H	1.6819	0.3527	-1.7083
O	-3.9948	0.7975	-0.4834	O	-3.2249	-1.3237	-0.4471
H	-0.0634	2.3091	1.315	H	0.0141	2.3518	1.2846
H	-0.8581	3.4223	0.2245	H	-0.7349	3.48	0.1772
H	1.4006	3.7611	-0.2315	H	1.5352	3.7262	-0.279
H	0.9023	2.7469	-1.5708	H	1.0024	2.7112	-1.6042
H	3.2859	-1.0753	-1.5889	H	3.2496	-1.1922	-1.557
H	4.0485	-0.2883	-0.2289	H	4.0273	-0.4213	-0.1962
H	3.1036	-2.7813	0.0625	H	2.7705	-1.6935	1.3926
H	2.8533	-1.6201	1.3604	H	2.9849	-2.8748	0.1065
H	4.0048	1.4759	0.8986	H	4.0479	1.3631	0.8962
H	3.3042	3.1713	0.9301	H	3.4094	3.083	0.9008
H	-2.58	0.8743	2.2988	H	-2.5385	1.0817	2.2687
H	-1.9346	-0.7731	2.2201	H	-2.0202	-0.6117	2.2394
H	-0.8405	0.6039	2.3164	H	-0.8244	0.6786	2.3057
H	-0.3518	-2.7314	-1.2782	H	-0.4553	-2.7029	-1.2607
H	1.1342	-3.6417	-1.1504	H	0.9942	-3.6672	-1.1152
H	1.1163	-2.1323	-2.0814	H	1.0396	-2.1662	-2.0585
H	-0.4737	-2.5151	1.0548	H	-0.5884	-2.4206	1.0839
H	-4.7719	0.3222	-0.842	H	-4.1282	-1.4329	-0.8104

Reference

(1) M. J. Frisch, G. W. Trucks, H. B. Schlegel, G. E. Scuseria, M. A. Robb, J. R. Cheeseman, G. Scalmani, V. Barone, B. Mennucci, G. A. Petersson, H. Nakatsuji, M. Caricato, X. Li, H. P. Hratchian, A. F. Izmaylov, J. Bloino, G. Zheng, J. L. Sonnenberg, M. Hada, M. Ehara, K. Toyota, R. Fukuda, J. Hasegawa, M. Ishida, T. Nakajima, Y. Honda, O. Kitao, H. Nakai, T. Vreven, J. A. Montgomery, J. E. Peralta, F. Ogliaro, M. Bearpark, J. J. Heyd, E. Brothers, K. N. Kudin, V. N. Staroverov, R.

Kobayashi, J. Normand, K. Raghavachari, A. Rendell, J. C. Burant, S. S. Iyengar, J. Tomasi, M. Cossi, N. Rega, J. M. Millam, M. Klene, J. E. Knox, J. B. Cross, V. Bakken, C. Adamo, J. Jaramillo, R. Gomperts, R. E. Stratmann, O. Yazyev, A. J. Austin, R. Cammi, C. Pomelli, J. W. Ochterski, R. L. Martin, K. Morokuma, V. G. Zakrzewski, G. A. Voth, P. Salvador, J. J. Dannenberg, S. Dapprich, A. D. Daniels, O. Farkas, J. B. Foresman, J. V. Ortiz, J. Cioslowski, D. J. Fox, Gaussian, Inc., Wallingford CT, 2009.
